# Medical Image Encryption via CSPRNG–HKDF Key Scheduling, Fractional-Order Modified Chen Hyperchaos, and Dynamic DNA–ARX Diffusion

**DOI:** 10.3390/s26196285

**Published:** 2026-10-03

**Authors:** Kaan Doğan Erdoğan, Nurettin Doğan

**Affiliations:** 1 Department of Property Protection and Security, Selcuk University, Konya 42400, Türkiye; kaan.erdogan@selcuk.edu.tr; 2 Faculty of Technology, Department of Computer Engineering, Selcuk University, Konya 42130, Türkiye

**Keywords:** medical image encryption, fractional-order systems, hyperchaotic Chen system, chaos-based cryptography, dynamic DNA encoding, bit-plane permutation, HKDF key derivation, ARX diffusion

## Abstract

Protecting medical images requires encryption methods that combine cryptographic key management with validated nonlinear transformations and effective diffusion mechanisms. This study proposes an encryption-instance-specific framework based on a CSPRNG-generated 256-bit session secret, HMAC-SHA-512-based HKDF with public NOAA/SWPC temporal context, a modified four-dimensional fractional-order Chen system, adaptive bit-plane permutation, position-dependent dynamic DNA transformation, and SPARKLE256-based two-pass ARX-duplex diffusion. The fractional operating region was evaluated through boundedness, Lyapunov-spectrum, bifurcation, phase-portrait, and modified 0–1 chaos analyses. All 10,000 sampled parameter triplets remained finite and bounded, all 1000 Lyapunov-grid configurations satisfied the hyperchaos criterion, and the minimum second Lyapunov exponent was 0.0546; the modified 0–1 test accepted all 35 representative cases with a median *K* value of 0.9971. For encrypted standard benchmark and medical images, entropy ranged from 7.9966 to 7.9978 bits. Across the 11 test images, the mean RGB NPCR and UACI values were 99.6115% and 33.4546%, respectively, while the corresponding grayscale means were 99.5999% and 33.4608%. Under NIST SP 800-22, the fractional modified Chen keystream satisfied 187 of 188 subtest rows (99.47%), while the operational ciphertext satisfied 185 of 188 rows (98.40%). In the 111-row Dieharder evaluation, the keystream produced 109 passed, 2 weak, and 0 failed outcomes, whereas the ciphertext produced 106 passed, 5 weak, and 0 failed outcomes. The optimized CPU implementation encrypted a 256 × 256 RGB image in 1356.327 ms, representing a 74.26-fold speedup relative to the 100,722.174 ms pure-Python 3.10.20 baseline. Overall, the results demonstrate strong statistical and differential characteristics while showing that the fractional-order chaotic generator remains the principal computational component of the proposed framework.

## 1. Introduction

The rapid digitalization of healthcare and the growing use of telemedicine have substantially increased the transmission, storage, and exchange of medical images across networked environments. Because these images contain sensitive diagnostic information and are often associated with patient records, their protection during transmission and storage has become an important requirement in modern healthcare systems [1,2]. Regulatory requirements such as the Health Insurance Portability and Accountability Act (HIPAA) Security Rule further reinforce the need to protect electronic protected health information (ePHI) through appropriate administrative, physical, and technical safeguards that ensure its confidentiality, integrity, and availability [3]. In this context, encryption provides a principal mechanism for protecting the confidentiality of medical-image data, while integrity and authenticity may require complementary protection mechanisms [1].

Digital images exhibit distinctive structural characteristics, including strong inter-pixel correlation, high data redundancy, and large data volume. These properties have motivated the development of encryption methods that explicitly consider the organization and statistical structure of image data. Standard cryptographic algorithms such as AES continue to provide a strong security foundation and remain widely used for image protection; however, image-encryption research has also explored specialized structures designed to address spatial redundancy and inter-pixel dependencies [4]. Complementary image-protection approaches have also been investigated outside the encryption domain. For example, Abdallah et al. proposed a block-based watermarking scheme that applies the fast Hadamard transform (FHT) to image blocks and embeds the singular values of the watermark image, obtained by singular value decomposition (SVD), into the DC components of the transformed blocks, thereby improving robustness against common image-processing attacks [5]. Such watermarking schemes primarily support ownership verification and related content-protection objectives while preserving the perceptual content of the host image; they therefore complement, rather than replace, encryption methods that conceal the image content. Within the encryption domain, a foundational chaos-based image-encryption architecture combines key-dependent pixel permutation with diffusion so that spatial information is rearranged and local plaintext changes propagate through the encrypted image [6]. Such methods are commonly assessed in terms of key sensitivity, resistance to statistical and differential attacks, computational efficiency, scalability, and encryption speed [4]. These structural and practical considerations have encouraged the development of image-oriented encryption architectures beyond conventional block-cipher processing. Among them, chaos-based methods have received considerable attention because chaotic systems exhibit pseudo-random behavior, sensitivity to initial conditions, and ergodicity, which are useful properties for constructing nonlinear transformations and pseudorandom sequences for image encryption [7].

Chaotic systems exhibit properties that are useful for cryptographic sequence generation, including sensitivity to initial conditions and system parameters, ergodicity, pseudo-random behavior, and deterministic unpredictability. Hyperchaotic systems extend this behavior by exhibiting two or more positive Lyapunov exponents, corresponding to simultaneous divergence along multiple phase-space directions and providing a richer dynamical structure for pseudorandom sequence generation [7,8]. Four-dimensional Chen-type hyperchaotic systems have consequently been employed in image-encryption architectures as nonlinear generators, with their multiple state variables and control parameters providing additional flexibility for key-controlled trajectory generation [7,9]. However, the use of a higher-dimensional or hyperchaotic system does not by itself establish cryptographic superiority; its admissible operating region and the statistical properties of the sequences generated under the actual numerical implementation must also be validated.

Fractional-order formulations introduce a qualitatively different extension by replacing instantaneous integer-order state evolution with nonlocal, history-dependent dynamics. Fractional derivatives retain contributions from previous states, while the derivative order provides an additional dynamical degree of freedom that can be controlled independently of the original system parameters [8,10,11]. This distinction is structural rather than merely an increase in the number of tunable parameters. In the Grünwald–Letnikov realization adopted in this work, the integer-order limit *α* = 1 reduces to a single-step four-dimensional recurrence, whereas *α* < 1 retains a finite history of previous states and therefore changes the effective state evolution of the generator. Fractional-order Chen systems have previously been employed for image-encryption and pseudorandom-sequence generation [7,9], but the present work does not assume that fractional-order chaos is universally superior to its integer-order counterpart. Indeed, comparative evaluations of different fractional-order chaotic systems show that no single model consistently dominates all image-encryption metrics [12]. The motivation here is therefore dynamical rather than based on marginal improvements in downstream ciphertext metrics: the fractional formulation introduces history dependence and an independently key-controlled order parameter, and the operating-region analysis in Section 3.3 shows that the second Lyapunov exponent reaches its strongest values within the fractional band rather than at the integer-order limit. These additional dynamical characteristics are obtained at a measurable computational cost, because numerical evaluation of fractional derivatives requires accumulation over previous states [10]. Accordingly, the fractional-order Chen generator is adopted because it provides memory-dependent state evolution and an additional order-sensitive degree of freedom, despite its higher computational cost. This choice is not intended to imply universal superiority over integer-order chaotic systems.

A central challenge in chaos-based image encryption is to disrupt the strong spatial correlation among adjacent pixels through effective permutation and diffusion operations. Bit-plane decomposition converts pixel values into their binary representations and separates an image into multiple binary layers, enabling encryption operations to be performed at the bit level rather than exclusively at the pixel level [13]. This finer representation provides additional flexibility for rearranging image information; however, the resulting bit planes are not equivalent in their contribution to the represented image. Higher-order bit planes generally contain more visually significant information, while correlations may also remain among the decomposed planes [13,14]. Recent approaches have therefore explored weighted and cross-bit-plane processing rather than treating all planes identically. Wen et al. [13] employed information-weighted bit-plane processing together with plaintext-associated chaotic control, whereas Liu et al. [15] performed cross-permutation between high- and low-order bit planes and used plaintext-related key generation. Such designs illustrate the use of input-dependent control to vary encryption parameters between images and to address known-plaintext and chosen-plaintext attack scenarios [14,15]. In the proposed method, rather than deriving permutation controls from the plaintext, each bit plane is assigned a reversible scan trajectory through a key-schedule-driven selector. This provides a finer-grained means of diversifying the permutation structure across different bit-plane significance levels while keeping the permutation controls dependent on secret key material.

Deoxyribonucleic acid (DNA) encoding represents binary image data using the four biological bases, adenine, thymine, cytosine, and guanine, through eight coding rules that satisfy Watson–Crick complementarity [16]. DNA-domain algebraic operations such as addition, subtraction, and XOR can subsequently be incorporated into image-encryption transformations [17]. However, conventional DNA-based schemes may employ a fixed coding rule or reuse the same coding configuration across image elements. Chai et al. [17] noted that such globally reused encoding rules can reduce resistance to statistical, known-plaintext, and chosen-plaintext attacks. Dynamic DNA coding addresses this limitation by varying the coding configuration across the image rather than applying a single global rule. Afify et al. [18] categorized these strategies according to the granularity of rule selection, with coding rules applied at the image, block, row, pixel, or two-bit level. Accordingly, the proposed scheme derives separate DNA control fields through the labeled CSPRNG–HKDF key schedule and performs position-dependent selection among the eight Watson–Crick-compliant rules. This design provides fine-grained transformation diversity without deriving secret material directly from the plaintext.

Diffusion mechanisms are used to propagate local variations in the plaintext throughout the encrypted data and thereby reduce the preservation of structural relationships. Addition, Rotation, and XOR operations, collectively referred to as ARX operations, provide a compact means of constructing diffusion transformations using simple integer operations. Zhang et al. [19] incorporated ARX-based round functions into a generalized Feistel structure and reported strong diffusion and avalanche behavior with relatively few rounds, together with flexible hardware serialization for resource-constrained implementations. Suzgen et al. [20], in contrast, integrated ARX with memristive chaos, DNA operations, and 3DES in a multilayer medical image encryption framework in which the R, G, and B channels are processed independently. In the proposed method, the DNA-transformed R, G, and B data are instead concatenated into a single sequence and processed by a SPARKLE256-based two-pass ARX-duplex construction. The forward and backward duplex passes use independently derived seeds and non-overlapping chaotic diffusion streams, with a deterministic inter-pass mask applied between the two traversals. Because the duplex state is not reinitialized at channel boundaries, dependencies can propagate across the serialized color components in both traversal directions.

A fundamental vulnerability in some chaos-based image encryption algorithms lies in the use of weak, static, or repeatedly reused key and permutation configurations. When an encryption process behaves essentially as a permutation-only cipher, an adversary may recover the underlying permutation from known plaintext or chosen plaintext pairs and subsequently use the recovered structure as an equivalent decryption key for ciphertexts produced under the same configuration [21]. Avoiding this exposure requires more than increasing the complexity of the chaotic system or repeatedly applying permutation operations. A more structured design should begin with secret material generated by a cryptographically secure random bit generator [22] and use a key-derivation function to derive distinct purpose-specific fields for different cipher functions [23]. In HKDF, application- and context-specific information can be incorporated through the info input to bind the derived keying material to its intended use and distinguish derivations performed under different contexts. More generally, explicit purpose labels and distinct context information provide a systematic basis for key separation and context binding when multiple cryptographic fields are derived from common secret material [24].

To address these limitations, this study introduces a session-specific key-derivation mechanism based on a confidential 256-bit session secret *K_s_*, independently generated for each encryption instance by a cryptographically secure pseudorandom number generator (CSPRNG). The selected Begin and Max timestamps obtained from NOAA’s Space Weather Prediction Center (NOAA/SWPC) are canonically encoded and used solely as public, non-secret session information. This use of NOAA/SWPC data is an application-specific design choice of the proposed method; the timestamps are not treated as secret key material and are not credited with cryptographic entropy. Accordingly, the confidentiality of the derived encryption configuration depends on *K_s_*, whereas the NOAA/SWPC values provide a reproducible and externally verifiable temporal binding for the corresponding encryption instance.

The confidential session secret *K_s_* is supplied as the input keying material to an HMAC-SHA-512-based HKDF following the Extract-and-Expand construction defined in RFC 5869 [23]. The canonically encoded NOAA/SWPC values are incorporated as public salt information in the extraction stage. The resulting pseudorandom key is subsequently expanded through separate HKDF-Expand invocations using function-specific labels and context information. These domain-separated derivations generate dedicated fields for the chaotic initial state, modified Chen-system coefficients, fractional order, nonlinear-feedback parameters, adaptive trajectory selectors, three DNA base-rule selectors, and independently derived forward and backward SPARKLE256 duplex seeds. The derivation context is additionally bound to the image dimensions and image mode. For RGB images, a single RGB-specific context and shared key schedule are used rather than independently generated schedules for the three channels. This organization separates the confidential root secret from the derived algorithmic parameters and avoids reusing undifferentiated key material across functionally distinct encryption components [24].

The principal contributions of this study are summarized as follows:

1. Bounded key-conditioned nonlinear state feedback. To introduce additional secret-dependent trajectory variation without allowing the added nonlinear interaction to grow without control, the fourth equation of the fractional-order Chen system is augmented with the feedback term *κ*tanh(*βxz*). The parameters *κ* and *β* are derived from independent HKDF fields, and the magnitude of the additional term is bounded by *κ* for every system state. The mechanism is formulated and analyzed in Section 3.3. Across the admissible region, all 10,000 sampled parameter triplets produced finite and bounded trajectories, all 1000 Lyapunov-grid configurations satisfied *λ*_1_ > 0 and *λ*_2_ > 0, with *λ*_2,min_ = 0.0546, and all 35 representative configurations were accepted by the modified 0–1 chaos test, with a median statistic of *K* = 0.9971. These results show that the feedback parameters provide key-conditioned trajectory diversification while preserving the validated hyperchaotic operating regime; no universal increase in chaotic complexity is claimed.
2. Channel-aware adaptive permutation and SPARKLE256-based cross-channel diffusion. To reduce structural reuse associated with fixed traversal patterns and to avoid isolating RGB components into independently reset diffusion chains, the proposed architecture combines adaptive bit-plane permutation, position-dependent DNA transformation, and SPARKLE256-based two-pass ARX-duplex diffusion. Each of the eight Gray-coded bit planes is assigned one of eight key-schedule-selected scanning trajectories. The separately permuted RGB components are then serialized as *R*||*G*||*B*, after which the position-dependent DNA stage is followed by a forward SPARKLE256 duplex pass, deterministic inter-pass masking, and a backward duplex pass. The duplex state is not reinitialized at channel boundaries, so the complete RGB image is processed as one continuous 3*HW*-byte sequence and dependencies can propagate across color-component boundaries.
3. Session-coupled and domain-separated CSPRNG–HKDF key scheduling. The proposed key schedule begins with an independently generated 256-bit CSPRNG session secret and expands it through HMAC-SHA-512-based RFC 5869 HKDF operations with function-specific labels and image-context binding. The canonically encoded NOAA/SWPC temporal record is incorporated solely as public salt/context and contributes no secret entropy. Independent HKDF fields are assigned to the chaotic coefficients and initial state, fractional order, nonlinear-feedback parameters, trajectory selectors, DNA base-rule selectors, and the forward and backward SPARKLE256 duplex seeds. The resulting nominal confidential root-secret domain is 2^256^; the derived numerical and discrete parameters are deterministic schedule outputs and are not counted as independent secret entropy.
4. Multi-level dynamical and statistical validation. The proposed generator and operational ciphertext are evaluated using complementary dynamical, numerical, and statistical analyses rather than image-level metrics alone. Section 3.3.5 reports boundedness scanning, empirical lower-order-bound estimation, Lyapunov-spectrum analysis, estimator verification, the modified 0–1 chaos test, bifurcation analysis, and step-size and memory-length convergence tests. Section 4.1 and Section 4.2 further evaluate the fractional modified Chen keystream and operational ciphertext using NIST SP 800-22 and Dieharder, with AES-256-CTR included as a reference. In the NIST evaluation, the modified Chen keystream satisfied 187 of 188 subtest rows (99.47%), while the operational ciphertext satisfied 185 of 188 rows (98.40%); the AES-256-CTR reference satisfied 184 of 188 rows (97.87%). In the 111-row Dieharder evaluation, the keystream produced 109 passed, 2 weak, and 0 failed outcomes, whereas the ciphertext produced 106 passed, 5 weak, and 0 failed outcomes.
5. Stage-wise ablation of the encryption architecture. Section 4.10 evaluates the cumulative contribution of Gray-code transformation, bit-plane permutation, dynamic DNA mixing, the forward SPARKLE256 duplex pass, inter-pass masking, and the complete bidirectional diffusion configuration over all 11 RGB test images and the medical-image subset. The results distinguish the functional roles of the individual stages: bit-plane permutation primarily disrupts spatial organization; dynamic DNA mixing increases entropy and suppresses residual adjacent-pixel correlation; and the forward SPARKLE256 duplex introduces the principal differential propagation, which is preserved through inter-pass masking and the backward duplex stage. For the complete dataset, the full algorithm achieves a mean entropy of 7.9972, horizontal, vertical, and diagonal correlations of 0.0032, 0.0002, and −0.0008, respectively, and NPCR and UACI values of 99.6115% and 33.4546%.
6. Computational profiling and optimized implementation. Section 4.11 quantifies the computational cost of the proposed method using stage-wise measurements for a 256 × 256 RGB image and compares a pure-Python implementation with an optimized CPU implementation. Fractional-order Chen sequence generation remains the dominant computational component. By accelerating the chaotic recurrence with Numba and vectorizing bit-plane permutation and dynamic DNA processing with NumPy, the total encryption time is reduced from 100,722.174 ms to 1356.327 ms, corresponding to an overall speedup of approximately 74.26×. The optimized fractional-order Chen stage requires 935.052 ms, while the forward and backward SPARKLE256 duplex stages require 126.834 ms and 125.394 ms, respectively.

The remainder of this paper is organized as follows. Section 2 reviews the theoretical and algorithmic foundations of the proposed method, including the Gray-code transform, bit-plane decomposition, scanning trajectories, the four-dimensional hyperchaotic Chen system, fractional-order calculus and Grünwald–Letnikov discretization, multi-rule DNA encoding, and SPARKLE256-based two-pass ARX-duplex diffusion. Section 3 presents the proposed medical image encryption architecture, including the session-coupled CSPRNG–HKDF key schedule, key-schedule-driven adaptive bit-plane permutation, the key-controlled fractional-order modified Chen generator and validation of its admissible operating region, position-dependent dynamic DNA transformation, SPARKLE256-based two-pass ARX-duplex diffusion with inter-pass masking, and the complete encryption and decryption procedures. Section 4 reports the experimental evaluation and security analysis, including statistical randomness testing, histogram and correlation analyses, information entropy, differential attack resistance, key-space analysis, robustness against cropping and noise, known-plaintext and chosen-plaintext analyses, stage-wise ablation, and computational complexity and encryption speed measurements. Finally, Section 5 summarizes the principal findings, discusses the limitations of the proposed method, and outlines directions for future research.

## 2. Preliminaries

This section reviews the theoretical and algorithmic components underlying the proposed algorithm: the Gray code transform, bit-plane decomposition, scan patterns, the four-dimensional hyperchaotic Chen system, fractional-order calculus and Grünwald–Letnikov discretization, multi-rule DNA encoding, and SPARKLE256-based two-pass ARX-duplex diffusion. Their integration into the encryption algorithm is presented in Section 3.

### 2.1. Gray Code Transform

The binary reflected Gray code is an ordered binary coding scheme in which two consecutive codewords differ in exactly one bit; hence, their Hamming distance is one [25]. The binary reflected Gray-code transformation follows the standard formulation in [26]:

*G_n_*_−1_ = *B_n_*_−1_, *G_i_* = *B_i_*_+1_⊕*B_i_*,  *i* = 0, 1, …, *n* − 2, (1)

where *B_n_*_−1_ *B_n_*_−2_ …*B*_1_ *B*_0_ represents an *n*-bit binary number, *G_n_*_−1_ *G_n_*_−2_ …*G*_1_ *G*_0_ represents the corresponding Gray code, and ⊕ denotes the exclusive-OR operation.

### 2.2. Bit-Plane Decomposition

Binary bit-plane decomposition represents an 8-bit grayscale image or an individual channel of an RGB image as eight binary layers, each corresponding to a fixed significance level of the pixel or channel values [14]. Since each pixel or channel value ranges from 0 to 255, it can be represented by an 8-bit binary sequence.

Let *v* denote an *n*-bit unsigned integer represented by the binary sequence (*b_n_*_−1_*b_n_*_−2_ … *b*_1_*b*_0_). Its decimal value is expressed as

(2) $$v=\sum_{i=0}^{n-1}b_{i}2^{i}=b_{0}2^{0}+b_{1}2^{1}+\cdots +b_{n-1}2^{n-1}.$$

Here, *b_i_* ∈ {0, 1}, *i* ∈ {0, 1, …, *n* − 1}, and *b*_0_ denotes the least significant bit. For an 8-bit image value, *n* = 8 and *v* ∈ {0, 1, …, 255}.

Let *I* denote an 8-bit image channel of size *H* × *W*, and let *P_k_*, *k* ∈ {1, 2, …, 8}, denote its binary bit planes. At spatial position (*μ*, *ν*), the original channel value is reconstructed from the eight bit planes as:

(3) $$I\left(\mu ,\nu \right)=\sum_{k=1}^{8}2^{k-1}P_{k}\left(\mu ,\nu \right)=P_{1}\left(\mu ,\nu \right)+2P_{2}\left(\mu ,\nu \right)+\cdots +2^{7}P_{8}\left(\mu ,\nu \right).$$

Here,

*I*(*μ*, *ν*) ∈ {0, 1, …, 255},  *P_k_*(*μ*, *ν*) ∈ {0, 1},

where 0 ≤ *μ* < *H* and 0 ≤ *ν* < *W*. Under this one-based plane-indexing convention, the bit *b_k_*_−1_ of each channel value forms the plane *P_k_*. Consequently, *P*_1_ is the least significant bit plane, whereas *P*_8_ is the most significant bit plane. The decomposition is lossless because the weighted combination in Equation (3) reconstructs every original channel value exactly.

### 2.3. Scan Patterns

**Figure 1:**
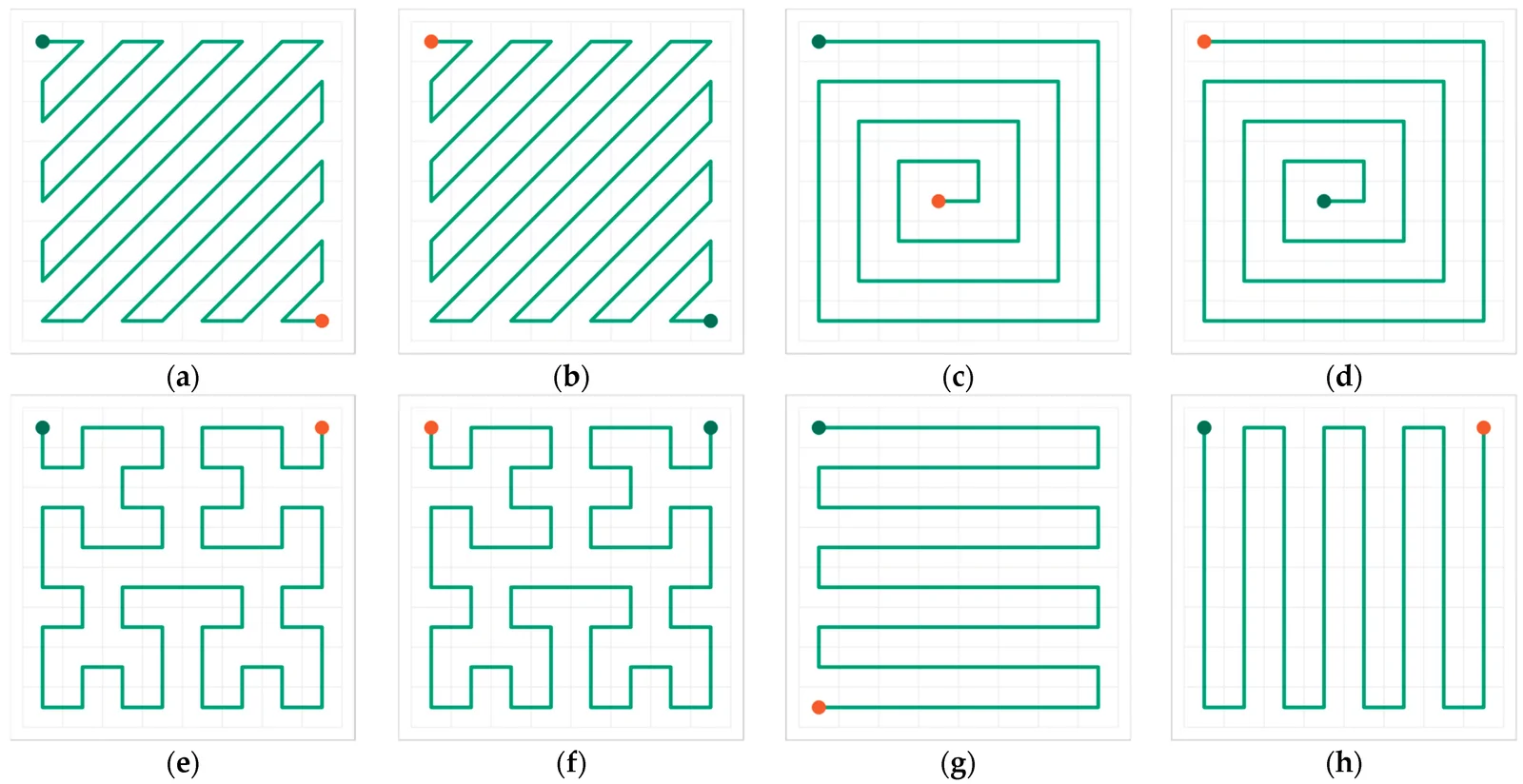
Visualization of the eight scan trajectories applied to an 8 × 8 grid. The zigzag, inward spiral, and Hilbert patterns are each traced in the forward and reverse traversal directions, while the snake pattern is shown in its row-wise and column-wise variants. The green marker denotes the starting position, and the red marker denotes the ending position of each traversal. (**a**) Zigzag scan (forward); (**b**) Zigzag scan (reversed); (**c**) Inward spiral scan (forward); (**d**) Inward spiral scan (reversed); (**e**) Hilbert curve scan (forward); (**f**) Hilbert curve scan (reversed); (**g**) Row snake scan; (**h**) Column snake scan.

A scan pattern defines the order in which the elements of a two-dimensional image or binary matrix are visited and converted into a one-dimensional sequence. For an image of size *H* × *W*, a valid scan path visits each spatial position exactly once and therefore establishes a bijective ordering over all *H* × *W* elements. The scan patterns considered in this study are zigzag, spiral, Hilbert, and snake traversals, as illustrated in Figure 1. The classical zigzag pattern traverses the matrix along successive diagonals with alternating directions [27].

In this study, the spiral traversal proceeds from the outer boundary toward the center, whereas the row-wise and column-wise snake traversals alternate the direction between successive rows or columns. The Hilbert traversal follows a space-filling curve that maps a two-dimensional image domain into a one-dimensional Hilbert order while preserving spatial locality [28]. Reverse zigzag, reverse spiral, and reverse Hilbert paths are obtained by reading the corresponding forward traversal in the opposite direction. Together with the row-wise and column-wise snake traversals, these variants yield eight distinct scan orders.

### 2.4. Four-Dimensional Hyperchaotic Chen System

The four-dimensional hyperchaotic Chen system is obtained by extending a modified three-dimensional Chen system with an additional dynamical feedback variable [29]. The original three-dimensional system is expressed as

(4) $$\dot{x}=a\left(y-x\right)\;\dot{y}=dx-\mathrm{xz}+\mathrm{cy}\;\dot{z}=\mathrm{xy}-\mathrm{bz}$$

where *x*, *y*, and *z* are the state variables, and *a*, *b*, *c*, and *d* are system parameters. By introducing an additional feedback variable *w*, the system is extended to four dimensions as follows:

(5) $$\dot{x}=a\left(y-x\right)+w\;\;\dot{y}=dx-\mathrm{xz}+\mathrm{cy}\;\;\dot{z}=\mathrm{xy}-\mathrm{bz}\;\;\dot{w}=\mathrm{yz}+\mathrm{rw}$$

where *r* denotes the feedback control parameter. The additional state variable increases the phase space dimension from three to four, allowing the system to exhibit hyperchaotic behavior. In general, a hyperchaotic system is characterized by the presence of at least two positive Lyapunov exponents.

Li et al. [29] denote the additional feedback state by *u*; in the present study, the same state variable is denoted by *w* for notational consistency with the subsequent fractional-order formulation. Apart from this notational relabeling, Equation (5) is identical to the four-dimensional system reported in [29].

For the parameter values *a* = 35, *b* = 3, *c* = 12, and *d* = 7, Li et al. reported that the system exhibits hyperchaotic dynamics within the interval 0.085 < *r* ≤ 0.798 [29].

**Figure 2:**
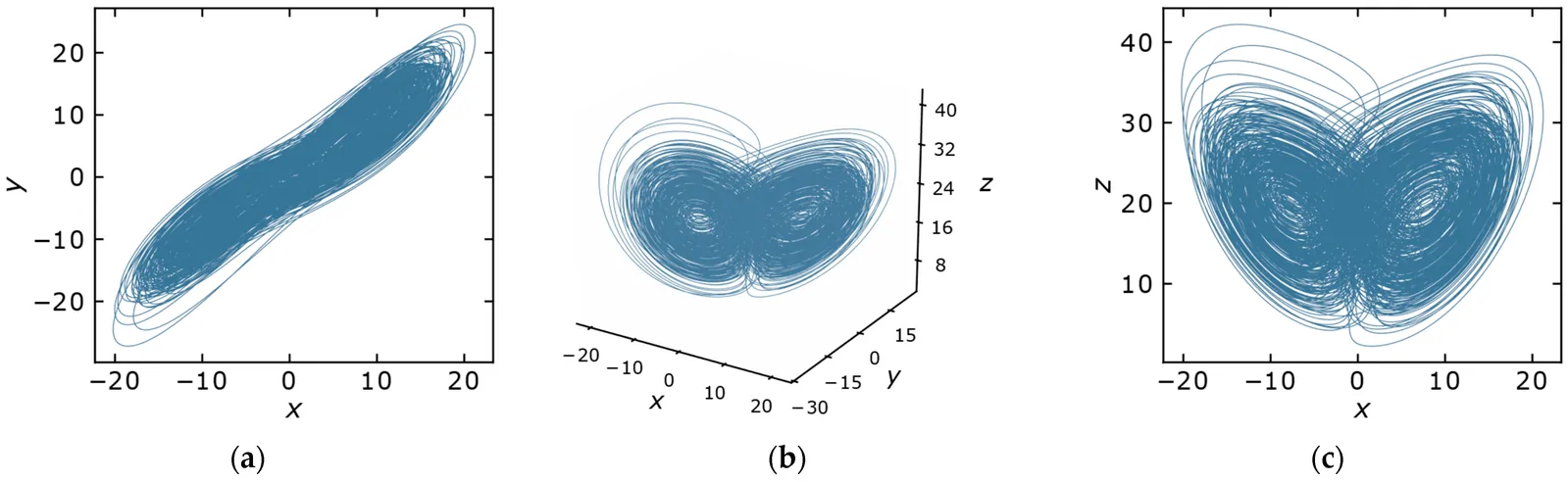
Attractor projections of the four-dimensional hyperchaotic Chen system defined by Equation (5) for *a* = 35, *b* = 3, *c* = 12, *d* = 7 and *r* = 0.5: (**a**) *x*–*y* plane; (**b**) x–z plane; (**c**) three-dimensional (*x*, *y*, *z*) projection.

Figure 2 illustrates the attractor of the four-dimensional system defined by Equation (5) for the parameter set reported in [29], with *r* = 0.5 selected from the interior of the hyperchaotic interval. The trajectory was obtained by fourth-order Runge–Kutta integration with a step size of 0.001 from the initial state (0.1, −0.2, 0.3, −0.1), and the first 10^5^ samples were discarded as transient. The *x*–*z* projection in Figure 2b exhibits the characteristic two-scroll structure inherited from the three-dimensional Chen system, whereas the three-dimensional projection in Figure 2c shows that the trajectory remains bounded and does not settle onto a periodic orbit. This integer-order configuration serves as the reference against which the fractional-order modified system introduced in Section 3.3 is compared.

### 2.5. Fractional-Order Calculus and Grünwald–Letnikov Discretization

Fractional calculus generalizes conventional integer-order differentiation to derivatives of non-integer order. Fractional-order systems exhibit nonlocal and memory-dependent behavior, since the current state is influenced by its previous states [30]. For a derivative order 0 < *α* < 1, the Grünwald–Letnikov fractional derivative of a function *x*(*t*) is defined as

(6) DtαGLxt=limh→01hα∑j=0t/h−1jαjxt−jh

where *h* denotes the discretization step and the generalized binomial coefficient is given by [31]

(7) $$\left(\begin{array}{c} \alpha \\ j \end{array}\right)=\frac{\Gamma \left(\alpha +1\right)}{\Gamma \left(j+1\right)\Gamma \left(\alpha -j+1\right)}$$

For a fractional-order dynamical system represented by

(8) $$D_{t}^{\alpha }X\left(t\right)=F\left(X\left(t\right)\right)$$

The Grünwald–Letnikov formulation produces a discrete recurrence in which the current state depends on a weighted sum of the preceding states. The corresponding Grünwald–Letnikov weights are defined as [31]

(9) $$\omega_{j}={\left(-1\right)}^{j}\;\left(\begin{array}{c} \alpha \\ j \end{array}\right)$$

and can be calculated recursively as [30]

(10) $$\omega_{0}=1\;\omega_{j}=\left(1-\frac{\alpha +1}{j}\right)\omega_{j-1},\;\;\;\;j\geq 1$$

As the simulation proceeds, the number of historical terms increases, reflecting the memory effect inherent in fractional-order dynamics [30].

To reduce the computational cost of retaining the complete state history, the short-memory principle approximates the full Grünwald–Letnikov sum by preserving only the most recent *L*_mem_ state samples. Here, *L*_mem_ denotes the finite memory length. The fractional order, discretization step, and memory length employed in the proposed algorithm are specified in Section 3.

### 2.6. Multi-Rule DNA Encoding

DNA encoding represents binary data using the four nucleotide symbols adenine (A), cytosine (C), guanine (G), and thymine (T), where each two-bit binary group is mapped to one DNA base. According to the Watson–Crick complementarity principle, adenine is complementary to thymine, while cytosine is complementary to guanine. Accordingly, the binary pairs 00 and 11, as well as 01 and 10, are treated as complementary pairs. Under these constraints, eight valid mappings can be defined between the binary set {00, 01, 10, 11} and the DNA alphabet {A, C, G, T} [32]. The rule ordering adopted in this study is presented in Table 1.

For an 8-bit value, the binary sequence is divided into four consecutive two-bit groups, and each group is encoded into one DNA base using the selected rule. Therefore, one byte is represented by a DNA sequence of length four. DNA decoding applies the inverse mapping of the same rule to recover the original binary value. The DNA exclusive-OR operation employed to combine two encoded DNA bases is defined according to the operation rules presented in Table 2.

For two DNA sequences of equal length, the operation is applied independently to the bases at corresponding positions. Let

*S* = (*s*_1_, *s*_2_, …, *s*_𝓁_)

and

*T* = (*t*_1_, *t*_2_, …, *t*_𝓁_)

denote two DNA sequences. Their DNA-domain XOR is defined as

*S*⊕_DNA_*T* = (*s*_1_⊕_DNA_*t*_1_, *s*_2_⊕_DNA_*t*_2_, …, *s*_𝓁_⊕_DNA_*t*_𝓁_) (11)

The operation is commutative and self-inverse:

*A*⊕_DNA_*B* = *B*⊕_DNA_*A*,  (*A*⊕_DNA_*B*)⊕_DNA_*B* = *A*. (12)

These properties enable the same DNA operand to be used to reverse the transformation during decryption. The mechanism used to select and assign the encoding and decoding rules in the proposed algorithm is described in Section 3.4.

### 2.7. SPARKLE256-Based Two-Pass ARX-Duplex Diffusion

Diffusion is used to propagate local modifications throughout the serialized data sequence and to suppress residual statistical dependencies after the preceding transformation stages. In the proposed framework, diffusion is implemented as a two-pass duplex construction built around the SPARKLE256 permutation. SPARKLE256 has a 256-bit internal state and combines Alzette ARX-boxes with the SPARKLE linear mixing layer. The diffusion stage uses SPARKLE256 with 10 steps throughout. The 256-bit state is ordered as rate || capacity, with a 128-bit rate portion followed by a 128-bit capacity portion.

The permutation used by the duplex construction is denoted by

SPARKLE_256,10_:{0, 1}^256^ → {0, 1}^256^. (13)

The diffusion input is the already defined byte sequence *Z* = (*z*_1_, *z*_2_, …, *z_M_*). Let *n_b_* denote the number of duplex blocks. For *M* ≥ 17, *n_b_* = ⌈*M*/16⌉; for 2 ≤ *M* ≤ 16, the sequence is split into two non-empty partial blocks and *n_b_* = 2. The one-byte case reduces to a keyed XOR and is not relevant to the image dimensions evaluated in this study. The block index is *ξ* ∈ {1, …, *n_b_*}, and Blk*_ξ_*(⋅) denotes the ξ-th block of a serialized byte sequence.

Each duplex direction is initialized from an independent 16-byte pass seed, Seed*_F_* and Seed*_B_*. Direction separation is provided by the 256-bit capacity-side domain-separation constants Tag*_F_* and Tag*_B_*. Their non-zero bits are XORed only into the capacity portion before the first permutation. The initial states are

(14) $${\mathrm{State}}_{0}^{\left(F\right)}={\mathrm{SPARKLE}}_{256,10}\left(\left(0^{128}∥{\mathrm{Seed}}_{F}\right)⊕{\mathrm{Tag}}_{F}\right)\;\;{\mathrm{State}}_{0}^{\left(B\right)}={\mathrm{SPARKLE}}_{256,10}\left(\left(0^{128}∥{\mathrm{Seed}}_{B}\right)⊕{\mathrm{Tag}}_{B}\right)$$

For one duplex step, Absorb(⋅) XORs the corresponding diffusion-keystream block into the 128-bit rate portion of the current state; Rate(⋅) returns that rate portion; Trunc(⋅) restricts the rate output to the current data-block length; and Feedback(⋅) XORs the produced block back into the rate. For a partial block, Feedback appends the byte 0 × 80 and then zero bytes until the 16-byte rate width is reached. The partial-block domain-separation constant Tag*_P_* is also XORed into the capacity portion before the permutation; for a full block this value is zero. The forward-pass block update is

(15) $${\mathrm{State}}_{\xi }^{\prime \left(F\right)}={\mathrm{SPARKLE}}_{256,10}\left(\mathrm{Absorb}\left({\mathrm{State}}_{\xi -1}^{\left(F\right)},{\mathrm{Blk}}_{\xi }\left(Q_{\mathrm{DIF}}^{\left(F\right)}\right)\right)⊕{\mathrm{Tag}}_{P,\xi }\right)\;{\mathrm{Blk}}_{\xi }\left(U\right)={\mathrm{Blk}}_{\xi }\left(Z\right)⊕\mathrm{Trunc}\left(\mathrm{Rate}\left({\mathrm{State}}_{\xi }^{\prime \left(F\right)}\right),\mathrm{len}\left({\mathrm{Blk}}_{\xi }\left(Z\right)\right)\right)\;{\mathrm{State}}_{\xi }^{\left(F\right)}=\mathrm{Feedback}\left({\mathrm{State}}_{\xi }^{\prime \left(F\right)},{\mathrm{Blk}}_{\xi }\left(U\right)\right),\;\;\xi =1,\ldots ,n_{b}.$$

ForwardDuplex(⋅) denotes the repeated application of Equation (15) for *ξ* = 1, …, *n_b_*. The backward pass uses the same update rule with $Q_{\mathrm{DIF}}^{\left(B\right)}$ and Seed*_B_*, but traverses *ξ* = *n_b_*, *n_b_* − 1, …, 1. At every backward step, block ξ is paired with ${\mathrm{Blk}}_{\xi }\left(Q_{\mathrm{DIF}}^{\left(B\right)}\right)$; the diffusion-stream index is therefore the absolute block index, not the ordinal position within the reverse traversal. Because encryption feeds the produced output block back into the rate, the decryptor can reproduce the same state evolution from the received block and recover the corresponding input block by XORing the same truncated rate value.

Two plain, oppositely directed duplex sweeps do not by themselves remove the structural boundary condition associated with the block processed last in the forward direction. The construction therefore inserts a common 128-bit inter-pass mask between the two sweeps. Fold(U) denotes the byte-wise XOR of the first and last forward-pass blocks over their common valid-byte range. The overall diffusion mapping is

(16) U=ForwardDuplex Z,QDIFF,SeedFΛ=MaskSP(Fold(U),SeedF⊕SeedB,nb)Umask=MaskBlocks(U,Λ)C=BackwardDuplex Umask,QDIFB,SeedB

Here, *Λ* is the inter-pass mask. MaskBlocks(⋅) XORs Λ into every intermediate block, truncating it only for a partial block. Because the same mask is applied to the first and last intermediate blocks, it cancels when those blocks are folded together. This permits the mask to be recomputed after inversion of the backward duplex pass. The construction is length preserving and consumes one diffusion-keystream byte per input byte in each direction, for a total requirement of 2*M* bytes. The concrete seed derivation, mask-generation rule, and inverse sequence are specified in Section 3.5.

## 3. Proposed Method

**Figure 3:**
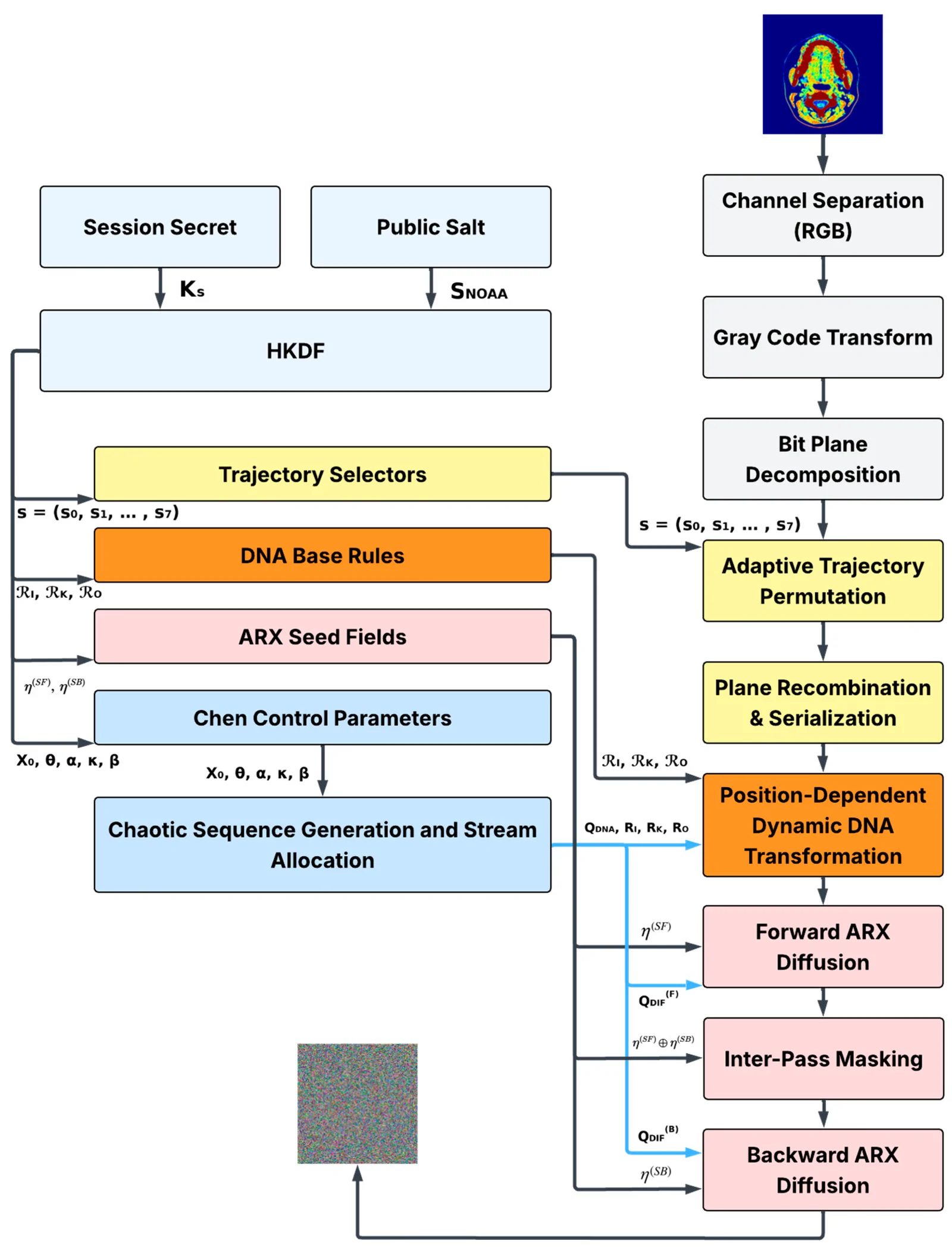
Proposed encryption algorithm.

This section presents the proposed medical image encryption algorithm, whose overall architecture is illustrated in Figure 3. The method integrates CSPRNG-based secret generation, HMAC-SHA-512-based HKDF key scheduling, Gray-code transformation, adaptive bit-plane permutation, a key-controlled modified four-dimensional fractional-order Chen system, position-dependent dynamic DNA encoding, and two-pass Add–Rotate–XOR (ARX) diffusion within a unified and fully reversible encryption framework. The image-transformation path and the key-schedule path are generated separately and subsequently combined through the permutation, DNA-domain substitution, and bidirectional diffusion stages.

Within this architecture, the transformation stages are assigned distinct functional roles rather than being introduced as independent operations. Adaptive bit-plane permutation primarily disrupts spatial organization, the position-dependent dynamic DNA stage performs value-domain transformation, and the bidirectional ARX diffusion stage propagates local changes throughout the serialized image data. These complementary roles are evaluated quantitatively through the stage-wise ablation analysis presented in Section 4.10.

For each encryption instance, a CSPRNG-generated 256-bit session secret is expanded through the domain-separated HKDF schedule described in Section 3.1, with the canonically encoded NOAA/SWPC record used only as public salt and context. The resulting schedule supplies function-specific parameters to the chaotic, permutation, DNA, and diffusion stages.

The schedule derivation is additionally bound to the image dimensions and image mode. A grayscale image is processed under a single *Y*-labeled context, whereas an RGB image is processed under one common RGB-labeled context. RGB images use a single RGB-specific key schedule shared across all three channels, while Gray-code transformation and adaptive bit-plane permutation are applied channel-wise before serialization.

Let the input image have spatial dimensions *H* × *W*. For a grayscale input, the image is treated as one channel. For an RGB input, the red, green, and blue components are first separated. Each channel is independently converted into its Gray-code representation and decomposed into eight binary bit planes. The eight planes of each channel are permuted using the common trajectory-selector vector and then recombined. The processed data are subsequently serialized in row-major order. For a grayscale image, the serialized sequence has length *M* = *HW*; for an RGB image, the three permuted channels are concatenated in the fixed RGB order, yielding *M* = 3*HW*. Thus, the DNA and diffusion stages operate on one continuous RGB byte sequence rather than on three independently encrypted channel sequences.

$$P=\mathrm{vec}\left(I_{\mathrm{perm}}^{\left(R\right)}\right)∥\mathrm{vec}\left(I_{\mathrm{perm}}^{\left(G\right)}\right)∥\mathrm{vec}\left(I_{\mathrm{perm}}^{\left(B\right)}\right),\;\;M=3\mathrm{HW}$$

In parallel with the image-transformation path, the schedule-derived initial state, Chen coefficients, fractional order, and nonlinear-feedback parameters are supplied to the fractional-order modified four-dimensional Chen system. The system is numerically evaluated using the Grünwald–Letnikov explicit recurrence with the short-memory principle. Following the warm-up stage, the four state components are jointly quantized to produce a byte-valued, chaotic sequence. For a processed sequence of length *M*, the full proposed configuration generates 6*M* chaotic bytes. These bytes are partitioned into six mutually non-overlapping segments: an *M*-byte DNA-value stream, three *M*-byte position-dependent DNA rule streams, and a 2*M*-byte diffusion stream. The diffusion segment is further divided into two *M*-byte subsequences assigned to the forward and backward ARX passes.

During the position-dependent dynamic DNA stage, the schedule provides three base-rule indices corresponding to image-byte encoding, chaotic-byte encoding, and output decoding. Three independently allocated chaotic rule streams modify these base indices at every sequence position. Therefore, each byte position may use a different ordered triplet of DNA rules. At position *i*, the permuted image byte and the corresponding DNA-value keystream byte are encoded using their position-dependent rules, combined through the DNA-domain exclusive-OR operation, and decoded using the position-dependent output rule. This stage changes byte values without altering the length or ordering of the serialized sequence.

The DNA-transformed sequence is subsequently processed by the two-pass ARX diffusion mechanism introduced in Section 2.7. The first pass traverses the complete serialized sequence in the forward direction using the forward diffusion keystream and the independently derived forward seed. The resulting intermediate sequence is then modified by the inter-pass mask and processed in the reverse direction using the independently allocated backward diffusion keystream and backward seed. Because the RGB components have already been concatenated, the duplex state is not reinitialized at the boundaries between the red, green, and blue segments. Consequently, dependencies originating in one color component can propagate across the remaining components through the continuous forward and backward diffusion passes.

After the backward ARX pass, the resulting ciphertext sequence is reshaped into the output image form. For a grayscale input, the *M*-byte sequence is reconstructed directly as an *H* × *W* cipher image. For an RGB input, the 3*HW*-byte ciphertext sequence is divided into three consecutive *HW*-byte segments, reshaped into the encrypted red, green, and blue components, and recombined to form the final RGB cipher image.

Decryption reproduces the same public schedule header, grayscale or RGB context, image dimensions, HKDF-derived schedule, fractional modified Chen trajectory, stream segmentation, DNA rule streams, diffusion keystreams, and the forward and backward diffusion seeds. The encryption stages are then inverted in reverse order. The backward ARX diffusion pass is inverted first, after which the inter-pass mask is recomputed and removed, followed by inversion of the forward ARX diffusion pass. The dynamic DNA transformation is subsequently reversed using the reproduced position-dependent rule triplets and DNA-value stream. Finally, each recovered channel undergoes inverse bit-plane permutation, bit-plane recombination, and inverse Gray-code transformation. The recovered channels are then combined to reconstruct the original image exactly. The following subsections describe each component of the proposed encryption algorithm in detail.

### 3.1. CSPRNG–HKDF Key Generation

Cryptographically secure pseudorandom number generators (CSPRNGs) are intended for security-sensitive applications in which generated values must be computationally unpredictable rather than merely exhibiting acceptable statistical randomness. In contrast to general-purpose pseudorandom generators, a CSPRNG is expected to prevent an adversary from feasibly predicting unrevealed outputs from an observed portion of the generated sequence or from partial knowledge of its internal state [33,34].

In the proposed key-generation mechanism, the confidential input keying material is a 256-bit session secret *K_s_* generated independently for each encryption instance by a CSPRNG:

*K_s_* ← CSPRNG(256),  *K_s_* ∈ {0, 1}^256^. (17)

Here, *K_s_* is the root secret from which the complete encryption configuration is derived. It is not mapped directly to the chaotic-system parameters, trajectory selectors, DNA rules, or diffusion variables. Instead, *K_s_* is supplied to the HKDF extraction stage as the input keying material. The same *K_s_* must be securely retained and supplied during decryption; it is never included in the public ciphertext packet.

**Figure 4:**
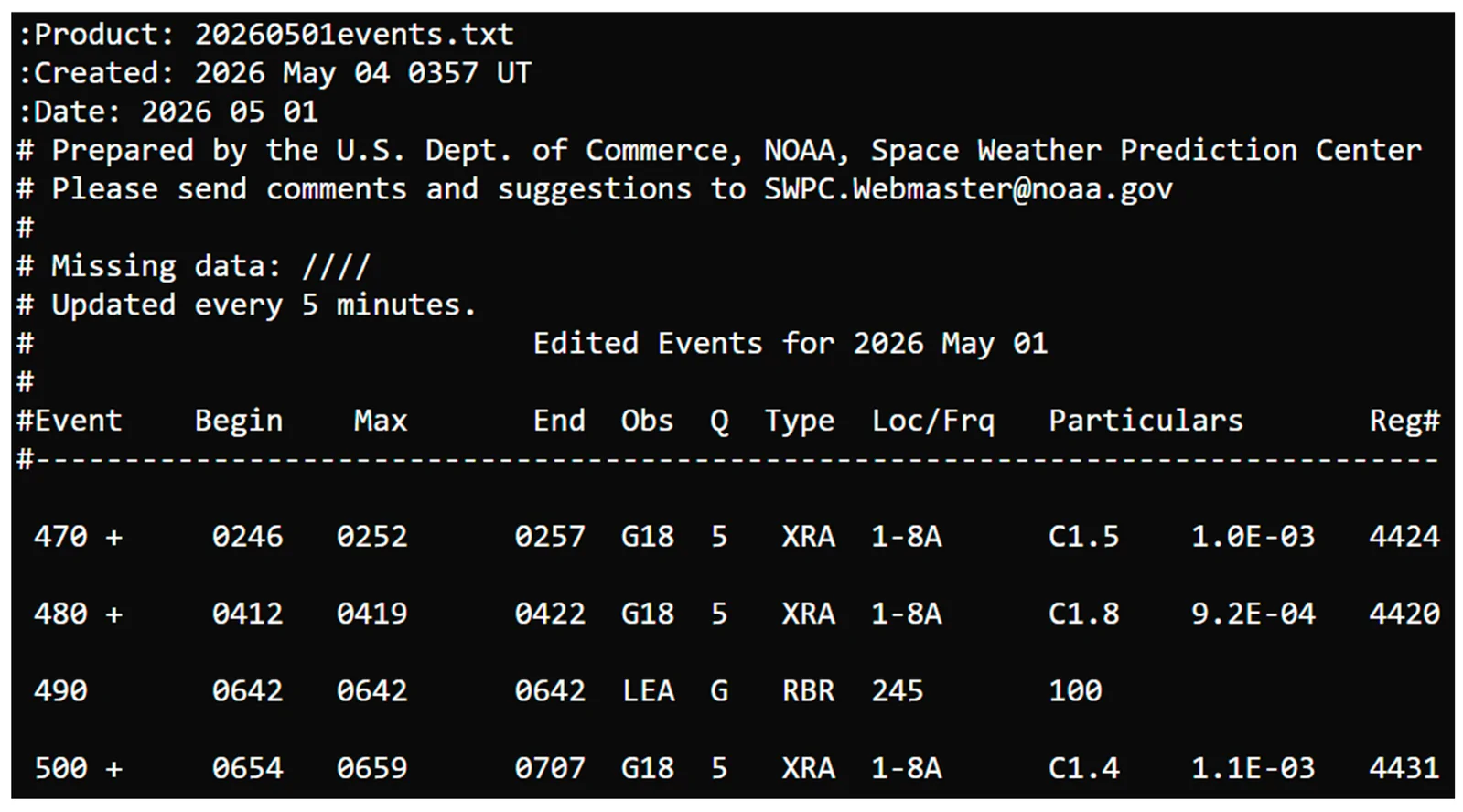
Screenshot excerpt from the NOAA/SWPC Daily Solar and Geophysical Event Report for 1 May 2026.

To bind the key-derivation process to a publicly reproducible temporal context, NOAA/SWPC solar-activity data are incorporated as non-secret salt information. NOAA/SWPC publishes event reports containing records of space-weather phenomena, including solar flares, geomagnetic storms, and radio blackouts [35,36,37]. In the proposed method, selected report fields are used only to provide an externally verifiable public context for schedule reproduction, auditability, and traceability; they are not treated as secret material and contribute no cryptographic entropy. An example excerpt from an NOAA/SWPC Solar and Geophysical Event Report is presented in Figure 4.

For each encryption instance, the public salt source is selected from the first recorded event in the NOAA/SWPC daily report corresponding to the day preceding encryption. If that report contains no event, the search proceeds to the immediately preceding report containing a recorded event. The selected Begin and Max values are placed in the public schedule header and canonically serialized to form *S*_NOAA_. This selection and serialization occur before HKDF processing. Because *S*_NOAA_ is public, confidentiality depends on *K_s_*.

The 256-bit session secret *K_s_* is supplied to HKDF as the input keying material, while *S*_NOAA_ is used as the public salt. Using HMAC-SHA-512, the extraction stage produces the 512-bit pseudorandom key PRK as

*PRK* = *HKDF* − *Extract_HMAC_*_− *SHA*512_(*S_NOAA_*,*K_s_*) (18)

In Equation (18), *S*_NOAA_ is the canonically encoded public salt, *K_s_* is the confidential input keying material, and PRK is the 512-bit pseudorandom key returned by HKDF-Extract. The use of public NOAA/SWPC data as salt does not weaken the role of *K_s_*; without the correct session secret, the same schedule cannot be reproduced.

The extracted PRK is then expanded into purpose-specific byte fields. Each field is generated by an independent HKDF-Expand invocation using a distinct domain-separation value info*_j_* and the required output length 𝓁*_j_*:

*K_j_* = HKDF − Expand_HMAC−SHA512_(PRK,info*_j_*,𝓁*_j_*) (19)

Here, *K_j_* is the byte string assigned to the *j*-th schedule field, info*_j_* is its public domain-separation context, and 𝓁*_j_* is its required length in bytes. The value info*_j_* combines the fixed algorithm identifier, the image dimensions *H* × *W*, the image-mode label *m* ∈ {*Y*,RGB}, and the semantic field label. A grayscale image uses a *Y*-labeled context, whereas an RGB image uses one common RGB-labeled context. Consequently, the complete RGB image is governed by one schedule; separate schedules are not derived for the red, green, and blue channels.

The field-specific outputs *K_j_* are assembled in a fixed canonical layout. The fields used by the full proposed configuration provide the four-dimensional chaotic initial state, the five modified Chen coefficients, the eight trajectory selectors, the dedicated forward and backward ARX-diffusion seed fields, the fractional order *α*, the nonlinear-feedback parameters *κ* and *β*, and the three DNA base-rule selectors. Since these fields are generated under distinct *info_j_* values, keying material assigned to one functional role is not reused as the direct input of another role.

During decryption, the ciphertext packet supplies the same public schedule header, while the legitimate recipient supplies the same confidential *K_s_*. Together with the recorded image dimensions and image mode, these values reproduce *S*_NOAA_, PRK, every info*_j_*, and the corresponding field outputs *K_j_*. This deterministic reconstruction yields the identical key schedule required by all subsequent inverse operations.

### 3.2. Key-Schedule-Driven Bit-Plane Permutation

The proposed permutation stage operates independently on the eight bit planes of each processed image channel. The 3-byte trajectory-selector field is derived once from the CSPRNG–HKDF key schedule defined in Section 3.1. For a grayscale image, the schedule is generated under the grayscale image context, whereas an RGB image uses one common RGB-specific schedule. Consequently, the red, green, and blue channels share the same trajectory-selector vector, although their bit-plane data are permuted independently.

Let *m* ∈ {*Y*,RGB} denote the image mode and define the corresponding channel-label set as 𝒥*_Y_* = {*Y*} and 𝒥_RGB_ = {*R*,*G*,*B*}. For each *χ* ∈ 𝒥*_m_*, let *I*^(*λ*)^ denote an 8-bit image channel of size *H* × *W*. Let *N* = *HW* denote the number of spatial positions in one channel. Each input channel is first converted into its Gray-code representation $I_{\mathrm{gray}}^{\left(\lambda \right)}\;$using the transformation defined in Section 2.1. The resulting, Gray-coded channel is then decomposed into eight binary planes $P_{k}^{\left(\lambda \right)}$, *k* ∈ {1, 2, …, 8}, according to the one-based plane-indexing convention defined in Section 2.2. 

Let the 3-byte selector field derived from the image-mode-specific key schedule be denoted by

(20) $$b_{\mathrm{sel}}=(\zeta_{0},\zeta_{1},\zeta_{2}),\;\;\zeta_{t}\in \{0,1,\ldots 255\}.\;S_{\mathrm{sel}}=2^{16}\zeta_{0}+2^{8}\zeta_{1}+\zeta_{2}.\;s_{k}=\mathrm{mod}\left(\mathrm{floor}\left(\frac{S_{\mathrm{sel}}}{2^{3\left(7-k\right)}}\right),8\right),\;\;k=0,1,\ldots ,7.$$

Here, *b*_sel_ is the 3-byte selector field, *S*_sel_ is the corresponding 24-bit unsigned selector word, and *s_k_* ∈ {0,…,7} is the trajectory selector assigned to the bit plane $P_{k+1}^{\left(\lambda \right)}$. Each selector is extracted from a distinct 3-bit segment of *S*_sel_. The mapping between the selector values and the eight available scanning trajectories is presented in Table 3.

The complete trajectory-selector vector is defined as

s = (*s*_0_, *s*_1_, …, *s*_7_) ∈ {0, 1, …, 7}^8^. (21)

The vector is common to all channel labels *χ* ∈ 𝒥*_m_*. Thus, a grayscale image applies s to its single *Y* channel, whereas the *R*, *G*, and *B* channels of an RGB image share the same RGB-specific selector vector. Consequently, each image-mode-specific schedule admits 2^24^ possible bit-plane-to-trajectory assignment vectors. The same trajectory may be assigned to multiple bit planes because the eight selectors are extracted independently from non-overlapping 3-bit segments. For an RGB image, the common selector vector is applied separately to the corresponding bit planes of the red, green, and blue channels.

For a trajectory type *j*, let *T_j_* denote the ordered sequence of row-major source indices generated for an *H* × *W* binary plane:

*T_j_* = (*τ_j_*(0), *τ_j_*(1), …, *τ_j_*(*N* − 1)). {*τ_j_*(0), *τ_j_*(1), …, *τ_j_*(*N* − 1)} = {0, 1, …, *N* − 1},  *j* ∈ {0, 1, …, 7}. (22)

The second line of Equation (22) states that every valid trajectory contains each source index exactly once. Hence, *T_j_* defines a bijective ordering over all *N* = *HW* elements of a bit plane.

The forward zigzag trajectory visits the diagonals satisfying

*μ* + *ν* = *δ*,  *δ* = 0, 1, …, *H* + *W* − 2

while alternating the traversal direction on consecutive diagonals. The inward spiral trajectory successively visits the upper, right, lower, and left boundaries of the remaining rectangular region. In the row-wise and column-wise snake trajectories, the traversal direction alternates according to the parity of the current row or column, respectively. The reverse zigzag and reverse spiral trajectories are obtained by reversing the ordered index sequences of their corresponding forward trajectories.

A separate construction is required for the Hilbert trajectory because a conventional Hilbert curve is defined over a square grid whose side length is a power of two. For an arbitrary *H* × *W* image channel, the side length of the auxiliary Hilbert grid is selected as

(23) $$L_{H}=2^{\mathrm{ceil}\left(\log_{2}\left(\max\left(H,W\right)\right)\right)}.$$

Let

$$H_{L_{H}}=\left(\left(\mu_{0},\nu_{0}\right),\left(\mu_{1},\nu_{1}\right),\ldots ,\left(\mu_{L_{H}^{2}-1},\nu_{L_{H}^{2}-1}\right)\right)$$

denote the conventional Hilbert traversal over the auxiliary *L_H_* × *L_H_* grid. The trajectory used for the original image domain is obtained by preserving only the coordinates that fall inside the *H* × *W* channel boundaries:

(24) $$T_{H}=\left(\mu_{t}W+\nu_{t}|\left(\mu_{t},\nu_{t}\right)\in H_{L_{H}},\;0\leq \mu_{t}<H,\;0\leq \nu_{t}<W\right).$$

Coordinates outside the original channel dimensions are discarded without changing the relative order of the retained coordinates. Consequently, *T_H_* contains exactly *N* row-major indices and remains bijective for non-square and non-power-of-two image dimensions. The reverse Hilbert trajectory is obtained by reading *T_H_* in the opposite direction.

To apply the selected trajectory, the *k*-th bit plane of channel *χ* ∈ 𝒥*_m_* is first represented as a row-major binary sequence. The source sequence and its permuted counterpart are defined as

(25) $$p_{k}^{\left(\lambda \right)}\left(n\right)=P_{k}^{\left(\lambda \right)}\left(\mathrm{floor}\left(\frac{n}{W}\right),\mathrm{mod}\left(n,W\right)\right).\;\;{\tilde{p}}_{k}^{\left(\lambda \right)}\left(n\right)=p_{k}^{\left(\lambda \right)}\left(\tau_{s_{k-1}}\left(n\right)\right),\;\;k=1,2,\ldots ,8,\;n=0,1,\ldots ,N-1.$$

Here, $p_{k}^{\left(\lambda \right)\;}$is the row-major representation of the *k*-th bit plane of channel *λ*, whereas ${\tilde{p}}_{k}^{\left(\lambda \right)}\;$is the corresponding permuted sequence. The selector *s_k_*_−1_ is used because the bit planes follow one-based indexing, *k* ∈ {1,…,8}, while the selector vector follows zero-based indexing, *s*_0_, …, *s*_7_. Equation (25) reads the source elements in the order specified by the selected trajectory and writes them consecutively into the permuted sequence.

The permuted binary sequence is reshaped into a two-dimensional bit plane, and the eight permuted planes are recombined to obtain the permuted channel:

(26) $${\tilde{P}}_{k}^{\left(\lambda \right)}\left(\mu ,\nu \right)={\tilde{p}}_{k}^{\left(\lambda \right)}\left(\mathrm{\mu W}+\nu \right).\;\;I_{\mathrm{perm}}^{\left(\lambda \right)}\left(\mu ,\nu \right)=\sum_{k=1}^{8}2^{k-1}{\tilde{P}}_{k}^{\left(\lambda \right)}\left(\mu ,\nu \right).$$

Here, ${\tilde{P}}_{k}^{\left(\lambda \right)}\;$denotes the two-dimensional form of the permuted *k*-th bit plane, and $I_{\mathrm{perm}}^{\left(\lambda \right)}$ denotes the channel reconstructed from the eight permuted planes. Although permutation is performed independently within each bit plane, the reconstructed byte at a given spatial position may contain bits originating from different source positions.

After channel-wise plane recombination, the resulting data are serialized for the position-dependent DNA transformation and two-pass ARX diffusion stages. Let vec(⋅) denote row-major vectorization. For a grayscale image,

$$P=\mathrm{vec}\left(I_{\mathrm{perm}}^{\left(Y\right)}\right),\;\;M=\mathrm{HW}.$$

For an RGB image, the three permuted channels are concatenated in the fixed order red, green, and blue:

$$P=\mathrm{vec}\left(I_{\mathrm{perm}}^{\left(R\right)}\right)∥\mathrm{vec}\left(I_{\mathrm{perm}}^{\left(G\right)}\right)∥\mathrm{vec}\left(I_{\mathrm{perm}}^{\left(B\right)}\right),\;\;M=3\mathrm{HW}.$$

Here, || denotes sequence concatenation, *P* is the complete serialized byte sequence supplied to the subsequent encryption stages, and *M* is its total length. Thus, RGB channels are processed separately during Gray-code conversion and bit-plane permutation but form one continuous sequence before the dynamic DNA and ARX diffusion stages.

During decryption, the same image dimensions, image mode, public salt, confidential session secret, and HKDF context are used to reproduce the identical selector vector and trajectory sequences. After inverse ARX diffusion and inverse dynamic DNA transformation, the recovered serialized sequence is reshaped directly into one *H* × *W* permuted channel for a grayscale image. For an RGB image, the recovered sequence is divided into three consecutive segments of length *N* = *HW*, and the segments are reshaped into the permuted red, green, and blue channels.

For each reconstructed permuted channel, the inverse permutation restores every received element to its original source index:

(27) $$p_{k}^{\left(\lambda \right)}\left(\tau_{s_{k-1}}\left(n\right)\right)={\tilde{p}}_{k}^{\left(\lambda \right)}\left(n\right).\;\;I_{\mathrm{gray}}^{\left(\lambda \right)}\left(\mu ,\nu \right)=\sum_{k=1}^{8}2^{k-1}P_{k}^{\left(\lambda \right)}\left(\mu ,\nu \right).$$

Here, ${\hat{p}}_{k}^{\left(\lambda \right)}\;$and ${\hat{P}}_{k}^{\left(\lambda \right)}\;$denote the recovered row-major sequence and recovered two-dimensional bit plane, respectively. The reconstructed, Gray-coded channel Igray λ is subsequently converted back to its ordinary binary representation by applying the inverse Gray-code transformation.

### 3.3. Key-Controlled Fractional-Order Modified Chen System and Keystream Generation

The proposed cryptosystem employs a key-controlled fractional-order modified Chen system as the pseudorandom byte source for all subsequent encryption stages. The modification introduces two independently derived nonlinear-feedback controls. No claim is made that the additional term universally increases chaotic complexity; instead, the coefficient, initial-state, fractional-order and feedback ranges are restricted to the bounded operating region that is validated numerically in Section 3.3.5. All quantities are derived deterministically from the function-specific HKDF fields introduced in Section 3.1, so that no user-selected dynamical parameter is required.

#### 3.3.1. System Definition

Let *X*(*t*) = [*x*(*t*),*y*(*t*),*z*(*t*),*w*(*t*)]^T^ denote the state vector. The commensurate fractional-order modified Chen system is defined by

(28) $$D_{t}^{\alpha }x=a\left(y-x\right)+w$$

(29) $$D_{t}^{\alpha }y=\mathrm{dx}-\mathrm{xz}+\mathrm{cy}$$

(30) $$D_{t}^{\alpha }z=\mathrm{xy}-\mathrm{bz}$$

(31) $$D_{t}^{\alpha }w=\mathrm{yz}+\mathrm{rw}+\kappa \tanh\left(\mathrm{\beta xz}\right)$$

where $D_{t}^{\alpha }$ denotes the fractional derivative of order *α* with 0 < *α* < 1; *a*, *b*, *c*, *d* and *r* are the Chen-system coefficients; and *κ* and *β* determine the gain and the sensitivity of the bounded nonlinear-feedback term, respectively. Because |tanh(*βxz*)| ≤ 1, the magnitude of the additional term is bounded above by *κ* for every admissible state. Setting *κ* = 0 reduces Equation (31) to the fourth equation of the unmodified four-dimensional Chen system, so the proposed generator contains the classical system as a strict special case.

#### 3.3.2. Key-Derived Parameterization

The nominal Chen-coefficient vector is ordered as *a*, *b*, *c*, *d* and *r*, and is given by

(32) $$\overset{‾}{\theta }={\left[\bar{a},\bar{b},\bar{c},\bar{d},\bar{r}\right]}^{T}={\left[35,3,12,7,0.5\right]}^{T}$$

Let $\eta^{\left(\theta \right)}=\left(\eta_{0}^{\left(\theta \right)},\eta_{1}^{\left(\theta \right)},\ldots ,\eta_{9}^{\left(\theta \right)}\right)$ denote the 10-byte HKDF field assigned exclusively to the Chen coefficients. The field is divided into five non-overlapping two-byte big-endian unsigned words. For j ∈ {0, 1, 2, 3, 4}, the corresponding 16-bit unsigned word and normalized value are

(33) $$v_{j}^{\left(\theta \right)}=256\eta_{2j}^{\left(\theta \right)}+\eta_{2j+1}^{\left(\theta \right)},\;u_{j}^{\left(\theta \right)}=\frac{v_{j}^{\left(\theta \right)}}{65535}$$

and the derived coefficient follows from a bounded relative perturbation of its nominal value,

(34) $$\delta_{j}=0.04u_{j}^{\left(\theta \right)}-0.02,\;\;\theta_{j}={\bar{\theta }}_{j}\left(1+\delta_{j}\right),\;\;j=0,1,2,3,4$$

Accordingly, every nominal coefficient varies within ±2% of its nominal value. The four-dimensional initial state is derived from a separate 32-byte HKDF field η^(X)^, divided into four non-overlapping eight-byte big-endian words. Each word is interpreted as a 64-bit unsigned integer, normalized by 2^64^ − 1, and mapped affinely onto [−1, 1], so that x_0_, y_0_, z_0_, and w_0_ each lie in the range reported in Table 4. The fractional order is derived from a separate two-byte field η^(a)^:

(35) $$v_{\alpha }=256\eta_{0}^{\left(\alpha \right)}+\eta_{1}^{\left(\alpha \right)},\;\;u_{\alpha }=\frac{v_{\alpha }}{2^{16}},\;\;\alpha =0.94+0.06u_{\alpha }$$

The fractional order is normalized by 2^16^ rather than 2^16^ − 1 so that the admissible interval remains half-open and the integer-order limit *α* = 1 is excluded from the production configuration, consistent with Table 4. The remaining fields, whose endpoints carry no comparable special significance, are normalized by 2^16^ − 1 and 2^64^ − 1. The nonlinear-feedback gain and sensitivity are derived from two further independent two-byte fields:

*κ* = 0.05 + 0.45*u_κ_*,  *β* = 0.0005 + 0.0045*u_β_* (36)

Because the coefficient, state, fractional-order, gain and sensitivity fields are generated through distinct labeled HKDF-Expand operations, bytes assigned to one dynamical role are never reused as the direct input of another. The resulting admissible ranges are summarized in Table 4.

The reported *r*-range lies entirely within the interval 0.085 < *r* ≤ 0.798 for which hyperchaotic behavior of the unmodified four-dimensional Chen system was reported in [29].

#### 3.3.3. Fundamental Dynamical Properties

Three structural properties of the modified system are established analytically before the numerical validation is presented. They are stated here because each of them constrains, and in one case excludes, the analytical tools that are conventionally applied to fractional-order chaotic systems.

(a) Dissipativity. The divergence of the vector field defined by Equations (28)–(31) is

(37) $$\nabla \;\cdot \;F=\frac{\partial \dot{x}}{\partial x}+\frac{\partial \dot{y}}{\partial y}+\frac{\partial \dot{z}}{\partial z}+\frac{\partial \dot{w}}{\partial w}=-a+c-b+r$$

The nonlinear-feedback term does not depend on w, so its partial derivative with respect to w vanishes identically and the divergence is unaffected by κ and β. For the nominal coefficients, this yields ∇·F = −35 + 12 − 3 + 0.5 = −25.5 < 0, and a phase-space volume element contracts as 𝒱(*t*) = 𝒱(0)exp(−25.5*t*). The negative divergence establishes uniform phase-space volume contraction throughout the admissible coefficient region. It does not, by itself, constitute a global boundedness proof; trajectory boundedness is therefore evaluated numerically over the complete sampled operating region in Section 3.3.5. The divergence identity is also used as an independent reference for validating the Lyapunov estimator.

(b) Symmetry. The system is invariant under the transformation

𝒮:(*x*, *y*, *z*, *w*) ↦ (−*x*, −*y*, *z*, −*w*) (38)

**Figure 5:**
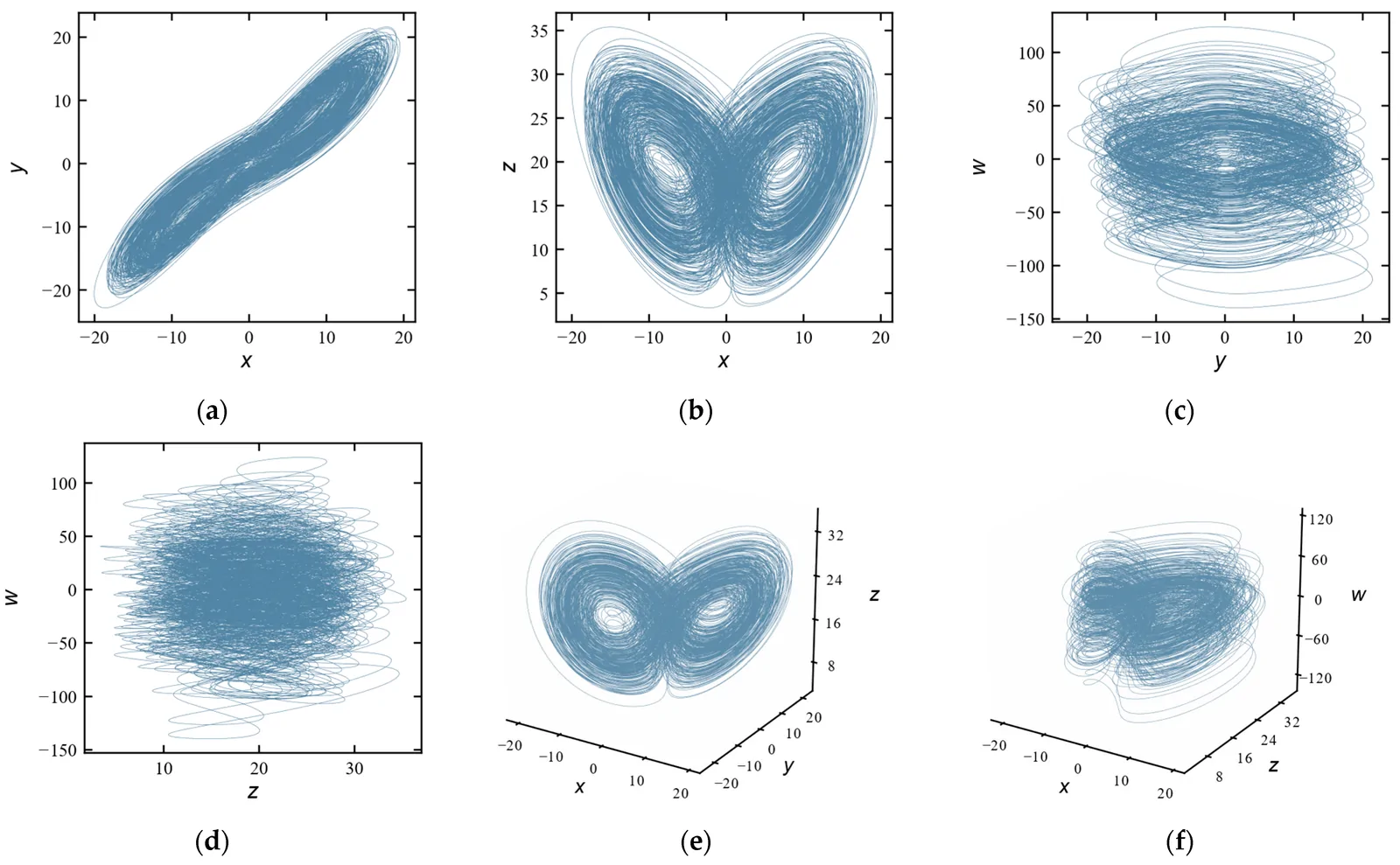
Attractor projections of the fractional-order modified Chen system at the mid-range key-derived configuration (α = 0.97, κ = 0.275, β = 0.00275): (**a**) x–y plane; (**b**) x–z plane; (**c**) y–w plane; (**d**) z–w plane; (**e**) three-dimensional (x, y, z) projection; (**f**) three-dimensional (x, z, w) projection. The two-lobe structure reflects the symmetry established in Equation (38).

Because tanh is an odd function, tanh(β(−x)z) = −tanh(βxz), and the fourth equation transforms consistently with the remaining three. The rotational symmetry of the unmodified four-dimensional Chen system is therefore preserved by the modification, which is reflected in the two-lobe structure of the attractor projections shown in Figure 5.

(c) Equilibria and local stability. Setting the right-hand sides of Equations (28)–(31) to zero yields a single equilibrium point for every admissible parameter set, namely the origin *O* = (0, 0, 0, 0). Since tanh(0) = 0, the feedback term contributes no additional equilibria. The Jacobian of the vector field is

(39) $$J\left(X\right)=\left[\begin{array}{cccc} -a & a & 0 & 1\\ d-z & c & -x & 0\\ y & x & -b & 0\\ \mathrm{\kappa \beta z}{\mathrm{sech}}^{2}\left(\mathrm{\beta xz}\right) & z & y+\mathrm{\kappa \beta x}{\mathrm{sech}}^{2}\left(\mathrm{\beta xz}\right) & r \end{array}\right]$$

Evaluated at the origin, the two feedback-dependent entries κβz·sech^2^(βxz) and κβx·sech^2^(βxz) both vanish. The Jacobian at O is therefore identical to that of the unmodified system, and the linear stability structure of the origin is independent of κ and β. The resulting spectrum is reported in Table 5.

All four eigenvalues are real. The equilibrium is consequently an unstable saddle with a two-dimensional unstable manifold, and not a saddle-focus. This has a direct methodological consequence: the necessary condition for chaos in commensurate fractional-order systems due to Tavazoei and Haeri presupposes the existence of a saddle-focus equilibrium with a complex conjugate eigenvalue pair, from which a minimum admissible order *α*_min_ = (2/π)·arctan(|Im(*λ*)|/Re(*λ*)) is obtained. Since no such equilibrium exists in the present system, that criterion is not applicable and no analytical lower bound on α can be derived from it. The admissible fractional order is therefore determined empirically in Section 3.3.5(b) rather than assumed. To the best of the authors’ knowledge, this property of the four-dimensional feedback-extended Chen system has not previously been stated explicitly in the image-encryption literature.

#### 3.3.4. Numerical Realization

The system is evaluated using the Grünwald–Letnikov explicit scheme introduced in Section 2.5, combined with the short-memory principle. For a retained memory length *L*, the GL weights are generated recursively as

(40) $$\omega_{0}=1,\;\;\omega_{j}=\left(1-\frac{\alpha +1}{j}\right)\omega_{j-1},\;\;j=1,2,\ldots ,L_{\mathrm{mem}}$$

and the state recurrence is

(41) $$X_{n+1}=h^{\alpha }F\left(X_{n}\right)-\sum_{j=1}^{\min\left(L_{\mathrm{mem}},n+1\right)}\omega_{j}X_{n-j+1}$$

In the production configuration *h* = 0.001, *L*_mem_ = 100, and 100 warm-up iterations are performed before byte generation begins. Two consequences of this discretization are relevant to the analysis that follows. First, at α = 1 the recursion in Equation (40) gives ω_1_ = −1 and *ω_j_* = 0 for all *j* ≥ 2, so the scheme reduces exactly to the forward Euler method and the map becomes genuinely four-dimensional; this property is exploited in Section 3.3.5(d). Second, for α < 1 the truncated recurrence is a deterministic map on the retained history window and therefore possesses an effective state dimension of 4*L*_mem_ = 400. The object characterized in this work is that map, since encryption and decryption both reproduce it exactly; identical arithmetic precision, memory length and numerical backend are required at both ends.

After the warm-up stage, each state is converted into one byte. Let *ψ_x_*_,*n*_, *ψ_y_*_,*n*_, *ψ_z_*_,*n*_ and *ψ_w_*_,*n*_ denote the four non-negative quantized state integers at iteration n:

*ψ_x_*_,*n*_ = ⌊10^6^|*x_n_*|⌋, *ψ_y_*_,*n*_ = ⌊10^6^|*y_n_*|⌋, *ψ_z_*_,*n*_ = ⌊10^6^|*z_n_*|⌋,  *ψ_w_*_, *n*_ = ⌊10^6^|*w_n_*|⌋ (42)

The output byte is obtained by combining all four quantized components through bitwise shifts and exclusive-OR:

*q_n_* = [*ψ_x_*_,*n*_⊕(*ψ_y_*_,*n*_≪1)⊕(*ψ_z_*_,*n*_≪2)⊕(*ψ_w_*_,*n*_≪3)]|mod|256 (43)

For a serialized image sequence of length M, the full configuration generates 6M bytes; their mutually non-overlapping allocation to the DNA-value stream, the three position-dependent DNA-rule streams and the two ARX-diffusion streams is specified in Section 3.4 and Section 3.5.

#### 3.3.5. Validation of the Admissible Operating Region

The admissible operating region defined by Table 4 was evaluated using the same short-memory GL recurrence, arithmetic precision and numerical backend employed by the production generator, rather than a simplified integer-order approximation or a different solver. The production generator begins byte extraction after 100 warm-up iterations; the asymptotic dynamical analyses below employ longer test-specific transients, stated separately for each experiment. Eight complementary analyses are reported. All computations were performed in double precision on the workstation described in Section 4, with fixed random seeds and recorded software versions.

(a) Boundedness of the sampled region. A three-dimensional 25 × 20 × 20 scan was performed over 0.94 ≤ α < 1, 0.05 ≤ κ ≤ 0.5 and 0.0005 ≤ β ≤ 0.005, yielding 10,000 evaluated parameter triplets. Every sampled trajectory remained finite and bounded under the numerical validity criterion used by the implementation, and the bit-balance and lag-one autocorrelation of the resulting byte streams satisfied the acceptance thresholds at all 10,000 triplets. Because the assessment is grid based, this result validates the sampled operating region; it is not presented as an analytical proof covering every point of the corresponding continuous parameter domain.

(b) Empirical determination of the lower fractional bound. Since the Tavazoei–Haeri criterion is inapplicable (Section 3.3.3), the lower limit of the admissible fractional order was located empirically by a three-stage bisection scan. At each sampled order, five parameter configurations spanning the corners and the center of the (κ, β) rectangle were integrated for 10^6^ observation steps after a transient of 10^5^ steps, under two independent integrator settings: the production setting (*h* = 10^−3^, *L*_mem_ = 100) and a matched-horizon setting (*h* = 5 × 10^−4^, *L*_mem_ = 200) that preserves the memory horizon *L*_mem_·*h* = 0.1. An order was classified as robust only when all five configurations passed under both settings. The results are summarized in Table 6.

The lowest contiguous robust order obtained from the ultra-refined stage is α* ≈ 0.9371. Over the retained band 0.940 ≤ α ≤ 0.999, all 600 long-horizon runs passed and the two integrator settings agreed at all 60 sampled orders. The production lower bound α = 0.94 therefore lies 0.0029 above the measured threshold. The complete agreement between the two settings, which differ in step size by a factor of two at a fixed memory horizon, provides direct evidence of step-size convergence for the classification.

**Figure 6:**
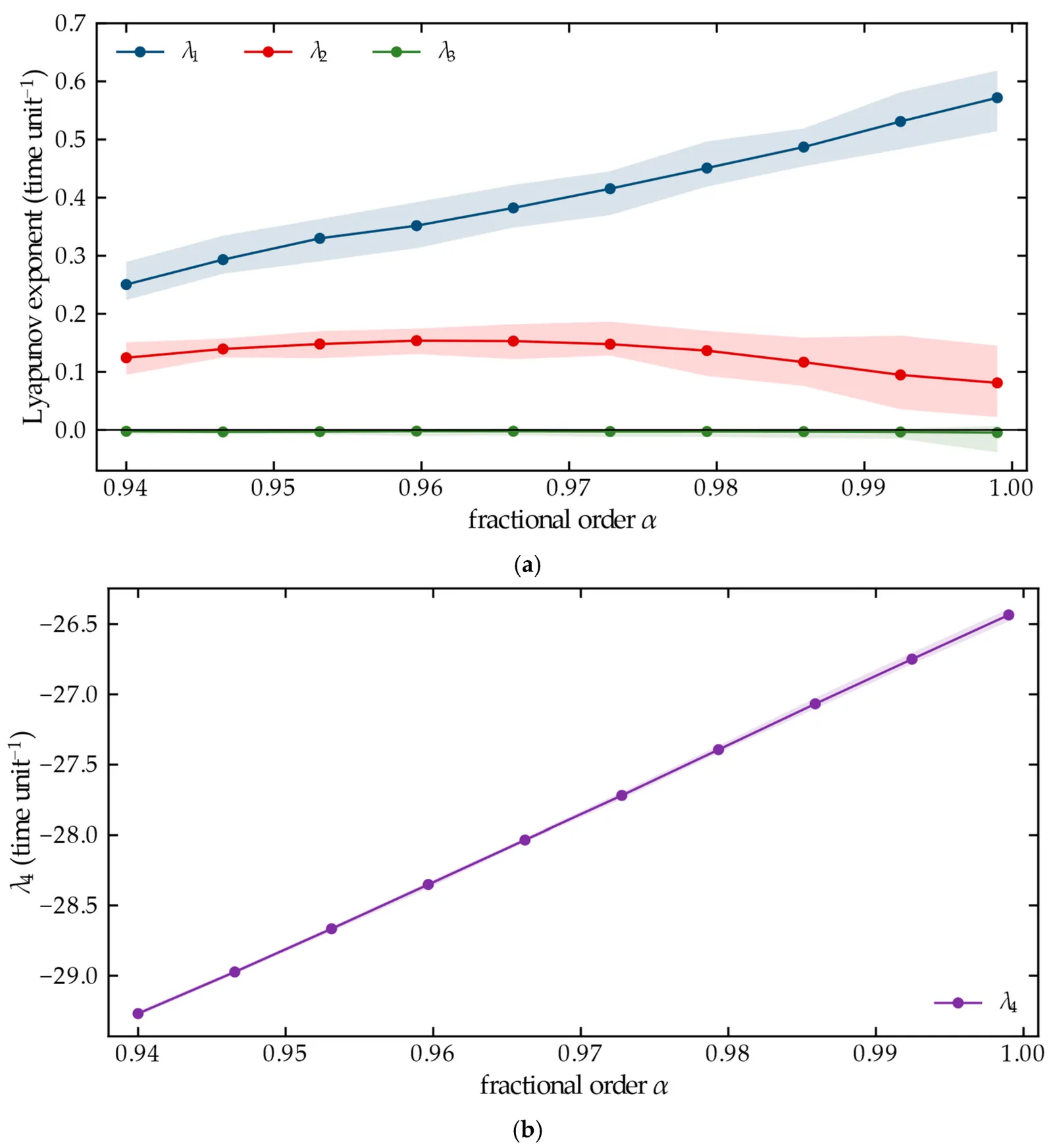
Lyapunov spectrum of the fractional-order modified Chen system over the admissible band, shown with a split vertical axis: (**a**) the three largest exponents λ_1_, λ_2_ and λ_3_; (**b**) the fourth exponent λ_4_. Markers denote the median over the 100 (κ, β) configurations sampled at each order and the shaded envelopes denote the corresponding minimum–maximum range.

(c) Lyapunov spectrum. Hyperchaos was assessed through the tangent dynamics of the exact short-memory GL companion map rather than through the classical 4 × 4 Jacobian of the integer-order differential system, since with *L*_mem_ = 100 the production recurrence has an effective state dimension of 4*L*_mem_ = 400. The four largest exponents were estimated on a 10 × 10 × 10 grid spanning the admissible region, using full-history Jacobian–vector products with periodic modified Gram–Schmidt reorthogonalization (transient 3 × 10^4^ steps, measurement 10^6^ steps, reorthogonalization every 20 steps). The finite-difference verification of the Jacobian–vector product agreed with the analytical form to a relative error of 7.8 × 10^−10^. All 1000 evaluated grid points satisfied the operational hyperchaos criterion λ_1_ > 0 and λ_2_ > 0. Median exponents per order are reported in Table 7 and the spectrum is plotted in Figure 6.

Two features of Table 7 are noteworthy. First, the largest exponent increases monotonically with the fractional order, whereas the second exponent attains a maximum near α ≈ 0.96 and decreases towards the integer-order limit. The strongest hyperchaotic character, measured by the second exponent, is therefore obtained inside the fractional band rather than at α → 1; this provides a dynamical, and not merely a key-space, motivation for operating the generator at fractional orders. Second, the third exponent remains close to zero at every sampled order (median −2.6 × 10^−3^ over the full grid; 968 of 1000 points satisfy |λ_3_| < 10^−2^). Recovery of the expected near-zero exponent along the flow direction is an internal consistency check on the estimator that does not depend on any external reference.

Because the smallest second exponents were obtained near the integer-order edge, where convergence is slowest, the 200 grid points with α ≥ 0.992 were re-measured over 10^7^ steps. This raised the minimum second exponent from 0.0222 to 0.0546 and confirmed that the lower values obtained at 10^6^ steps were under-converged; all 200 points remained hyperchaotic and the largest absolute third exponent was 2.8 × 10^−3^. Over the remaining band α < 0.992 the minimum second exponent at 10^6^ steps is 0.0759. The value λ_2_,_min_ = 0.0546 is therefore reported as a conservative lower bound over the whole admissible region; residual drift between the final two checkpoints remains larger near α → 1 (median 5.8%), but this bound leaves λ_2_ strictly positive at every sampled point, so the hyperchaos classification is unaffected by the remaining estimator uncertainty.

(d) Validation of the exponent estimator against the analytic divergence. As established in Section 3.3.4, the GL weights satisfy *ω_j_* = 0 for all *j* ≥ 2 when α = 1, so at that order the companion map is exactly four-dimensional and the four measured exponents constitute the complete spectrum. Their sum must then equal the analytic divergence of Equation (37). This provides an absolute reference against which the estimator can be tested. Table 8 reports the outcome at α = 1 with κ = 0, together with a step-size refinement study.

Table 8 reports relative deviations computed at full precision; the tabulated exponents are rounded to six decimals and do not reproduce them exactly. The deviation from the analytic divergence decreases linearly with the integration step, with successive ratios of 0.4960 and 0.4968 against the theoretical value of 0.5 for a first-order scheme. The residual is therefore attributable to forward-Euler truncation and not to the exponent estimator itself. Together with the recovery of λ_3_ ≈ 0 reported above, this establishes the reliability of the spectra in Table 7. It should be emphasized that the sum rule applies only in the limit α → 1: for α < 1 the companion map possesses 396 further exponents below λ_4_, so neither the sum of the four largest exponents nor a Kaplan–Yorke dimension computed from them carries the usual interpretation, and λ_4_ in Table 7 denotes the fourth-largest exponent of the companion map rather than the fourth exponent of an underlying four-dimensional flow.

**Figure 7:**
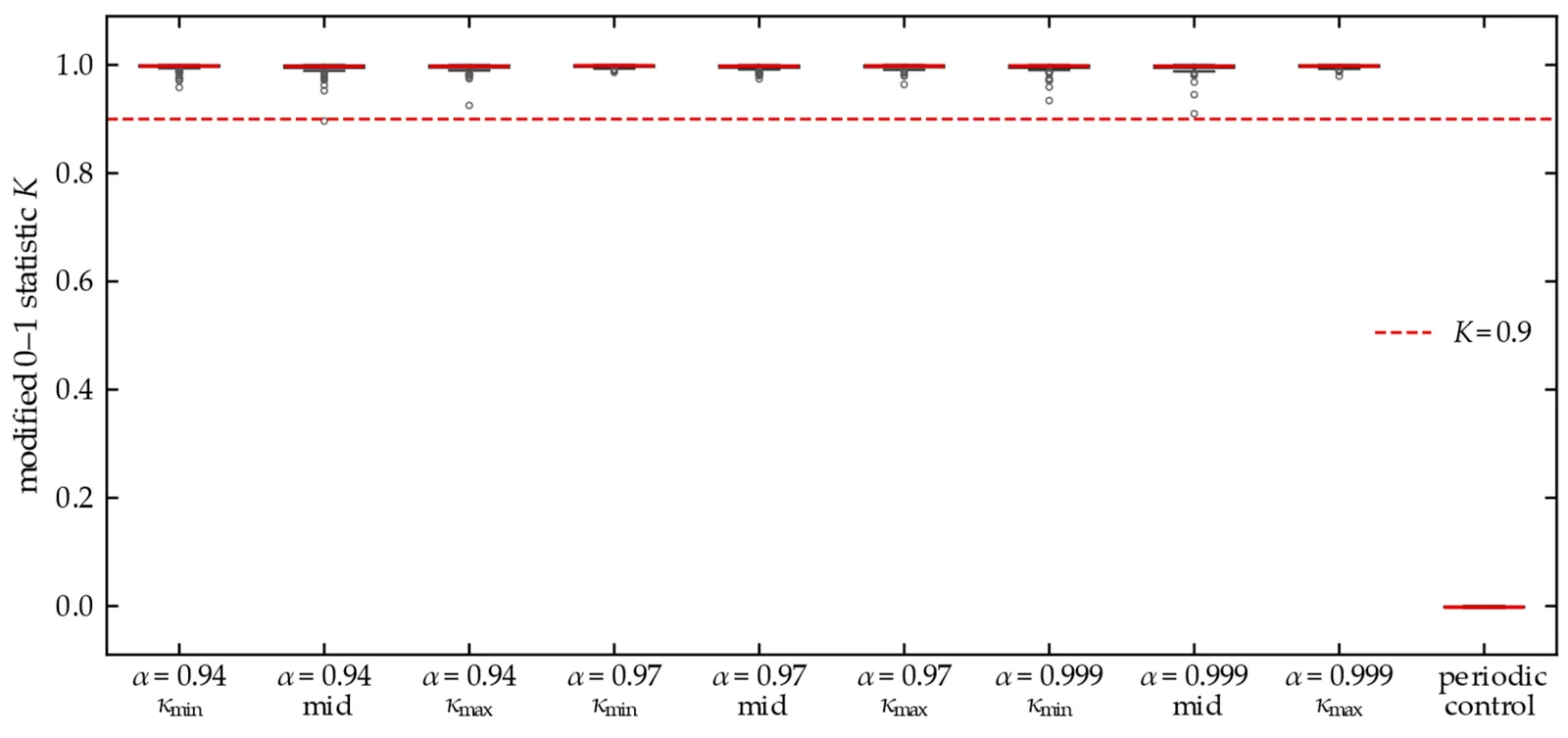
Distribution of the modified 0–1 test statistic K over 100 values of the test parameter c, shown for representative configurations at α = 0.940, 0.970 and 0.999 at the minimum, maximum and mid-range feedback gains, together with the periodic sinusoidal negative control. The dashed line marks the acceptance threshold K = 0.9.

(e) Modified 0–1 chaos test. The Lyapunov analysis was corroborated by the modified 0–1 test, which operates directly on the scalar observable and requires neither tangent dynamics nor reorthogonalization, and is therefore methodologically independent of the estimator validated above. Thirty-five representative configurations spanning the admissible region were integrated for 10^6^ observation steps after a transient of 10^5^ steps, sampled at a stride of 100 steps, and evaluated over 100 values of the test parameter *c*_test_. A periodic sinusoidal signal was included as a negative control. The results are given in Table 9 and Figure 7.

Acceptance was determined from the median *K* value calculated over the 100 values of the test parameter *c*_test_ for each case. The minimum individual-*c*_test_ statistic reported in Table 9 was included only as a diagnostic extreme and was not used as the acceptance criterion. An initial screening had applied the modified 0–1 test with a sampling stride of 10 steps, corresponding to an interval of 0.01 time units. This configuration proved to be strongly oversampled and returned *K* ≈ 0 uniformly, including at orders subsequently confirmed as chaotic by both the Lyapunov analysis and the longer-stride test. All 0–1 statistics reported in this work therefore correspond to the stride of 100 steps described above. The separation between the fractional cases and the periodic control exceeds 0.87 in the test statistic, and no fractional case approached the acceptance threshold.

**Figure 8:**
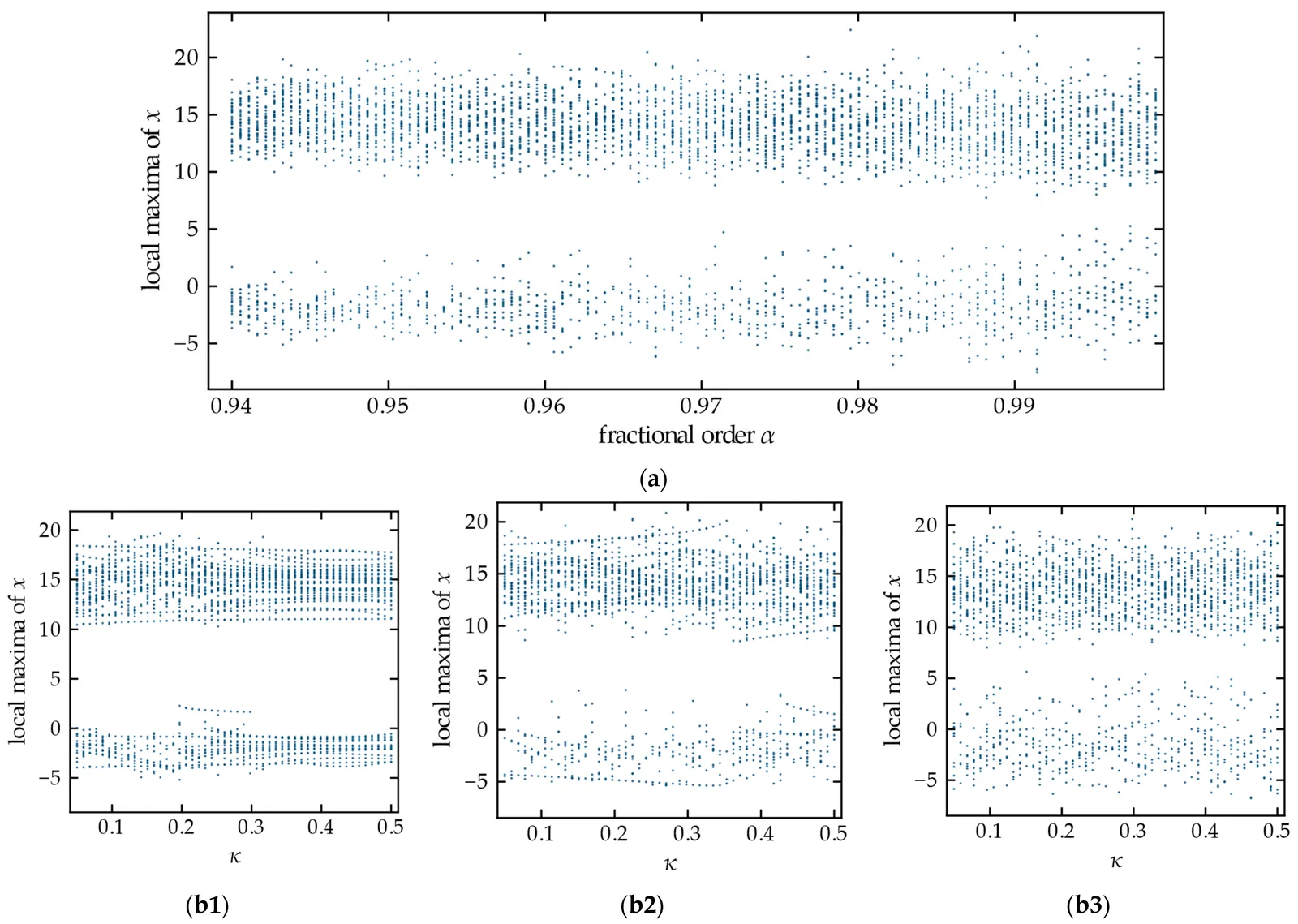

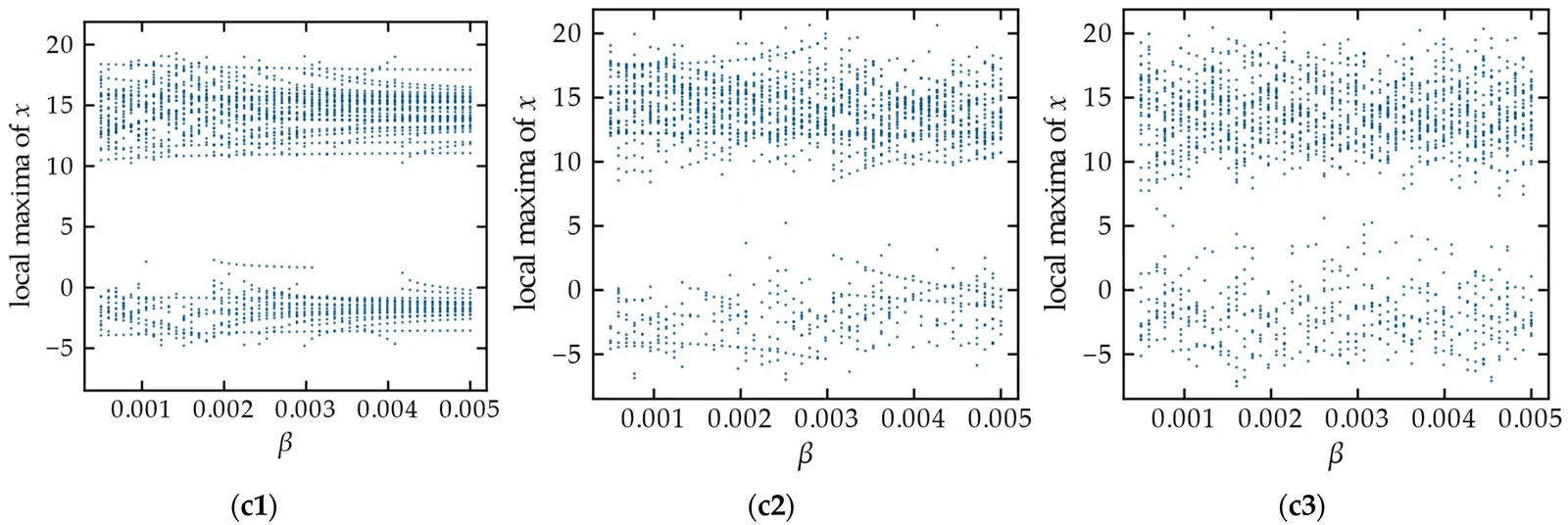
Bifurcation sections of the fractional-order modified Chen system: (**a**) variation with α over the admissible band; (**b1**) variation with κ at α = 0.94; (**b2**) variation with κ at α = 0.97; (**b3**) variation with κ at α = 0.99; (**c1**) variation with β at α = 0.94; (**c2**) variation with β at α = 0.97; (**c3**) variation with β at α = 0.99. The plotted values are the local maxima of *x* after the transient stage.

(f) Bifurcation structure. The bifurcation sections in Figure 8 provide a qualitative check that complements the quantitative indicators above. Across the sampled α, κ and β slices the local maxima of *x* remain persistently and densely distributed, with no broad transition to a fixed-point or low-period regime over the examined ranges.

(g) Numerical convergence in step size and memory length. Because the short-memory principle truncates the Grünwald–Letnikov history, the sensitivity of the generator to both h and *L*_mem_ was assessed by a full factorial campaign over *h* ∈ {2 × 10^−3^, 10^−3^, 5 × 10^−4^} and *L*_mem_ ∈ {50, 100, 200, 500}, evaluated at 25 configurations per cell. Table 10 reports the aggregate outcome.

The bit balance of the generated byte stream remains within 6 × 10^−4^ of 0.5 across the bounded cells. Lag-one autocorrelation remains at or below 10^−2^ in every cell except the shortest memory-horizon configuration (*L*_mem_·*h* = 0.025) and the two *L*_mem_ = 500 cells at *h* ≥ 10^−3^, all three of which lie outside the production regime. Two boundary effects are therefore visible. The cells with *L*_mem_ = 500 at *h* ≥ 10^−3^ exhibit divergence in a minority of configurations, which reflects the stability limit of the explicit GL scheme at large retained memory rather than a property of the underlying system; all such failures occurred at orders below the admissible band. Conversely, at the smallest memory horizon *L*_mem_·*h* = 0.025 the autocorrelation degrades. The production configuration *L*_mem_·*h* = 0.1 lies in the interior of the stable region, and the production cell (*h* = 10^−3^, *L*_mem_ = 100) and the matched-horizon cell (*h* = 5 × 10^−4^, *L*_mem_ = 200) return statistically indistinguishable results, consistent with the 60 of 60 agreement reported in Section 3.3.5(b).

(h) Role of the bounded feedback term. The influence of the feedback parameters on the spectrum was examined directly rather than asserted. Over the admissible region of Table 4, the leading exponents are governed almost entirely by the fractional order: the partial correlation of κ with λ_2_ after controlling for α is −0.015, the sign of the effect is consistent in only five of the ten sampled orders, and the median relative effect of 3.6% is of the same magnitude as the estimator noise. Within the production range, the feedback term provides key-dependent trajectory variation while the sampled configurations remain inside the numerically validated hyperchaotic region; no increase in chaotic complexity is claimed.

To establish whether this is a property of the term itself or of its admissible magnitude, an exploratory campaign widened the gain by two orders of magnitude in a separate branch that does not affect the reported cryptosystem. The outcome is summarized in Table 11. At the widened gain the relative magnitude of the feedback term rises from 0.073% to 7.3% of the dominant term of Equation (31), while the hyperbolic tangent remains entirely outside its saturation regime. Boundedness is retained at all 10,000 evaluated triplets and the median attractor size increases by only 4.9%. Under these conditions the dependence of the second exponent on κ becomes measurable and systematic, exhibiting a consistent sign across all ten sampled orders and a partial correlation of −0.295, while the leading exponent remains unaffected. This selective response indicates that the observed dependence is structural rather than numerical, because a spectrum-wide numerical bias would be expected to shift both exponents simultaneously.

The sign of the dependence is negative: increasing the gain reduces the second exponent. Beyond a gain–sensitivity product of approximately κβ ≈ 0.18 at the lower edge of the fractional band, the second exponent crosses zero and hyperchaos is lost, so that the admissible gain is bounded above by a dynamical and not merely a numerical constraint; recovering a fully hyperchaotic region at the widened gain additionally required raising the lower order bound to α = 0.95. These findings are reported for completeness and to delimit the parameter space; the cryptosystem evaluated in Section 4 retains the production ranges of Table 4, within which the feedback parameters act as key-controlled degrees of freedom that are numerically shown to preserve the hyperchaotic regime throughout the sampled production region. No claim of increased chaotic complexity is made.

Taken together, the analyses of this subsection define a numerically validated operating region for the key schedule. The region is bounded (10,000 triplets), delimited from below by a measured rather than assumed order threshold (α* ≈ 0.9371, with the production bound 0.0029 above it), hyperchaotic at every sampled point (1000 of 1000, with λ_2_,_min_ = 0.0546), corroborated by a methodologically independent chaos test (35 of 35 accepted against a separated negative control), stable under refinement of both the integration step and the retained memory, and served by an exponent estimator validated against an analytic invariant. Statistical randomness, key sensitivity, avalanche behavior and the cryptographic security of the generated keystream and ciphertext are evaluated separately in Section 4.

### 3.4. Position-Dependent Dynamic DNA Encoding

The eight valid binary-to-DNA mappings, byte-to-DNA conversion procedure, inverse decoding operation, and DNA-domain exclusive-OR operation were introduced in Section 2.6. This subsection therefore specifies how the proposed algorithm derives the three functional DNA rules and updates them independently at every byte position. The mechanism is applied to the complete serialized sequence produced in Section 3.2; consequently, an RGB image is processed as one continuous sequence after the channel-wise permutation and serialization stages.

Let *P* denote the serialized byte sequence supplied by the permutation stage, and let *Q*_DNA_ denote the non-overlapping chaotic subsequence allocated as the DNA-domain operand. Their common length is *M*, where *M* = *HW* for a grayscale image and *M* = 3*HW* for an RGB image:

*P* = (*p*_1_, *p*_2_, …, *p_M_*),  *Q*_DNA_ = (*γ*_1_, *γ*_2_, …, *γ_M_*) *M* = *HW* (gray),  *M* = 3*HW* (RGB),  *p_i_*, *γ_i_* ∈ {0, 1, …, 255} (44)

Here, *p_i_* is the *i*-th permuted image byte and *γ_i_* is the corresponding chaotic operand byte. Both values belong to the 8-bit integer alphabet {0, 1, …, 255}.

The key schedule contains three independent bytes assigned to the functional roles of image-byte encoding, chaotic-byte encoding, and output decoding. Let these schedule bytes be *η_I_*, *η_K_*, and *η_O_*, respectively. To avoid overloading the Chen coefficient *r*, the resulting DNA-rule indices are denoted by ℛ. The three base-rule indices are obtained by reduction modulo eight:

(45) $${\bar{R}}_{I}=\eta_{I}|\mathrm{mod}|8,\;\;{\bar{R}}_{K}=\eta_{K}|\mathrm{mod}|8,\;\;{\bar{R}}_{O}=\eta_{O}|\mathrm{mod}|8\;\;{\bar{R}}_{I},{\bar{R}}_{K},{\bar{R}}_{O}\in \{0,1,\ldots ,7\}$$

The base-rule indices provide a secret-dependent offset for each functional role, but they are not held constant over the sequence. As established in Section 3.3, the fractional modified Chen generator produces a single chaotic byte sequence *Q* = (*q*_0_, *q*_1_,…). For a processed sequence of length *M*, the first 4*M* bytes allocated to the DNA stage are partitioned into one value stream and three mutually non-overlapping rule streams:

*Q*_DNA_ = (*q*_0_, *q*_1_, …, *q_M_*_−1_) = (*γ*_1_, *γ*_2_, …, *γ_M_*) *R_I_* = (*q_M_*, *q_M_*_+1_, …, *q*_2*M*−1_) = (*ϕ_I_*(1), *ϕ_I_*(2), …, *ϕ_I_*(*M*)) *R_K_* = (*q*_2*M*_, *q*_2*M*+1_, …, *q*_3*M*−1_) = (*ϕ_K_*(1), *ϕ_K_*(2), …, *ϕ_K_*(*M*)) *R_O_* = (*q*_3*M*_, *q*_3*M*+1_, …, *q*_4*M*−1_) = (*ϕ_O_*(1), *ϕ_O_*(2), …, *ϕ_O_*(*M*)) (46)

The internally reindexed elements of *R_I_*, *R_K_*, and *R_O_* are denoted by *ϕ_I_*(*i*), *ϕ_K_*(*i*), and *ϕ_O_*(*i*), respectively. At position *i*, the actual rule index for each functional role is computed by adding the relevant rule-stream byte to its base-rule index and reducing the result modulo eight:

(47) $$R_{I}\left(i\right)=\left[{\bar{R}}_{I}+\phi_{I}\left(i\right)\right]|\mathrm{mod}|8,\;\;R_{K}\left(i\right)=\left[{\bar{R}}_{K}+\phi_{K}\left(i\right)\right]|\mathrm{mod}|8\;\;R_{O}\left(i\right)=\left[{\bar{R}}_{O}+\phi_{O}\left(i\right)\right]|\mathrm{mod}|8,\;\;i=1,2,\ldots ,M$$

Thus, each byte position is associated with the ordered rule triplet (ℛ*_I_*(*i*),ℛ*_K_*(*i*),ℛ*_O_*(*i*)). The same triplet may occur at more than one position, but its occurrence is governed by the three independently allocated rule streams rather than by a single image-wide DNA-rule configuration. The functional assignments are summarized in Table 12.

Let *E*_ℛ_(*v*) denote the four-symbol DNA representation of byte *v* under the rule indexed by ℛ, and let *D*_ℛ_(*S*) denote the inverse decoding of a four-symbol DNA sequence *S* under the same rule. At byte position *i*, the permuted image byte and chaotic operand byte are encoded under their independently selected position-dependent rules. The resulting DNA sequences are combined by DNA-domain exclusive-OR and decoded under the position-dependent output rule:

(48) $$A_{i}=E_{R_{I}\left(i\right)}\left(p_{i}\right),\;\;B_{i}=E_{R_{K}\left(i\right)}\left(\gamma_{i}\right)\;\;\Omega_{i}=A_{i}{⊕}_{\mathrm{DNA}}B_{i},\;\;z_{i}=D_{R_{O}\left(i\right)}\left(\Omega_{i}\right),\;\;i=1,2,\ldots ,M$$

Here, 𝒜*_i_*, ℬ*_i_*, and *Ω_i_* are DNA sequences of length four. The use of *Ω_i_* avoids reusing *G_i_*, which denotes a Gray-code bit in Section 2.1. The DNA-domain exclusive-OR is applied independently to the four corresponding symbol pairs, as defined in Section 2.6. The byte sequence produced by the DNA stage is

*Z* = (*z*_1_, *z*_2_, …, *z_M_*),  *z_i_* ∈ {0, 1, …, 255} (49)

Sequence *Z* is passed directly to the two-pass ARX diffusion stage described in Section 2.7 and Section 3.5. The DNA stage changes the byte values without changing the sequence length or the ordering produced by the preceding permutation and serialization stage.

During decryption, the same session secret, public salt, image dimensions, image mode, and HKDF context reproduce the identical base-rule indices, chaotic operand stream, and three rule streams. The position-dependent rule indices are recomputed using Equation (47). Each received DNA-stage byte is encoded under the output rule, combined with the corresponding encoded chaotic operand by the same self-inverse DNA-domain exclusive-OR operation, and decoded under the image rule:

(50) $${\hat{\Omega }}_{i}=E_{R_{O}\left(i\right)}\left(z_{i}\right),\;\;B_{i}=E_{R_{K}\left(i\right)}\left(\gamma_{i}\right)\;\;{\hat{A}}_{i}={\hat{\Omega }}_{i}{⊕}_{\mathrm{DNA}}B_{i},\;\;{\hat{p}}_{i}=D_{R_{I}\left(i\right)}\left({\hat{A}}_{i}\right),\;\;i=1,2,\ldots ,M$$

The recovered byte ${\hat{p}}_{i}$ is equal to *p_i_* because the DNA-domain exclusive-OR operation is self-inverse, as established in Equation (12). No DNA-rule matrix or position-dependent rule metadata is transmitted with the ciphertext: all required values are reproduced deterministically from the same secret-dependent schedule and chaotic trajectory.

The term position-dependent dynamic DNA therefore has a precise operational meaning in the proposed design. The key schedule determines three base-rule offsets, whereas the three independent rule streams update the image, chaotic, and output rule indices at every byte position. This construction avoids a globally fixed DNA-rule triplet, thereby preserving exact reversibility while achieving position-dependent diversity.

### 3.5. Key-Schedule Integration of the SPARKLE256-Based Two-Pass ARX-Duplex Diffusion

This subsection instantiates the construction introduced in Section 2.7 using the image-mode-specific key schedule and the chaotic diffusion stream. Let *Z* = (*z*_1_, *z*_2_, …, *z_M_*) denote the output of the position-dependent dynamic DNA stage. As defined previously, *M* = *HW* for a grayscale image and *M* = 3*HW* for an RGB image. For RGB input, the serialized order remains red, green, and blue, and the duplex state is not reset at channel boundaries. The complete serialized RGB sequence is therefore processed as one continuous diffusion input.

For a sequence of length M, the fractional-order modified Chen generator supplies exactly 2M diffusion bytes. The segment is divided into two mutually non-overlapping subsequences,

(51) $$\begin{array}{c} Q_{\mathrm{DIF}}=Q_{\mathrm{DIF}}^{\left(F\right)}∥Q_{\mathrm{DIF}}^{\left(B\right)},\\ Q_{\mathrm{DIF}}^{\left(F\right)}=\left(q_{1}^{\left(F\right)},q_{2}^{\left(F\right)},\ldots ,q_{M}^{\left(F\right)}\right),\\ Q_{\mathrm{DIF}}^{\left(B\right)}=\left(q_{1}^{\left(B\right)},q_{2}^{\left(B\right)},\ldots ,q_{M}^{\left(B\right)}\right). \end{array}$$

Thus, no diffusion byte assigned to the forward pass is reused by the backward pass.

The two 16-byte duplex seeds are derived by the field-specific HKDF-Expand convention of Equation (19). The forward and backward seed fields are denoted by *η*^(*SF*)^ and *η*^(*SB*)^, respectively, and use the context values info*_SF_* and info*_SB_*. Their derivation is

*η*^(*SF*)^ = HKDF − Expand_HMAC−SHA−512_ (PRK, info*_SF_*, 16) *η*^(*SB*)^ = HKDF − Expand_HMAC−SHA−512_ (PRK, info*_SB_*, 16) Seed*_F_* = *η*^(*SF*)^,   Seed*_B_* = *η*^(*SB*)^. (52)

After the forward duplex pass produces *U*, the 128-bit inter-pass mask is generated from Fold(*U*), the XOR of the two pass seeds, and the block count *n_b_*. Let Tag*_Λ_* denote the mask-domain separation constant. Pad_128_(*X*) denotes right zero-padding of the byte string X to exactly 16 bytes; this is distinct from the 0 × 80-followed-by-zeros feedback padding used for partial duplex blocks. The mask-generation operator is defined by

Mask_SP_(*a*, *b*, *n_b_*): = Rate (SPARKLE_256,10_ ((Pad_128_(*a*)||*b*)⊕Tag*_Λ_*(*n_b_*, len(*a*)))) *Λ* = Mask_SP_ (Fold(*U*), Seed*_F_*⊕Seed*_B_*, *n_b_*) *U*_mask_ = MaskBlocks(*U*, *Λ*). (53)

In Equation (53), Tag*_Λ_* encodes the valid folded-byte count len(*a*) and the block count *n_b_* in the designated capacity-side words before the SPARKLE256 permutation is applied. Since the same Λ is XORed into the first and last intermediate blocks, Fold(*U*_mask_) = Fold(*U*) over their common valid-byte range. The decryptor can therefore regenerate Λ without transmitting it.

Decryption first inverts the backward duplex pass, recomputes and removes the inter-pass mask, and then inverts the forward duplex pass:

(54) $$U_{\mathrm{mask}}={\mathrm{BackwardDuplex}}^{-1}\left(C,Q_{\mathrm{DIF}}^{\left(B\right)},{\mathrm{Seed}}_{B}\right)\;\Lambda ={\mathrm{Mask}}_{\mathrm{SP}}(\mathrm{Fold}(U_{\mathrm{mask}}),{\mathrm{Seed}}_{F}⊕{\mathrm{Seed}}_{B},n_{b})\;U=\mathrm{MaskBlocks}(U_{\mathrm{mask}},\Lambda )\;Z={\mathrm{ForwardDuplex}}^{-1}\left(U,Q_{\mathrm{DIF}}^{\left(F\right)},{\mathrm{Seed}}_{F}\right).$$

The quantities required by the SPARKLE256-based two-pass ARX-duplex diffusion are summarized in Table 13. The two non-overlapping diffusion keystreams are obtained from the fractional-order modified Chen sequence, whereas the forward and backward duplex states are initialized by independently derived 16-byte HKDF fields. The inter-pass value Λis not an additional secret field; it is deterministically generated from the forward-pass intermediate sequence, the two pass seeds, and the block count according to Equation (53). Accordingly, no diffusion-specific value other than the two pass seeds is introduced as independent keying material.

To examine propagation beyond the structural definition of the diffusion stage, byte-level dependency and bit-level avalanche analyses were performed. For RGB images, Equation (54) operates on the complete 3*HW*-byte serialized sequence without state reinitialization at channel boundaries. The results showed broad propagation across the evaluated output domain, bit-transition probabilities centered close to the ideal one-half level, and no persistently unaffected output-bit region under the tested perturbations. These findings provide empirical support for the propagation characteristics of the construction rather than a formal cryptographic security proof.

### 3.6. Encryption/Decryption Algorithm

The complete full-configuration encryption and decryption procedures are summarized in Algorithms 1 and 2. The pseudocode follows the notation and operation order established in Section 3.1, Section 3.2, Section 3.3, Section 3.4 and Section 3.5. Static DNA-rule selection, direct-XOR diffusion, integer-order integration, and single-trajectory permutation remain ablation configurations and are not included in the proposed full procedure.

For RGB images, Gray-code conversion and adaptive bit-plane permutation are applied channel-wise before the processed channels are serialized as *R*||*G*||*B*. The subsequent key-schedule-driven chaotic, DNA, and two-pass ARX diffusion stages operate on this merged sequence rather than within a per-channel loop.
**Algorithm 1.** Encryption Process**Input:** *I* of size *H* × *W* (grayscale) or *H* × *W* × 3 (RGB); confidential 256-bit session secret *K_s_*; public NOAA/SWPC schedule header ℋ_pub_. **Output:** Cipher packet 𝒫 containing the ciphertext image *I_C_* and public reconstruction metadata. 1.Validate the input image *I* and the confidential session secret *K_s_* ∈ {0, 1}^256^; determine *H*, *W*, and the image-mode label *m* ∈ {*Y*, RGB}. 2.Obtain or validate the public schedule header ℋ_pub_ and canonically encode its NOAA/SWPC Begin and Max values as *S*_NOAA_. 3.PRK ← HKDF − Extract_HMAC−SHA512_(*S*_NOAA_,*K_s_*) 4.𝒦*_m_* ← DeriveSchedule(PRK, *H*, *W*, *m*) using the field-specific HKDF-Expand contexts defined in Section 3.1. 5.Parse 𝒦_m_ to obtain X_0_, θ, α, κ, β, the common selector vector s, the 16-byte forward and backward seed fields η^(SF)^ and η^(SB)^, and the base DNA rules ℛ_I_, ℛ_K_, and ℛ_O_. 6.Seed*_F_* ← η^(SF)^ and Seed*_B_* ← η^(SB)^, as defined in Equation (52). 7.If *m* = *Y*, set 𝒥 ← {*Y*} and *I*^(*Y*)^ ← *I*; otherwise split *I* into *I*^(*R*)^, *I*^(*G*)^, and *I*^(*B*)^, and set 𝒥 ← {*R*,*G*,*B*}. 8.for each *χ* ∈ 𝒥 do 9.$I_{\mathrm{gray}}^{\left(\chi \right)}\leftarrow$ GrayEncode(*I*^(*χ*)^). 10.$P^{\left(\chi \right)}\leftarrow \;\mathrm{AdaptiveBitPlanePermute}(I_{\mathrm{gray}}^{\left(\chi \right)},s)$ according to Section 3.2. 11.end for 12.if *m* = *Y* then *P* ← vec(*P*^(*Y*)^) and *M* ← *HW*. 13.else *P* ← vec(*P*^(*R*)^)||vec(*P*^(*G*)^)||vec(*P*^(*B*)^) and *M* ← 3*HW*. 14.end if 15.Determine the duplex-block count *n_b_* from M according to Section 2.7. 16.*Q* = (*q*_0_, *q*_1_, …, *q*_6*M*−1_)← ChenGL(*X*_0_, θ, *α*, *κ*, *β*, 6*M*; *h* = 0.001, *L*_mem_ = 100, warm − up = 100). 17.*Q*_DNA_ ← (*q*_0_, *q*_1_, …, *q_M_*_−1_) = (*γ*_1_, *γ*_2_, …, *γ_M_*) 18.*R_I_* ← (*q_M_*, *q_M_*_+1_, …, *q*_2*M*−1_) 19.*R_K_* ← (*q*_2*M*_, *q*_2*M*+1_, …, *q*_3*M*−1_) 20.*R_O_* ← (*q*_3*M*_, *q*_3*M*+1_, …, *q*_4*M*−1_) 21.$Q_{\mathrm{DIF}}^{\left(F\right)}\leftarrow \left(q_{4M},q_{4M+1},\ldots ,q_{5M-1}\right)$ 22.$Q_{\mathrm{DIF}}^{\left(B\right)}\leftarrow \left(q_{5M},q_{5M+1},\ldots ,q_{6M-1}\right)$ 23.for *i* = 1 to *M* do 24.$R_{I}\left(i\right)\leftarrow \left[{\bar{R}}_{I}+\phi_{I}\left(i\right)\right]|\mathrm{mod}|8$$,\;R_{K}\left(i\right)\leftarrow \left[{\bar{R}}_{K}+\phi_{K}\left(i\right)\right]|\mathrm{mod}|8$$,\;\mathrm{and}\;R_{O}\left(i\right)\leftarrow \left[{\bar{R}}_{O}+\phi_{O}\left(i\right)\right]|\mathrm{mod}|8$. 25.$A_{i}\leftarrow E_{R_{I}\left(i\right)}\left(p_{i}\right)$$\;\mathrm{and}\;B_{i}\leftarrow E_{R_{K}\left(i\right)}\left(\gamma_{i}\right)$. 26.$\Omega_{i}\;\leftarrow \;{𝒜}_{i}{⊕}_{\mathrm{DNA}}B_{i}\;\mathrm{and}\;z_{i}\leftarrow D_{R_{O}\left(i\right)}\left(\Omega_{i}\right)$ 27.end for 28.*Z* ← (*z*_1_, *z*_2_, …, *z_M_*) 29.$U\;\leftarrow \;\mathrm{ForwardDuplex}(Z,Q_{\mathrm{DIF}}^{\left(F\right)},{\mathrm{Seed}}_{F})$ according to Equation (15). 30.*Λ* ← Mask_SP_(Fold(*U*), Seed*_F_* ⊕ Seed*_B_*, *n_b_*) and *U*_mask_ ← MaskBlocks(*U*,*Λ*), according to Equation (53). 31.$C\;\leftarrow \;\mathrm{BackwardDuplex}(U_{\mathrm{mask}},\;Q_{\mathrm{DIF}}^{\left(B\right)}$, Seed*_B_*) according to Equation (16). 32.if *m* = *Y* then *I_C_*← reshape(*C*, *H*, *W*). 33.else split *C* into three consecutive *HW*-byte segments, reshape them as $I_{C}^{\left(R\right)}$$,\;I_{C}^{\left(G\right)}$$,\;\mathrm{and}\;I_{C}^{\left(B\right)}$$,\;\mathrm{and}\;\mathrm{set}\;I_{C}\leftarrow \;\mathrm{MergeRGB}(I_{C}^{\left(R\right)},I_{C}^{\left(G\right)},I_{C}^{\left(B\right)})$ 34.end if 35.return the packet 𝒫 containing *I_C_*, ℋ_pub_, *H*, *W*, *m*, and the public configuration identifier; do not include *K_s_* or any DNA/diffusion stream.

**Algorithm 2.** Decryption Process**Input:** Cipher packet 𝒫 containing *I_C_* and ℋ_pub_; the same confidential 256-bit session secret *K_s_*. **Output:** Reconstructed image $\hat{I}$. 1.Read *I_C_*, ℋ_pub_, *H*, *W*, and the image-mode label *m* from 𝒫; require the same confidential session secret *K_s_*. 2.Canonically reconstruct *S*_NOAA_ from ℋ_pub_. 3.PRK ← HKDF-Extract_HMAC-SHA512_(*S*_NOAA_, *K_s_*). 4.𝒦*_m_* ← DeriveSchedule(PRK, *H*, *W*, *m*) using the same field-specific HKDF-Expand contexts. 5.$\mathrm{Parse}\;{𝒦}_{m}\;\mathrm{and}\;\mathrm{reproduce}\;X_{0},\;\theta ,\;\alpha ,\;\kappa ,\;\beta ,\;s,\;\eta^{\left(\mathrm{SF}\right)},\;\eta^{\left(\mathrm{SB}\right)},\;{\bar{R}}_{I}$$,\;{\bar{R}}_{K}$$,\;\mathrm{and}\;{\bar{R}}_{O}$. 6.Seed*_F_* ← η^(SF)^ and Seed*_B_*← η^(SB)^. 7.if *m* = *Y* then *C* ← vec(*I_C_*) and *M* ← *HW*. 8.$\mathrm{else}\;\mathrm{split}\;I_{C}\;\mathrm{into}\;I_{C}^{\left(R\right)}$$,\;I_{C}^{\left(G\right)}$$,\;\mathrm{and}\;I_{C}^{\left(B\right)}$$;\;\mathrm{set}\;C\leftarrow \mathrm{vec}\left(I_{C}^{\left(R\right)}\right)∥\mathrm{vec}\left(I_{C}^{\left(G\right)}\right)∥\mathrm{vec}\left(I_{C}^{\left(B\right)}\right)$ and *M* ← 3*HW*. 9.end if 10.Determine *n_b_* from M according to Section 2.7. 11.*Q* = (*q*_0_, *q*_1_, …, *q*_6*M*−1_)← ChenGL(*X*_0_, θ, *α*, *κ*, *β*, 6*M*; *h* = 0.001, *L*_mem_ = 100, warm-up = 100). 12.$\mathrm{Split}\;Q\;\mathrm{identically}\;\mathrm{into}\;Q_{\mathrm{DNA}},\;R_{I},\;R_{K},\;R_{O},\;Q_{\mathrm{DIF}}^{\left(F\right)}$$,\;\mathrm{and}\;Q_{\mathrm{DIF}}^{\left(B\right)}$ using Lines 17–22 of Algorithm 1. 13.*U*_mask_← BackwardDuplex^−1^(C, Q_DIF_^(B)^, Seed*_B_*). 14.*Λ* ← Mask_SP_(Fold(*U*_mask_), Seed*_F_* ⊕ Seed*_B_*, *n_b_*). 15.*U* ← MaskBlocks(*U*_mask_, *Λ*). 16.*Z* ← ForwardDuplex^−1^(*U*, Q_DIF_^(F)^, Seed*_F_*); Lines 13–16 implement the inverse sequence of Equation (54). 17.for *i* = 1 to *M* do 18.$R_{I}\left(i\right)\leftarrow \left[{\bar{R}}_{I}+\phi_{I}\left(i\right)\right]|\mathrm{mod}|8$, $R_{K}\left(i\right)\leftarrow \left[{\bar{R}}_{K}+\phi_{K}\left(i\right)\right]|\mathrm{mod}|8$, and $R_{O}\left(i\right)\leftarrow \left[{\bar{R}}_{O}+\phi_{O}\left(i\right)\right]|\mathrm{mod}|8$. 19.${\hat{\Omega }}_{i}\leftarrow E_{R_{O}\left(i\right)}\left(z_{i}\right)$$\;\mathrm{and}\;B_{i}\leftarrow E_{R_{K}\left(i\right)}\left(\gamma_{i}\right)$. 20.${\hat{A}}_{i}\leftarrow {\hat{\Omega }}_{i}{⊕}_{\mathrm{DNA}}B_{i}$$\;\mathrm{and}\;{\hat{p}}_{i}\leftarrow D_{R_{I}\left(i\right)}\left({\hat{A}}_{i}\right)$. 21.end for 22.$\hat{P}\leftarrow \left({\hat{p}}_{1},{\hat{p}}_{2},\ldots ,{\hat{p}}_{M}\right)$ 23.$\mathrm{if}\;m\;=\;Y\;\mathrm{then}\;\mathrm{reshape}\;\hat{P}$$\;\mathrm{as}\;{\hat{P}}^{\left(Y\right)}$ and set 𝒥 ← {*Y*}. 24.$\mathrm{else}\;\mathrm{divide}\;\hat{P}$into three consecutive *HW*-byte segments, reshape them as ${\hat{P}}^{\left(R\right)}$$,\;{\hat{P}}^{\left(G\right)}$$,\;\mathrm{and}\;{\hat{P}}^{\left(B\right)}$, and set 𝒥 ← {*R*, *G*, *B*} 25.end if 26.for each *χ* ∈ 𝒥 do 27.${\hat{I}}_{\mathrm{gray}}^{\left(\chi \right)}\leftarrow$$\;\mathrm{InverseAdaptiveBitPlanePermute}({\hat{P}}^{\left(\chi \right)},s)$. 28.${\hat{I}}^{\left(\chi \right)}\leftarrow$$\;\mathrm{GrayDecode}({\hat{I}}_{\mathrm{gray}}^{\left(\chi \right)})$. 29.end for 30.$\mathrm{if}\;m\;=\;Y\;\mathrm{then}\;\hat{I}\leftarrow {\hat{I}}^{\left(Y\right)}$$;\;\mathrm{otherwise}\;\hat{I}\leftarrow$$\;\mathrm{MergeRGB}({\hat{I}}^{\left(R\right)},{\hat{I}}^{\left(G\right)},{\hat{I}}^{\left(B\right)})$$;\;\mathrm{return}\;\hat{I}.$


## 4. Experimental Results and Security Analysis

This section evaluates the proposed encryption algorithm through comprehensive experiments on natural and medical RGB and grayscale images. The evaluation includes NIST SP 800-22 and Dieharder statistical randomness testing, histogram and chi-square analysis, correlation coefficient analysis, information entropy, differential attack analysis, key-space analysis, cropping and noise attacks, known-plaintext and chosen-plaintext attacks, an ablation study, and computational complexity and encryption speed analysis. All experiments were conducted in an Ubuntu 22.04 environment on a workstation equipped with an AMD Ryzen 9 7950X3D 16-core processor (Advanced Micro Devices, Inc., Santa Clara, CA, USA), 128 GB of RAM (Kingston Technology Company, Inc., Fountain Valley, CA, USA), and an NVIDIA GeForce RTX 4090 GPU (NVIDIA Corporation, Santa Clara, CA, USA) with 24 GB of memory. The experimental dataset consisted of standard benchmark test images and medical images selected from the Medical Imaging (CT-Xray) Colorization dataset.

### 4.1. NIST SP 800-22

NIST Special Publications comprise a series of reports produced by NIST’s Information Technology Laboratory to present research, guidelines, and outreach on information system security and related practices. These publications are intended to help federal agencies (and voluntary non-federal users) implement cost-effective security and privacy measures for information systems.

To determine whether the binary outputs of the proposed encryption algorithm contain statistically detectable regularities, the official NIST Statistical Test Suite (STS) 2.1.2 implementation of NIST SP 800-22 Rev. 1a was applied to three independently generated output groups: the fractional modified Chen keystream, the operational ciphertext, and an AES-256-CTR reference output. Each group comprised 100 independent binary sequences of 1,000,000 bits, corresponding to 100,000,000 evaluated bits per group.

For the NIST STS evaluation, each of the 100 input sequences was generated as an independent test instance. For the *i*-th sequence, an independent 256-bit session secret $K_{s}^{\left(i\right)}$ was generated by a CSPRNG together with a distinct test-generated NOAA/SWPC public salt $S_{\mathrm{NOAA}}^{\left(i\right)}$. Thus, the 100 sequences were not obtained by partitioning a single long output, but were generated from 100 separate $\left(K_{s}^{\left(i\right)},S_{\mathrm{NOAA}}^{\left(i\right)}\right)$ pairs. For the fractional modified Chen keystream group, each pair was processed through the HKDF-based key-scheduling procedure described in Section 3.1 to derive a session-specific chaotic initial state, fractional order, Chen-system coefficients, and nonlinear-feedback parameters, from which 125,000 bytes (10^6^ bits) of keystream were generated. For the operational-ciphertext group, the corresponding $\left(K_{s}^{\left(i\right)},S_{\mathrm{NOAA}}^{\left(i\right)}\right)$ pair was used to encrypt a standard RGB test image selected cyclically from Lena, Baboon, and Peppers, and the first 125,000 bytes of the flattened ciphertext were used as the NIST STS input. For the AES-256-CTR reference group, the same independently generated $K_{s}^{\left(i\right)}$ was used as the AES-256 key together with a distinct per-sequence IV, and 125,000 zero bytes were encrypted in CTR mode to obtain the corresponding 10^6^-bit reference output. The distinct $S_{\mathrm{NOAA}}^{\left(i\right)}$ values were used solely as public salt and were not credited with any additional secret entropy; consistent with the key-scheduling model defined in Section 3.1, the secret entropy of each test instance was provided by the independently generated 256-bit $K_{s}^{\left(i\right)}$.

The significance level was set to *α* = 0.01. All 188 subtest rows generated by the 15 statistical test families were retained. In particular, the separate rows produced by tests with multiple outputs were evaluated individually rather than being reduced to a single minimum value or combined at the test-family level.

For each subtest row, an individual sequence was counted as passing when its test *p*-value satisfied *p* ≥ *α*. Accordingly, the expected passing probability was defined as

$$\hat{p}=1-\alpha$$

The lower acceptance bound for the observed passing proportion was calculated using the three-standard-deviation interval recommended for NIST STS evaluations:

(55) $$p_{\mathrm{LB}}(m)=\hat{p}-3\sqrt{\frac{\hat{p}\left(1-\hat{p}\right)}{m}}$$

where *m* denotes the number of sequences eligible for the corresponding subtest row. For *m* = 100 and *α* = 0.01, the unrounded lower bound is *p*_LB_ = 0.960150. Consequently, a proportion of 96/100 lies slightly below the acceptance bound. For the Random Excursions and Random Excursions Variant tests, the bound was recalculated using the smaller number of eligible sequences produced by the corresponding state-visit condition.

The second evaluation criterion was the uniformity of the individual sequence *p*-values. The resulting uniformity *P_T_*-value was required to satisfy

*P_T_* ≥ 10^−4^

A subtest row was accepted only when both the passing-proportion criterion and the uniformity criterion were satisfied. Table 14 reports the number of accepted rows for each statistical test family and each evaluated output group.

As shown in Table 14, 187 of the 188 subtest rows (99.47%) satisfied both NIST acceptance criteria for the fractional modified Chen keystream, while 185 of 188 rows (98.40%) were accepted for the operational ciphertext. Under the identical protocol, the AES-256-CTR reference satisfied 184 of 188 rows (97.87%). The fractional modified Chen keystream produced one passing-proportion flag, the operational ciphertext produced three passing-proportion flags, and the AES-256-CTR reference produced four passing-proportion flags. No uniformity flags were observed for any of the three evaluated groups. These row-level differences provide a useful statistical reference context, but they do not imply cryptographic superiority or equivalence between the proposed method and AES-256-CTR.

For the fractional modified Chen keystream, the only flagged row occurred in Non-overlapping Template subtest 72, with a passing proportion of 96/100. For the operational ciphertext, the three flagged outcomes occurred in Non-overlapping Template subtests 34, 47, and 91, with passing proportions of 96/100, 95/100, and 96/100, respectively. For the AES-256-CTR reference, the four flagged outcomes occurred in Non-overlapping Template subtests 11, 95, 116, and 121, with passing proportions of 96/100, 96/100, 95/100, and 96/100, respectively. All uniformity *P_T_*-values for the three evaluated groups remained above 10^−4^. These isolated row-level outcomes were retained and reported without reclassification or family-level aggregation.

Overall, the NIST STS results do not reveal a broad or systematic statistical deficiency in either the fractional modified Chen keystream or the ciphertext under the evaluated sample configuration.

### 4.2. Dieharder Statistical Test Suite

The Dieharder statistical test suite extends the 18 original Diehard tests with additional statistical tests, including tests derived from the NIST suite, and is widely used for evaluating random and pseudorandom number generators. The suite examines generated sequences from complementary perspectives, including bit balance, run structure, matrix rank, permutation behavior, spatial distribution, serial dependence, and the distribution of the resulting *p*-values. Dieharder classifies each reported result as passed, weak, or failed according to the position of its *p*-value within the configured acceptance intervals. Isolated weak outcomes may occur even for statistically well-behaved generators, whereas persistent or reproducible failures provide stronger evidence of non-uniformity. Foreman et al. further emphasize that file-based Dieharder testing requires a sufficiently large quantity of input data because repeated reading of the same finite input can introduce artificial dependencies and produce misleading results. For smaller files, the numbers of test samples and *p*-value samples should therefore be adjusted to prevent input reuse [38].

In the present study, the Dieharder analysis was conducted on the same three output groups used in the NIST SP 800-22 evaluation: the fractional modified Chen keystream, the operational ciphertext, and an AES-256-CTR reference output. Each group comprised 12,000 independently generated test sessions, with each session contributing 125,000 bytes, resulting in 1,500,000,000 bytes of input data per group. The resulting inputs therefore represent concatenated outputs from independent test sessions rather than a single long message generated under one key.

A total of 79 test cases were considered, of which 77 satisfied the executable zero-rewind configuration. The Diehard Sums test was excluded because it is marked “Do Not Use” in the installed Dieharder test catalog, while one additional case could not be executed under the strict zero-rewind constraint. The tsamples values were fixed according to the zero-rewind calibration and applied unchanged across all three output groups, while all 77 configurations were executed with psamples = 100. Because several Dieharder tests produce multiple output rows for different tuple lengths or test components, these 77 configurations generated 111 result rows for each output group, as summarized in Table 15. All reported executions completed with zero input rewinds and the passed, weak, and failed classifications were retained exactly as produced by the installed Dieharder implementation; no retrospective reclassification of the native results was performed.

The complete 111-row results show broadly similar overall profiles for the three evaluated groups. The fractional modified Chen keystream produced 109 passed, 2 weak, and 0 failed rows, whereas the operational ciphertext produced 106 passed, 5 weak, and 0 failed rows. The AES-256-CTR reference produced 108 passed, 3 weak, and 0 failed rows. All three groups completed the analysis with zero input-rewind rows. Because every result row was generated using 100 *p*-value samples, all 111 rows satisfy the *N_p_* ≥ 10 criterion. Accordingly, the complete 111-row result set also constitutes the primary evaluation set.

The small number of weak outcomes observed across all three groups indicates that isolated extreme *p*-value distributions may occur even under the 100-*p*-value sampling configuration. These outcomes should therefore be interpreted in the context of the complete test set and the corresponding sampling conditions rather than in isolation. Nevertheless, the AES-256-CTR comparison is used only as a contextual statistical reference and does not imply cryptographic security equivalent to or superior to AES-256-CTR. Overall, no failed outcomes were observed for either the fractional modified Chen keystream or the operational ciphertext across the 111 evaluated rows, providing supporting evidence that no broad or persistent statistical anomaly was detected under the tested zero-rewind, 100-psample configuration.

### 4.3. Histogram and Chi-Square Analysis

In image encryption, histogram analysis evaluates the flatness of the pixel-value distribution in the encrypted image and its lack of correlation with the plain image, thereby assessing the confidentiality provided by the encryption process [39]. To quantitatively evaluate histogram uniformity, Pearson’s chi-square goodness-of-fit test was employed. For 8-bit images, using a significance level of α = 0.05 and 255 degrees of freedom, the critical chi-square value is 293.247. Accordingly, χ^2^ values below this threshold indicate that the encrypted-image histogram does not significantly deviate from a uniform distribution [40]. The test statistic measures the deviation between the observed frequency of each intensity level and the frequency expected under an ideal uniform distribution and is calculated as

(56) $$\chi^{2}=\sum_{k=0}^{L-1}\frac{{\left[O(k)-E(k)\right]}^{2}}{E(k)},$$

where *L* denotes the total number of possible intensity levels, *O*(*k*) represents the observed frequency of intensity level *k*, and *E*(*k*) is its expected frequency under the uniform-distribution assumption. For an image channel of size *M* × *N*, the expected frequency is obtained as

(57) $$E(k)=\frac{M\times N}{L},\forall k.$$

The uniformity assumption is not rejected when the calculated chi-square statistic satisfies

(58) $$\chi^{2}<\chi_{\alpha }^{2}(\mathrm{df}),$$

where $\chi_{\alpha }^{2}(\mathrm{df})$ is the critical chi-square value at significance level *α*, and *df* denotes the number of degrees of freedom. For an 8-bit image, *L* = 256 and therefore *df* = *L* − 1 = 255. At *α* = 0.05, the corresponding critical value is $\chi_{0.05}^{2}(255)=293.247$. Accordingly, a calculated value below 293.247 indicates that the encrypted-image histogram does not exhibit a statistically significant deviation from a uniform distribution.

**Figure 9:**
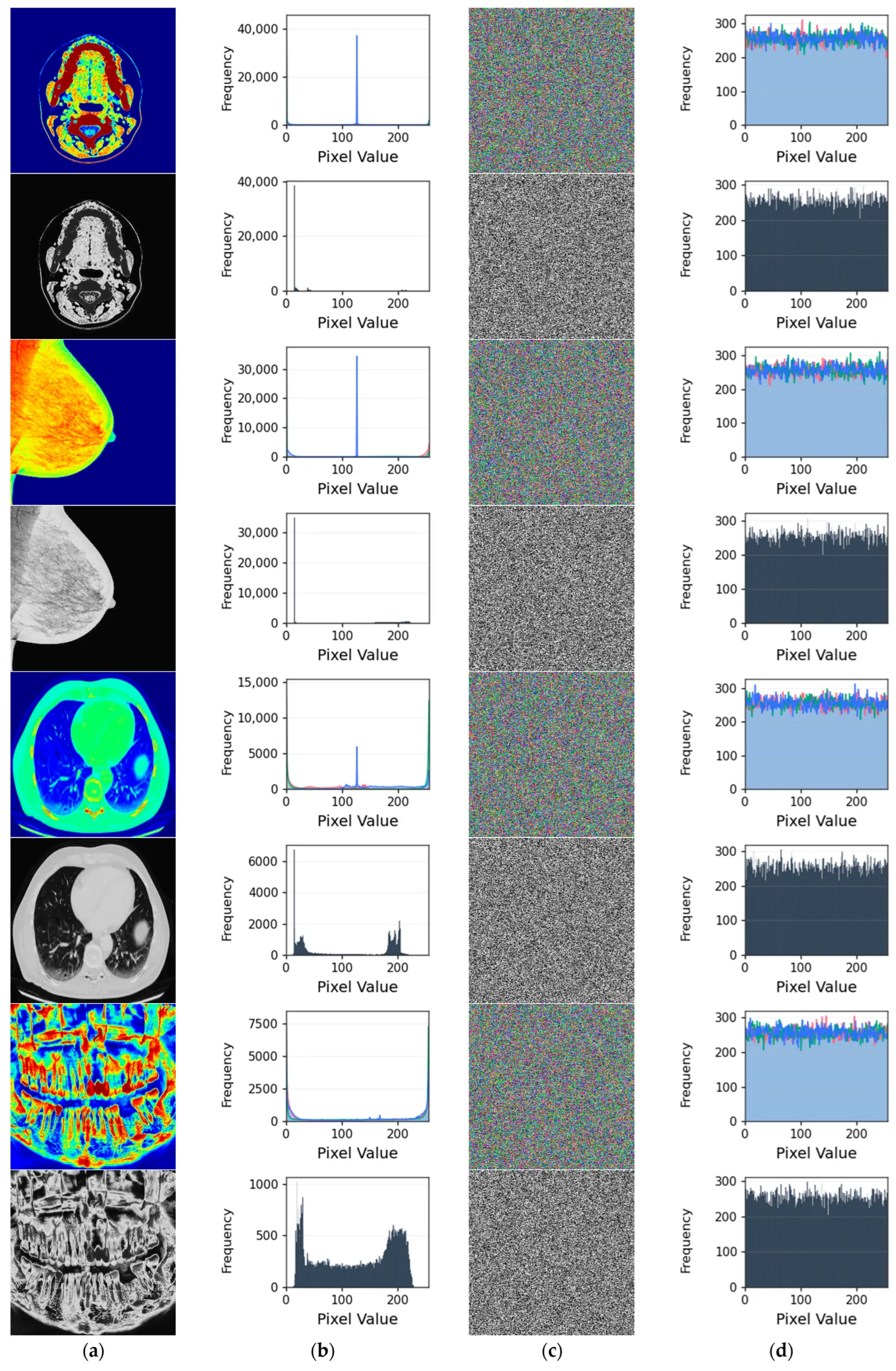
Histogram analysis of plain and encrypted test images: (**a**) plain images, (**b**) histograms of plain images, (**c**) encrypted images, (**d**) histograms of encrypted images.

Figure 9 presents the histogram distributions of representative plain and encrypted images in both RGB and grayscale formats. The plain-image histograms exhibit highly concentrated, irregular, and image-dependent distributions, reflecting the dominant intensity characteristics of the original images. In contrast, the histograms of the encrypted images are distributed more evenly across the entire intensity range from 0 to 255. The corresponding cipher images also display noise-like visual patterns, indicating that the proposed algorithm effectively suppresses the statistical characteristics and visual information of the plain images.

The quantitative chi-square results presented in Table 16 support the visual observations obtained from Figure 9. At the significance level *α* = 0.05 and *df* = 255, the critical chi-square value is 293.247. All calculated values remain below this threshold and therefore satisfy the histogram-uniformity criterion. For the encrypted RGB images, the chi-square statistics range from 238.1849 for Peppers to 272.9089 for Lena, whereas the grayscale-image results range from 227.0625 for Peppers to 272.9609 for Dental. The largest statistic among all tested images is obtained for the grayscale Dental image, with *χ*^2^ = 272.9609; however, this value also remains below the critical threshold. Consequently, the null hypothesis that the encrypted pixel values follow a uniform distribution cannot be rejected for any of the tested RGB or grayscale images. These results indicate that the encrypted-image histograms exhibit no statistically significant deviation from the expected uniform distribution under the chi-square test, consistent with the visually uniform histogram characteristics observed in Figure 9.

### 4.4. Correlation Coefficient Analysis

Correlation analysis is used to quantitatively measure the linear relationship between neighboring pixels in the horizontal, vertical, and diagonal directions and to evaluate the effectiveness of the confusion and diffusion mechanisms of an encryption algorithm. The correlation coefficient represents the linear dependence between adjacent value pairs at the bit or pixel level, and a secure encryption algorithm is expected to eliminate this dependence [41].

In a robust image encryption system, the absolute values of the correlation coefficients calculated in the horizontal, vertical, and diagonal directions are expected to be low and close to zero. This indicates that the high dependency between neighboring pixels in the plain image is effectively eliminated through the encryption process [41]. Correlation coefficients close to zero suggest that there is no significant linear relationship between the pixels of the encrypted image and support the robustness of the algorithm against statistical attacks [42]. In contrast, high correlation values indicate that the dependency between neighboring pixels has not been sufficiently removed and may suggest inadequacies in the confusion or diffusion stages.

To quantitatively evaluate the statistical dependence between adjacent pixels in the plain and encrypted images, Pearson’s correlation coefficient was calculated in the horizontal, vertical, and diagonal directions. The coefficient measures the degree of linear relationship between the intensity values of adjacent pixel pairs and is calculated as

(59) $$r_{\mathrm{xy}}=\frac{\sum_{i=1}^{N}\left(x_{i}-\bar{x}\right)\left(y_{i}-\bar{y}\right)}{\sqrt{\sum_{i=1}^{N}{\left(x_{i}-\bar{x}\right)}^{2}\sum_{i=1}^{N}{\left(y_{i}-\bar{y}\right)}^{2}}}$$

where *x_i_* and *y_i_* denote the intensity values of the *i*-th pair of adjacent pixels, $\bar{x}\;$and $\bar{y}\;$represent the corresponding mean intensity values, and *N* is the total number of examined pixel pairs. The coefficient *r_xy_* ranges from −1 to 1. Values close to 1 or −1 indicate a strong positive or negative linear correlation, respectively, whereas values close to zero indicate that the adjacent pixels are statistically uncorrelated. Therefore, for a secure encrypted image, the correlation coefficients in all three directions are expected to approach zero, indicating that the encryption process effectively eliminates the strong spatial dependence present in the plain image.

**Figure 10:**
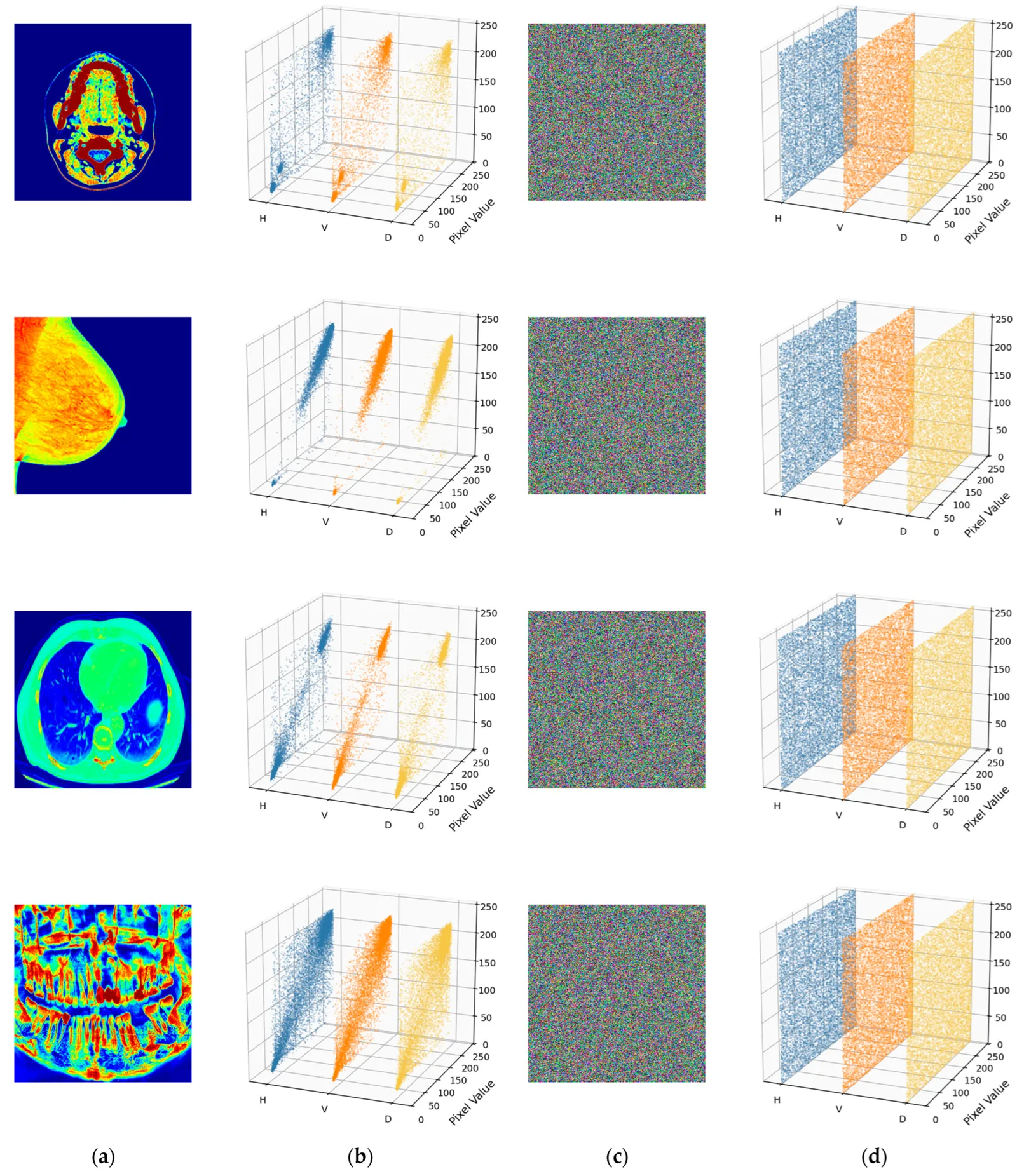
Correlation analysis of adjacent pixels in the plain and encrypted images: (**a**) plain images, (**b**) three-dimensional scatter distributions of horizontally, vertically, and diagonally adjacent pixels in the plain images, (**c**) encrypted images, and (**d**) corresponding scatter distributions of the encrypted images.

As shown in Figure 10, the adjacent pixels of the plain images exhibit highly concentrated and structured distributions in the horizontal (H), vertical (V), and diagonal (D) directions, indicating strong spatial correlation between neighboring pixel values. In contrast, the corresponding distributions of the encrypted images are widely and uniformly dispersed over the entire pixel-value range, without any visible linear or clustered pattern. This substantial disruption of the original correlation structure demonstrates that the proposed encryption method effectively removes the statistical dependence between adjacent pixels. Consequently, the encrypted images do not preserve recognizable spatial information from the plain images, which strengthens their resistance to correlation-based statistical attacks.

As shown in Table 17, the original test images exhibit strong adjacent-pixel dependence in the horizontal, vertical, and diagonal directions, with correlation coefficients ranging from 0.6760 to 0.9964 across the evaluated RGB channels and grayscale representations. After encryption, this spatial dependence is substantially suppressed, and the reported correlation coefficients are concentrated near zero. For the RGB channel averages, the horizontal coefficients range from −0.0079 to 0.0104, the vertical coefficients from −0.0139 to 0.0147, and the diagonal coefficients from −0.0127 to 0.0073. Similarly, the encrypted grayscale images yield horizontal, vertical, and diagonal coefficients within [−0.0174, 0.0280], [−0.0287, 0.0191], and [−0.0233, 0.0301], respectively. Across the individual encrypted RGB channels, the largest absolute correlation coefficient is 0.0436, observed for the vertical correlation of the blue channel of the Dental image; this value nevertheless remains small relative to the strong correlations present in the corresponding plain images. Overall, the results show that the encryption process substantially reduces the linear dependence between adjacent pixels in both natural and medical images, yielding ciphertexts with strongly decorrelated neighboring-pixel values.

Table 18 compares the adjacent-pixel correlation coefficients obtained for the encrypted RGB Lena image with those reported in [42,43,44,45]. The proposed method yields horizontal, vertical, and diagonal correlation coefficients of 0.0104, 0.0147, and 0.0011, respectively. The corresponding coefficients reported by the compared methods range from −0.0073 to 0.0238 in the horizontal direction, from −0.0009 to 0.0149 in the vertical direction, and from −0.0076 to 0.0038 in the diagonal direction. The proposed horizontal and vertical coefficients therefore remain within the ranges reported by the existing methods, while the diagonal coefficient has the smallest absolute magnitude among the compared results. Overall, all three coefficients obtained by the proposed method remain close to zero and are consistent with the low adjacent-pixel correlation levels reported in the literature.

### 4.5. Information Entropy

Information entropy analysis quantitatively evaluates the randomness, uniformity, and unpredictability of pixel-value distributions in encrypted images and is commonly performed using Shannon entropy [46]. For an 8-bit image, the theoretical maximum entropy is 8 bits; therefore, values approaching this limit, typically around 7.99 bits, indicate that the encrypted-image histogram is close to a uniform distribution and that the pixel values are statistically difficult to predict. High entropy is consequently regarded as an important indicator of resistance to statistical analysis and of the unpredictability introduced by the encryption process.

The information entropy is calculated as

(60) $$H=-\sum_{i=1}^{L}p_{i}*\log_{2}\left(p_{i}\right)$$

where *L* denotes the total number of possible intensity levels and *p_i_* represents the occurrence probability of the *i*-th intensity level. For an 8-bit grayscale image or an individual color channel, *L* = 256.

As reported in Table 19, the information entropy values of the original images vary considerably across the RGB channels and grayscale representations, ranging from 3.0882 for the green channel of the Brain image to 7.7526 for the blue channel of the Mandrill image. This variation reflects the different intensity distributions and structural characteristics of the natural and medical test images. After encryption, however, the entropy values become tightly concentrated near the theoretical maximum of 8 bits. Across all encrypted RGB channels and grayscale representations, the entropy values range from 7.9966 to 7.9978. The minimum value of 7.9966 is observed for both the blue channel of Lena and the green channel of Mandrill, whereas the maximum value of 7.9978 is obtained for the red channel of Chest. The average RGB entropy values are similarly confined to a narrow interval of 7.9970–7.9974. Despite the substantial variation among the original images, the encrypted outputs therefore exhibit consistently high entropy and only negligible variation across the evaluated dataset. These results indicate that the encryption process substantially reduces the intensity-level statistical redundancy present in the input images and produces ciphertext distributions with entropy values very close to the theoretical 8-bit maximum.

According to the comparative results presented in Table 20, the proposed method achieves entropy values of 7.9976, 7.9968, and 7.9966 for the red, green, and blue channels of the encrypted Lena image, respectively. Compared to [47], the proposed method yields higher entropy values in all three channels. Relative to [48], the proposed method produces a slightly higher red-channel entropy by 0.0005, while the green- and blue-channel values are lower by 0.0001 and 0.0012, respectively. Despite these small channel-wise differences, all three entropy values of the proposed method remain very close to the theoretical maximum of 8 bits. Overall, the results indicate that the proposed method achieves entropy levels comparable to those reported by [48] and consistently higher than those reported in [47].

### 4.6. Differential Attack Analysis

Differential attack analysis evaluates the sensitivity and diffusion capability of an encryption algorithm by examining the effect of a small modification, typically applied to a single pixel in the plain image, on the encrypted output generated using the same secret key. This analysis is commonly quantified using the Number of Pixels Change Rate (NPCR) and the Unified Average Changing Intensity (UACI). NPCR measures the percentage of pixels whose values differ between two encrypted images produced from plain images that differ only slightly, whereas UACI measures the normalized average intensity difference between the corresponding encrypted pixels [41]. For a secure 8-bit image encryption algorithm, the NPCR value is theoretically expected to approach approximately 99.61%, while the UACI value should be close to 33.46%. These values indicate that a minor change in the plain image produces widespread and substantial changes throughout the encrypted image [40]. Therefore, NPCR and UACI results close to these reference values indicate strong plain-image sensitivity, effective diffusion, and increased resistance to differential and chosen-plaintext attacks.

To quantitatively evaluate the sensitivity of the proposed encryption method to minor plain-image modifications, NPCR and UACI were calculated using two encrypted images generated under the same secret key. The corresponding plain images differed only by a single-pixel modification. NPCR measures the proportion of pixels whose values change between the two encrypted images and is calculated as

(61) $$\mathrm{NPCR}=\frac{\sum_{i,j}D\left(i,j\right)}{M\times N}\times 100\%,$$

where *D*(*i*,*j*) is defined as

$$D\left(i,j\right)=\left\{\begin{array}{cc} 0, & C_{1}\left(i,j\right)=C_{2}\left(i,j\right),\\ 1, & C_{1}\left(i,j\right)\neq C_{2}\left(i,j\right). \end{array}\right.$$

UACI measures the average normalized intensity difference between the corresponding pixels of the two encrypted images and is calculated as

(62) $$\mathrm{UACI}=\frac{1}{M\times N}\sum_{i,j}\frac{\left|C_{1}\left(i,j\right)-C_{2}\left(i,j\right)\right|}{255}\times 100\%.$$

Here, *C*_1_ and *C*_2_ denote the encrypted images obtained from the original and single-pixel-modified plain images, respectively, using the same secret key. *M* and *N* represent the image dimensions, while 255 denotes the maximum intensity value of an 8-bit image. For color images, NPCR and UACI are calculated separately for the red, green, and blue channels.

The differential attack results presented in Table 21 show that a single-pixel modification in the plain image produces widespread changes throughout the corresponding ciphertext for both RGB and grayscale images. Across the individual RGB channels and grayscale experiments, the NPCR values range from 99.5453% for the red channel of the Chest image to 99.6521% for the blue channel of the Chest image, while the UACI values range from 33.1499% for the red channel of Peppers to 33.6253% for the red channel of Mandrill. Across the 11 test images, the mean RGB results are 99.6115% for NPCR and 33.4546% for UACI, whereas the corresponding grayscale means are 99.5999% and 33.4608%, respectively. These values remain close to the theoretical reference levels of approximately 99.61% for NPCR and 33.46% for UACI. Overall, the results indicate that minor plain-image modifications are propagated extensively through the encrypted output across both natural and medical test images.

According to the comparative results presented in Table 22, the proposed method achieves an NPCR of 99.6170% and a UACI of 33.4713% for the encrypted Lena image. These results were compared to the values reported in [49,50,51,52,53,54]. Among the evaluated methods, the proposed scheme yields the highest NPCR, while its UACI remains very close to the theoretical reference level of approximately 33.46%. The compared studies likewise report UACI values concentrated near the theoretical expectation, although [50] shows a noticeably larger deviation in both NPCR and UACI. Overall, the proposed method exhibits differential-response characteristics that are consistent with the expected behavior of an 8-bit encrypted image and comparable to the results reported in the existing literature.

### 4.7. Key-Space and Effective-Key Analysis

The confidential root of the proposed scheme is the 256-bit session secret *K_s_*, independently generated for each encryption instance by a cryptographically secure pseudorandom number generator. The NOAA/SWPC values are used only as public salt/context information and therefore contribute no secret entropy. Likewise, the chaotic initial states, system coefficients, fractional order, nonlinear-feedback parameters, trajectory selectors, DNA controls, and ARX initialization values are deterministic outputs of the HMAC-SHA-512-based HKDF schedule and must not be treated as independent keys. Accordingly, the nominal confidential root-secret domain is

$$\begin{array}{cccc} & |K_{\mathrm{root}}|=2^{256}. & & \end{array}$$

The 512-bit HKDF intermediate values and the multiple domain-separated output fields do not increase the entropy beyond that of the original 256-bit secret. Hence, 2^256^ denotes the nominal input domain rather than a claim that all session secrets necessarily produce numerically distinct operational configurations.

To determine whether finite-precision parameter mapping introduces a smaller effective space, the complete derivation chain *K_s_* → HKDF → *u_f_* → *θ_f_* was analyzed. The main quantization loss occurs in the four 64-bit chaotic initial-state fields. Each such field is mapped to 3 × 2^52^ + 1 distinct binary64 values, corresponding to approximately 53.585 bits of representational capacity and therefore a loss of about 10.415 bits relative to the original 64-bit field. This reduction is mainly caused by the integer-to-binary64 conversion preceding the bounded affine mapping. In contrast, exhaustive evaluation of the eight 16-bit fields controlling *a*, *b*, *c*, *d*, *r*, *α*, *κ*, and *β* showed that all 65,536 possible values remain distinct.

After accounting for these finite-precision effects, the representational capacity of the chaotic initial states and system parameters is approximately

4(53.585) + 8(16) = 342.34 bits.

Including the effective discrete selector, DNA, and ARX control fields increases the complete operational configuration capacity to approximately 647.34 bits. These values are not additional secret entropy and must not be multiplied with the 2^256^ root-secret domain; they only indicate whether the numerical parameterization creates a capacity bottleneck. Since the chaotic-parameter representation alone exceeds 256 bits, the measured quantization loss does not impose a demonstrated bottleneck below the root-secret space.

Equivalent-key behavior was additionally examined at both the derived-configuration and keystream levels. Across 10^6^ distinct 256-bit session secrets, all examined derived configurations were distinct, and no tolerance-equivalent parameter pairs were observed down to the evaluated threshold. However, this finite experiment does not prove injectivity over the full 2^256^ domain; statistically, the zero-collision result excludes only substantially smaller effective spaces, approximately below 2^37.3^ at the 5% level.

A second experiment propagated 10^5^ independently generated session secrets through the complete chaotic generator and produced a 4096-byte keystream for each secret. All resulting keystreams were distinct, with no collisions for prefixes of eight bytes or longer. Short-prefix positive controls produced collision counts consistent with birthday-bound expectations, confirming the operation of the collision detector. Nevertheless, the corresponding statistical power excludes only effective spaces below approximately 2^30.6^; therefore, these results provide empirical evidence against severe many-to-one collapse rather than proof of a 2^256^ effective space.

Finally, numerical-resolution analysis showed that extremely close chaotic states can become equivalent under binary64 integration. In particular, a one-unit-in-the-last-place (1-ULP) perturbation of *x*_0_ was absorbed during numerical evolution and produced an identical evaluated keystream. The measured equivalence widths nevertheless corresponded to estimated chaotic-state capacities of approximately 2^275.0^ for a 4096-byte keystream and 2^325.6^ for a 65,536-byte keystream, both remaining above the 256-bit root-secret domain. Moreover, the smallest normalized parameter-space separation observed among the tested one-bit-related session-secret pairs was 0.363, far above the identified ULP-scale equivalence region.

Taken together, these results distinguish the nominal root-secret space, the representational capacity of the derived numerical configuration, and the exact but analytically unknown effective key space of the complete finite-precision implementation. Finite-precision entropy reduction and local numerical equivalence are present and have been explicitly quantified; however, neither analysis reveals a capacity bottleneck below the 256-bit root-secret domain, and no configuration- or keystream-level collisions were observed in the conducted experiments. Accordingly, 2^256^ is reported in this work strictly as the nominal confidential root-secret space and an upper bound on exhaustive secret search, rather than as a formally proven 2^256^ effective key space.

### 4.8. Analysis of Cropping and Noise Attacks

Cropping and noise attack analyses are standard security evaluation procedures used to assess the robustness of an image encryption system against partial data loss and external distortions. In a cropping attack, a specific region of the encrypted image is intentionally removed or masked, whereas in a noise attack, random distortions such as Gaussian noise or salt-and-pepper noise are added to the encrypted data to simulate transmission errors [55]. These analyses determine whether the encryption algorithm can recover the original image with sufficient quality under real-world conditions involving transmission losses or malicious interference.

**Figure 11:**
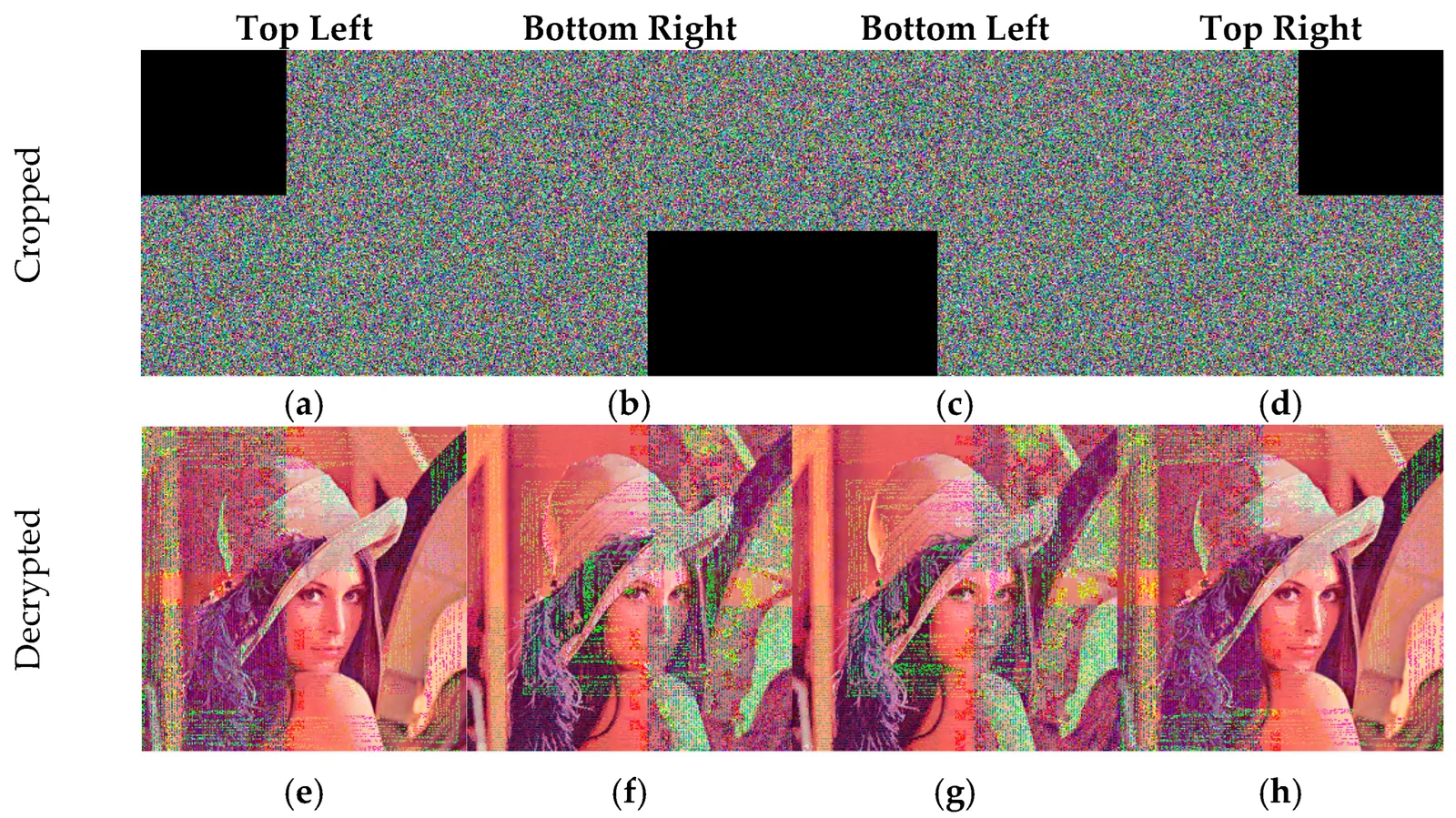
Robustness of the proposed algorithm against a 20% corner-cropping attack on the encrypted Lena image: (**a**–**d**) cropped ciphertexts with the top-left, bottom-right, bottom-left, and top-right corners removed, respectively; (**e**–**h**) corresponding decrypted images obtained from (**a**–**d**), respectively.

As presented in Figure 11, the proposed algorithm maintains acceptable visual recovery performance under a 20% corner-cropping attack applied to the encrypted Lena image. Although the cropped ciphertext regions introduce noticeable distortion, color degradation, and artifact propagation in the decrypted outputs, the main structural content of the image remains visually recognizable in all four cases. The results also show that the impact of cropping depends on the attacked corner; while all attacks degrade the reconstruction quality to some extent, the decrypted image still preserves sufficient perceptual information for scene identification. These findings indicate that the proposed method provides a meaningful degree of robustness against partial ciphertext loss, even though the recovered images are not artifact-free.

**Figure 12:**
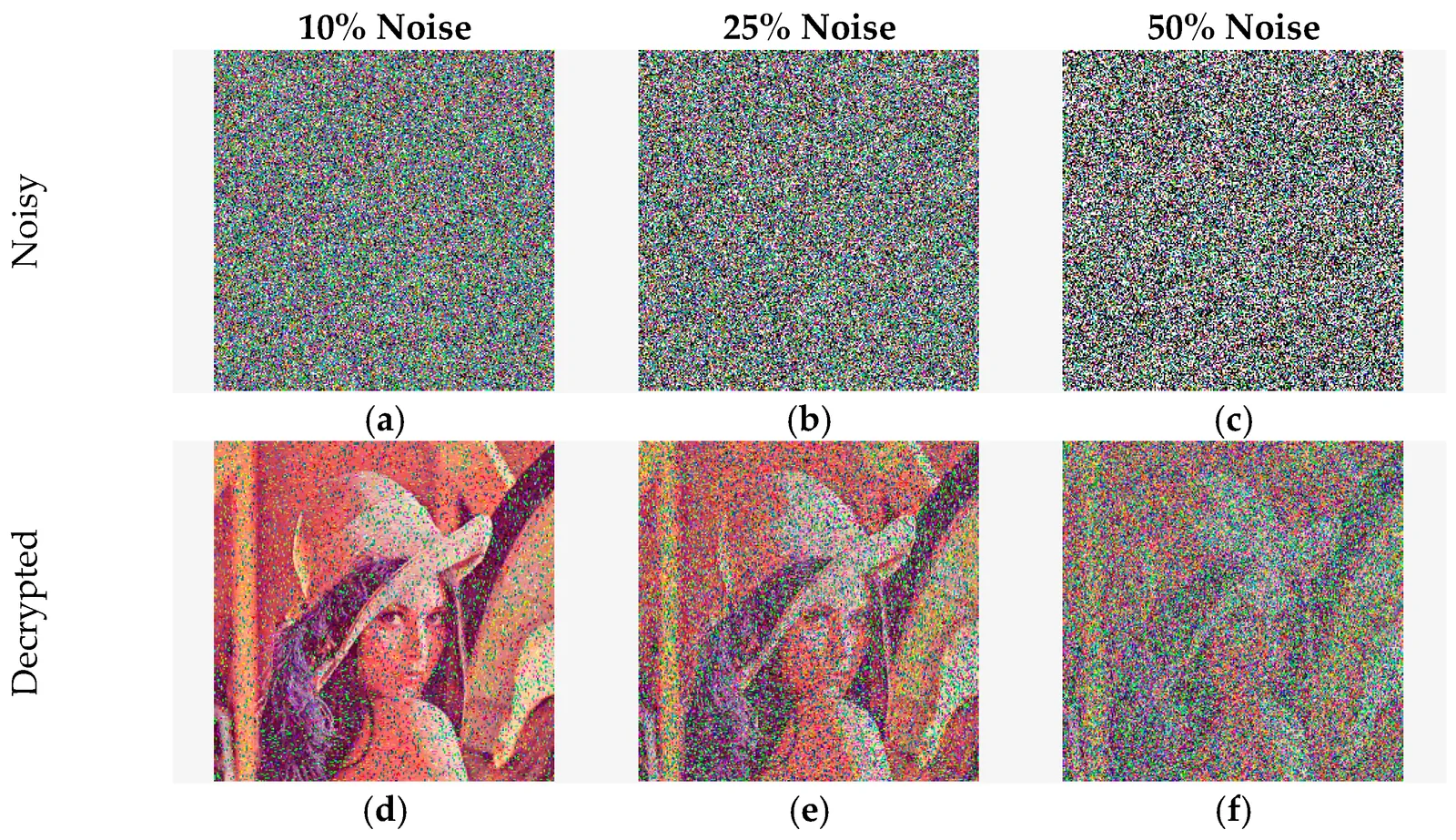
Salt-and-pepper noise attack resilience analysis: (**a**–**c**) encrypted images corrupted with 10%, 25%, and 50% noise levels; (**d**–**f**) corresponding decrypted images.

Figure 12 shows that the proposed algorithm maintains acceptable decryption robustness under 10% and 25% salt-and-pepper noise attacks, where the principal contours and semantic content of the Lena image remain identifiable despite visible artifacts. However, under 50% noise corruption, the decrypted result exhibits substantial visual distortion, indicating that excessive ciphertext contamination weakens the reconstruction capability. Overall, the findings confirm that the proposed algorithm is resistant to moderate impulse-noise attacks, although the recovery quality degrades progressively with increasing noise density.

### 4.9. Known-Plaintext and Chosen-Plaintext Attacks

In a known-plaintext attack, an adversary uses one or more available plain-image–cipher-image pairs to infer the secret key or an equivalent encryption relationship. In a chosen-plaintext attack, the adversary selects specific plain images and analyzes their corresponding cipher images to identify structural weaknesses or reusable relationships in the encryption process [56].

Known-plaintext resistance was evaluated under a same-session configuration rather than relying on session uniqueness. The same 256-bit session secret, public salt, image dimensions, channel mode, and key-schedule context were retained for all evaluated encryptions. A plaintext–ciphertext pair generated from the Lena image was used as the known pair, while Brain and House were selected as independent target plaintexts. Two simple bytewise transfer relations were extracted from the known pair: an XOR-based relation and a modular-additive relation. These relations were subsequently applied to the corresponding target ciphertexts to determine whether information inferred from the known pair could be transferred to reconstruct the target plaintexts.

To quantify the reconstruction quality of the resulting transfer guesses, the exact pixel-match ratio, mean squared error (MSE), peak signal-to-noise ratio (PSNR), structural similarity index (SSIM), and Pearson correlation coefficient were evaluated. A successful transfer would be expected to produce a high exact-match ratio, low MSE, high PSNR and SSIM, and strong correlation with the target plaintext. In contrast, an exact-match ratio close to the random 8-bit coincidence probability of 1/256 (0.390625%), together with low PSNR and SSIM and near-zero correlation, indicates that the inferred bytewise relation does not provide meaningful reconstruction of the target image.

As reported in Table 23, neither transfer relation successfully reconstructs the evaluated target plaintexts. For the Brain image, the exact pixel-match ratios are 0.3677% for the XOR relation and 0.4013% for the modular-additive relation. The corresponding MSE values are 15,921.9637 and 15,917.7872, while the PSNR values are 6.1108 dB and 6.1120 dB, respectively. The SSIM values of 0.0064 and 0.0054, together with correlation coefficients of 0.0030 and 0.0009, indicate negligible structural and linear similarity between the transfer guesses and the actual Brain plaintext. For the House image, the exact pixel-match ratios are 0.3677% and 0.4059%, again remaining close to the random 8-bit coincidence level. The corresponding MSE values are 7706.8451 and 7701.9713, PSNR values are 9.2620 dB and 9.2648 dB, SSIM values are 0.0092 and 0.0120, and the correlation coefficients are −0.0026 and 0.0005. These results likewise show that the transferred relations do not preserve meaningful structural or pixel-level information about the House plaintext.

**Figure 13:**
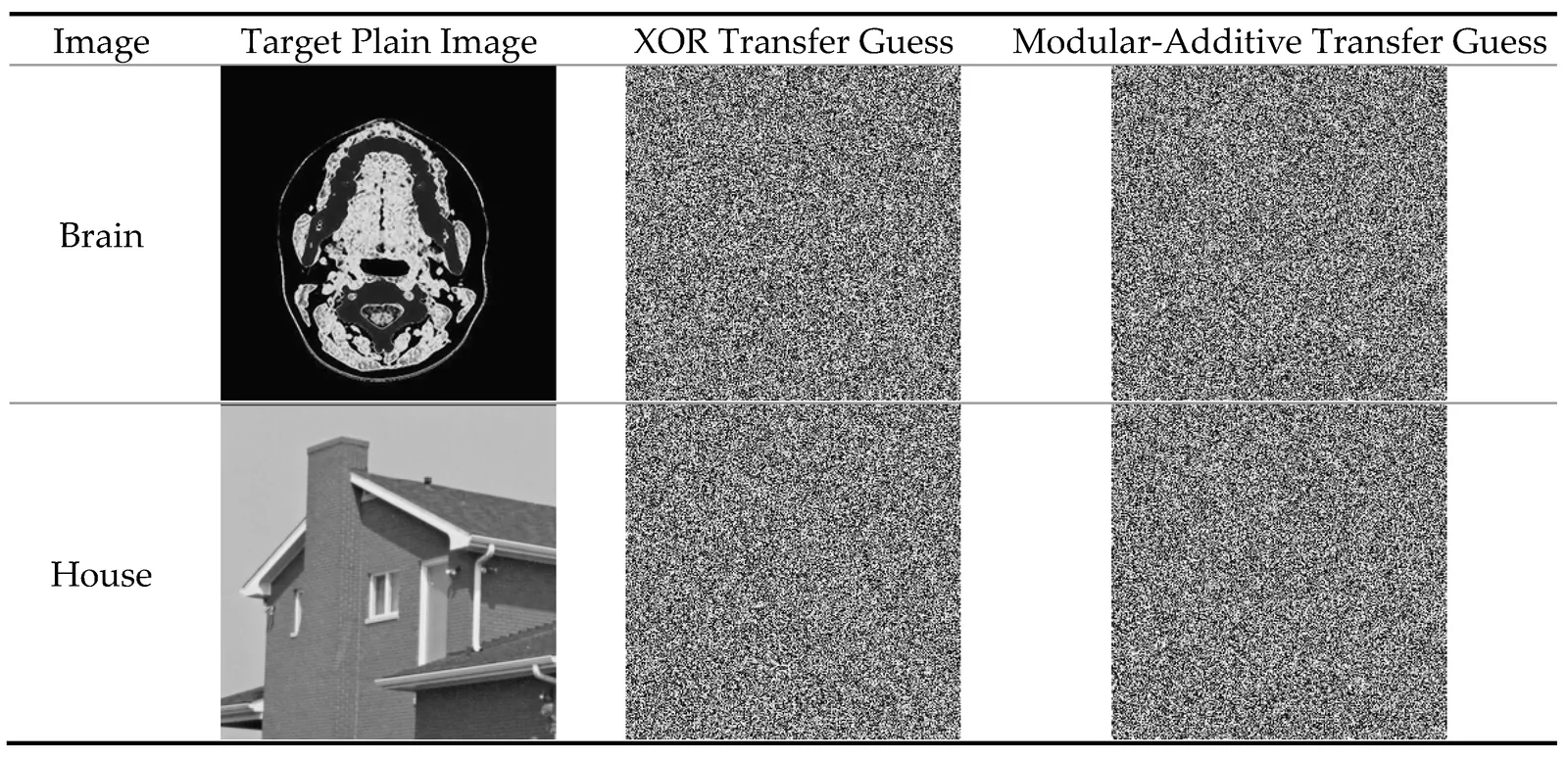
Visual comparison of target plaintexts and the corresponding XOR-based and modular-additive transfer guesses in the same-session known-plaintext analysis.

The visual results presented in Figure 13 are consistent with the quantitative findings. Although the Brain and House target plaintexts contain clearly recognizable anatomical and structural content, both the XOR-based and modular-additive transfer guesses exhibit noise-like patterns with no visually recoverable information from the corresponding target images. Therefore, even when the session secret and schedule context are intentionally held fixed, the simple bytewise relationships extracted from the known plaintext–ciphertext pair do not meaningfully transfer to the evaluated target ciphertexts.

**Figure 14:**
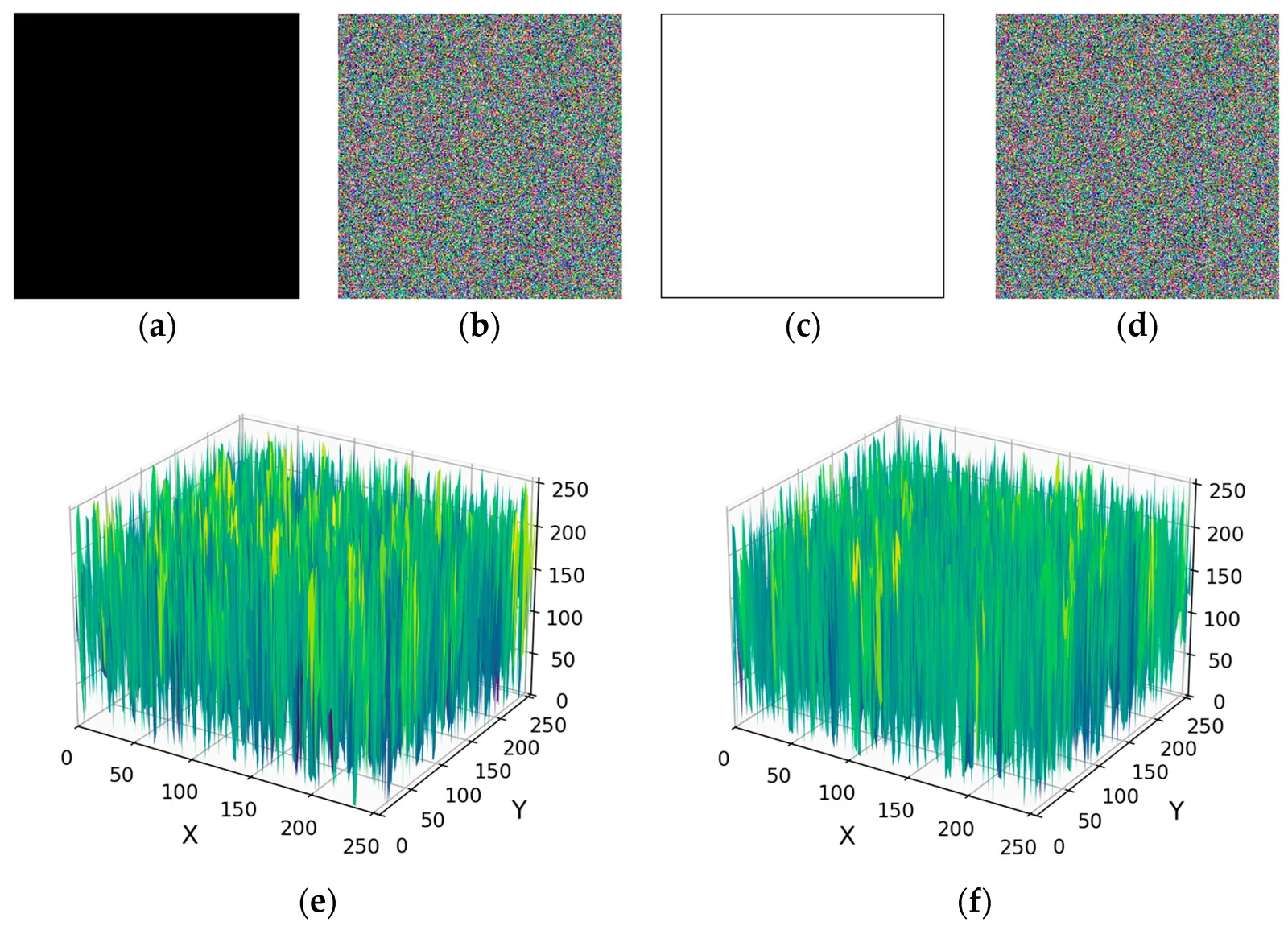
Chosen-plaintext attack analysis using homogeneous images: (**a**) all-black plain image, (**b**) cipher image of (**a**), (**c**) all-white plain image, (**d**) cipher image of (**c**), (**e**) three-dimensional histogram surface of (**b**), and (**f**) three-dimensional histogram surface of (**d**).

As a complementary chosen-plaintext experiment, homogeneous all-black and all-white images were encrypted and examined, as shown in Figure 14. Despite the extreme structural regularity of these inputs, their corresponding ciphertexts exhibit visually noise-like distributions without discernible resemblance to the selected plaintexts. The three-dimensional intensity surfaces likewise show broadly dispersed ciphertext values rather than concentrated structures associated with the homogeneous inputs.

This qualitative experiment provides an initial visual assessment of highly structured homogeneous plaintexts, as shown in Figure 14. However, visual inspection alone is insufficient for a rigorous chosen-plaintext security evaluation. A quantitative structured differential analysis was therefore conducted to complement the qualitative assessment of chosen-plaintext resistance.

The structured differential analysis considered eight plaintext families comprising all-black, all-white, 1 × 1 checkerboard, 8 × 8 checkerboard, horizontal stripes, vertical stripes, a single-pixel impulse, and a sparse impulse pattern. For each plaintext *P*, a corresponding plaintext *P*′ was generated by changing exactly one bit while keeping the 256-bit session secret, public salt, image dimensions, channel mode, and key-schedule context unchanged. Accordingly, the resulting ciphertext difference, Δ*C* = *C*⊕*C*′, reflects the propagation of a controlled plaintext perturbation under an identical encryption configuration. The evaluation included 304 grayscale 256 × 256 pairs, 408 RGB 256 × 256 pairs, and 120 grayscale 512 × 512 pairs, yielding a total of 832 structured differential pairs.

The differential ciphertexts were evaluated using the output bit-change ratio, NPCR, UACI, byte-level differential entropy, chi-square uniformity, adjacent-byte correlation, plaintext–ciphertext differential correlation, and, for RGB images, channel-wise NPCR and UACI. NPCR and UACI were additionally assessed using their corresponding theoretical critical values rather than being interpreted solely according to their proximity to nominal reference values.

As shown in Table 24, the mean output bit-change ratios remain close to the ideal value of 0.5, while the NPCR and UACI values remain close to their theoretical expectations across all evaluated image settings. The observed critical-test pass rates are also broadly consistent with the nominal significance level. These results indicate that the tested structured single-bit plaintext perturbations did not reveal a systematic differential distinguisher.

To further examine response-dependent chosen-plaintext behavior, a separate two-stage adaptive experiment was conducted under the same session secret, public context, image geometry, and encryption configuration. In the first stage, 16 predefined single-bit queries covering the eight plaintext families were evaluated. The plaintext family and bit position exhibiting the largest standardized deviation in the ciphertext bit-change ratio from the theoretical value of 0.5 were then selected according to the observed ciphertext responses. In the second stage, eight previously unqueried neighboring positions around the selected location were evaluated, making the second query set explicitly dependent on the first-stage responses.

The largest first-stage deviation was observed for the all-black/first-LSB case, with a bit-change ratio of 0.501505 (*z* = 2.179). The eight adaptively selected follow-up queries produced a mean bit-change ratio of 0.500092, a mean NPCR of 99.6120%, and a mean UACI of 33.5423%. The individual follow-up bit-ratio *z*-values ranged from −0.655 to 1.643, and the initially selected deviation did not persist in the response-dependent follow-up queries.

Taken together, the same-session known-plaintext transfer analysis, the qualitative homogeneous-plaintext assessment, the structured single-bit differential analysis, and the response-dependent adaptive experiment provide complementary empirical evidence against the evaluated reusable plaintext–ciphertext relations and single-bit differential distinguishers. The results show no systematic differential behavior across the examined structured plaintext families, image formats, or tested perturbation locations under the evaluated configuration. Nevertheless, these experiments constitute empirical cryptanalytic evaluations rather than a formal proof of chosen-plaintext security. More general adaptive strategies, multi-bit and higher-order differentials, truncated or impossible differentials, and key-recovery attacks remain outside the scope of the present analysis.

### 4.10. Ablation Study

A cumulative ablation study was conducted on all 11 RGB test images to determine the functional contribution of each transformation stage to the statistical and differential behavior of the proposed encryption architecture. The evaluation includes seven general-purpose images and four medical images, namely Brain, Breast, Chest, and Dental. This design enables the stage-wise behavior to be examined both over the complete dataset and specifically within the medical-image subset. Table 25 presents the mean information entropy, horizontal, vertical, and diagonal adjacent-pixel correlations, together with the NPCR and UACI values obtained under a one-bit plaintext modification for both evaluation sets. The stages were applied cumulatively so that the incremental contribution of each component to the overall pipeline could be observed.

The plain images exhibit mean entropy values of 6.3773 for the complete dataset and 5.0481 for the medical subset, with adjacent-pixel correlations above 0.87 in all three directions. The Gray-code transformation leaves entropy unchanged, as expected for a bijective re-encoding, but reduces the horizontal, vertical, and diagonal correlations to 0.7824, 0.7836, and 0.7254 for the complete dataset. Its role is therefore that of a reversible preprocessing layer that reorganizes the bit-level structure before bit-plane permutation, rather than an independent diffusion mechanism. In a controlled comparison in which only the Gray-code stage was replaced by the conventional binary representation, with the same session secret, public salt, schedule context, and all other parameters unchanged, both configurations produced final ciphertexts with entropy of approximately 7.997, near-zero correlations, and NPCR/UACI values close to their reference levels. The Gray-coded configuration, however, yielded lower residual adjacent-pixel correlations for several medical images. Gray coding is therefore retained as a modest structural contribution rather than as a principal source of security.

Bit-plane permutation primarily disrupts spatial organization. It reduces the vertical and diagonal correlations to 0.0452 and 0.0484 for the complete dataset, whereas the horizontal correlation remains at 0.7994 and entropy increases only to 6.8654. Position-dependent dynamic DNA mixing subsequently raises entropy to 7.9972 for both evaluation sets and reduces all three correlations below 0.011 in absolute value. Neither stage, however, produces differential propagation: NPCR and UACI remain at their baseline levels, confirming that high entropy and low correlation alone do not imply plaintext sensitivity.

The forward SPARKLE256 duplex introduces the principal differential propagation, raising NPCR and UACI to 99.6100% and 33.4657% for the complete dataset and to 99.6183% and 33.5024% for the medical subset, while preserving near-maximal entropy and near-zero correlations. The inter-pass mask and the backward duplex do not act as independent sources of statistical randomization; they preserve these characteristics while completing the bidirectional diffusion structure. The full algorithm yields NPCR and UACI values of 99.6115% and 33.4546% for the complete dataset and 99.6065% and 33.4517% for the medical subset.

Overall, the stages make distinct and complementary contributions: permutation provides spatial scrambling, dynamic DNA mixing provides value-domain randomization, and duplex diffusion provides differential propagation. The same stage-dependent trends are observed within the medical-image subset.

### 4.11. Computational Complexity and Encryption Speed

To evaluate the computational cost of the proposed algorithm, theoretical time and space complexity were examined together with experimental CPU execution times. Let *M* = *CHW* denote the total number of processed image samples, where *C* is the number of channels, *H* × *W* is the spatial image size, and *B* = 8 is the fixed number of bit planes. The Gray-code transformation, dynamic DNA mixing, and forward/backward ARX diffusion stages each process the image sequence linearly and therefore require *O*(*M*) time. Bit-plane decomposition and trajectory-based permutation require *O*(*BM*) time; however, because *B* = 8 is fixed, this term asymptotically reduces to *O*(*M*). In the full configuration, the fractional-order Chen system generates *L* = 6*M* chaotic bytes. When the Grünwald–Letnikov short-memory window length is denoted by *D*, chaotic-stream generation requires *O*(*LD*) = *O*(*MD*) time. Accordingly, the complete encryption procedure has a general time complexity of *O*(*MD*) and a space complexity of *O*(*M* + *L* + *D*). Under the fixed short-memory configuration used in the experiments, *D* is treated as an implementation parameter independent of image size, so both the computational and memory requirements scale approximately linearly with image size. Table 26 presents the theoretical complexity of each principal stage together with the corresponding pure-Python and optimized CPU execution times.

The fractional-order formulation is intrinsically more expensive than an integer-order recurrence because each Grünwald–Letnikov update accumulates weighted contributions from the *L*_mem_ most recent states. The optimization described below therefore does not alter this formulation but executes it more efficiently.

Stage-wise profiling was performed using the 256 × 256 RGB Lena image on an AMD Ryzen 9 7950X3D 16-core processor. Two implementation configurations were compared. In the pure-Python baseline, fractional-order Chen generation, bit-plane permutation, and dynamic DNA processing were executed using direct Python-based implementations. The optimized configuration employed Numba-compiled execution for fractional-order Chen keystream generation and NumPy-based vectorized execution for bit-plane permutation and dynamic DNA processing. For each configuration, one warm-up run was performed, followed by three complete encryption/decryption runs, and the median execution time was reported. Table 26 presents the execution time of each encryption stage and the corresponding speedup under these two implementations.

The pure-Python results in Table 26 show that the computational cost of the algorithm is highly concentrated in the fractional-order chaotic generator. Complete encryption requires 100,722.174 ms, of which 96,723.822 ms is spent on fractional-order Chen keystream generation. In comparison, bit-plane decomposition and permutation require 2448.768 ms, dynamic DNA value mixing requires 1177.266 ms, Gray-code transformation requires 116.793 ms, forward SPARKLE256 duplex requires 128.214 ms, and backward SPARKLE256 duplex requires 127.311 ms. These measurements identify the iterative evaluation of the fractional-order chaotic trajectory as the dominant computational bottleneck, whereas the remaining image-transformation and bidirectional diffusion stages contribute substantially smaller execution costs.

Under the optimized implementation, the execution cost is reduced substantially across the computationally intensive stages. The Numba-compiled fractional-order Chen generation time decreases from 96,723.822 ms to 935.052 ms, corresponding to a speedup of approximately 103.44×. Bit-plane decomposition and permutation decrease from 2448.768 ms to 22.209 ms, yielding an approximately 110.26× speedup, while dynamic DNA mixing decreases from 1177.266 ms to 66.336 ms, corresponding to approximately 17.75× acceleration. The forward and backward SPARKLE256 duplex stages require 126.834 ms and 125.394 ms, respectively, and therefore remain close to their pure-Python execution times. Overall, the complete encryption time decreases from 100,722.174 ms to 1356.327 ms, corresponding to an overall speedup of approximately 74.26×.

Despite this acceleration, the fractional-order Chen generator still accounts for about 68.9% of the optimized encryption time. The complete 256 × 256 RGB encryption time is reduced from approximately 100.7 s to 1.36 s.

In addition to stage-wise profiling, the behavior of the optimized implementation under increasing image sizes was also evaluated. Resolution scalability was examined across all 11 test images, including the Brain, Breast, Chest, and Dental medical images. The experiments were conducted in both grayscale and RGB modes at resolutions of 256 × 256, 512 × 512, 1024 × 1024, and 2048 × 2048. For each test, one warm-up run was followed by three complete encryption/decryption executions, and the median time was reported. In these experiments, the optimized backend employed Numba for fractional-order Chen generation and NumPy-based execution for permutation and DNA processing. Spatial throughput and processed-sample throughput were evaluated separately because a grayscale spatial pixel contains one 8-bit sample, whereas an RGB spatial pixel contains three separate channel samples. Table 27 presents the mean execution-time and throughput results obtained across all 11 images.

According to Table 27, the mean grayscale encryption time is 0.4426 s at 256 × 256 resolution and increases to approximately 1.7778 s, 7.1339 s, and 28.3945 s at 512 × 512, 1024 × 1024, and 2048 × 2048, respectively. For RGB images, the corresponding mean execution times are 1.3589 s, 5.4223 s, 21.6719 s, and 87.1797 s. Taking the 256 × 256 case as the 1.00× reference, the grayscale encryption times increase by approximately 1.00×, 4.02×, 16.12×, and 64.15×, whereas the RGB times increase by 1.00×, 3.99×, 15.95×, and 64.16×. Because doubling both spatial dimensions increases the total number of pixels by a factor of four, these measured growth factors remain close to the ideal linear progression of 1×, 4×, 16×, and 64×. This observation is consistent with the approximately *O*(*M*) scaling predicted by the complexity analysis when *D* is fixed.

The throughput results further support this scaling behavior. For grayscale encryption, the spatial throughput remains approximately between 0.1470 and 0.1481 MPix/s across the evaluated resolution range, whereas the RGB spatial throughput remains close to 0.048 MPix/s. The processed-sample throughput for RGB images remains within approximately 0.1443–0.1452 MB/s. Thus, although the number of spatial pixels increases by a factor of 64 from 256 × 256 to 2048 × 2048, throughput remains within a relatively narrow range. This indicates that execution time increases approximately in proportion to image size and that the system does not exhibit an abrupt performance collapse at higher resolutions. Moreover, the same behavior is observed across both general-purpose and medical images, supporting the conclusion that the measured scalability profile is not specific to a particular image content.

Nevertheless, absolute execution speed and asymptotic scalability should be considered separately. For standard and relatively small image sizes, the measured execution times remain compatible with latency-tolerant image-transfer scenarios. In particular, 256 × 256 grayscale and RGB images are encrypted in approximately 0.443 s and 1.359 s, respectively, indicating that the current CPU implementation remains applicable to image-based scenarios that do not require sustained high-frame-rate processing. As the image resolution increases, however, the computational cost becomes more restrictive; for example, the mean encryption time reaches approximately 87.2 s for 2048 × 2048 RGB images. Accordingly, the present implementation demonstrates feasibility for standard-resolution image encryption, whereas applications involving sustained high-frame-rate processing, such as real-time video encryption, or large high-resolution RGB workloads would require further acceleration. For such more demanding scenarios, further throughput improvements may be achieved through parallelization of the fractional-order computations, additional optimization of data-processing and memory-access operations, and GPU- or FPGA-based acceleration.

Overall, Table 26 and Table 27 show that implementation-level optimization substantially reduces execution time while the optimized method retains approximately linear scaling with image size under the fixed short-memory configuration.

## 5. Conclusions

This study developed an encryption-instance-specific framework for protecting medical images through a coordinated combination of cryptographic key scheduling, fractional-order hyperchaos, adaptive permutation, position-dependent dynamic DNA transformation, and SPARKLE256-based two-pass ARX-duplex diffusion. A confidential 256-bit session secret generated independently by a CSPRNG is processed with canonically encoded NOAA/SWPC data through an HMAC-SHA-512-based HKDF. The session secret provides the cryptographic entropy, while the publicly accessible NOAA/SWPC information is used only as reproducible temporal context. Domain-separated derivations control the fractional-order modified Chen system, bit-plane trajectory selectors, DNA base-rule selectors, and independently derived forward and backward SPARKLE256 duplex seeds. This organization avoids deriving secret material from the plaintext and prevents the reuse of undifferentiated key material across functionally distinct stages of the cipher.

The dynamical analyses established a numerically validated operating region for the key-controlled fractional-order generator. Boundedness, Lyapunov-spectrum, modified 0–1, NIST SP 800-22, and Dieharder evaluations consistently supported stable hyperchaotic operation and did not reveal broad or persistent statistical anomalies under the tested configurations.

The image-level experiments showed that the encrypted outputs exhibit near-maximal entropy, low adjacent-pixel correlation, and differential responses close to the expected reference levels for 8-bit image encryption. The ablation study further confirmed that the individual transformation stages make distinct and complementary contributions, while implementation-level optimization substantially reduced the computational cost. Despite this improvement, the fractional-order Chen generator remains the principal computational bottleneck.

Several limitations should nevertheless be acknowledged. The experimental evaluation was restricted to selected two-dimensional RGB and grayscale images and did not include complete three-dimensional CT or MR volumes, multi-frame DICOM studies, or clinical PACS workloads. The fractional-order generator was implemented using a numerically validated short-memory Grünwald–Letnikov approximation; consequently, its computational cost and scaling behavior remain important constraints for higher-resolution images and volumetric data. Performance was measured in a CPU-based workstation environment, and the method was not evaluated on embedded, mobile, or resource-constrained PACS clients. Furthermore, although the NOAA/SWPC information is intentionally treated as public context rather than secret entropy, the cryptographic authentication, integrity protection, and replay management of the public context and schedule metadata were outside the scope of this study. Finally, the security assessment is based primarily on dynamical, statistical, differential, robustness, and cryptanalytic experiments; a formal confidentiality proof and implementation-level timing, cache, and power side-channel analyses were not provided.

Future work will systematically address these limitations through further validation and implementation studies. The framework will first be extended to multi-frame DICOM data and complete three-dimensional CT and MR volumes, with explicit measurements of throughput, memory consumption, and reconstruction correctness at increasing spatial and volumetric resolutions. A major implementation objective will be to further reduce the computational burden through stage-specific optimization rather than relying on a single acceleration strategy for the complete pipeline. The fractional-order modified Chen generator, which currently constitutes the dominant computational cost, will be investigated using optimized recurrence evaluation, improved memory handling, compiled numerical kernels, and parallel or segmented execution strategies wherever the temporal dependencies of the short-memory formulation permit. The bit-plane permutation and dynamic DNA stages will be further optimized through SIMD/vectorized execution, cache-aware data layouts, multithreading, batched processing, and GPU-parallel kernels, while the SPARKLE256-based duplex stages will be examined for low-overhead parallel execution across independent blocks, images, or processing batches. Additional improvements will investigate pipeline parallelism, asynchronous execution, overlap of data transfer and computation, and heterogeneous CPU–GPU scheduling so that independent or weakly dependent stages can execute concurrently where possible. The objective is therefore to combine different optimization methods according to the computational characteristics of each stage and thereby reduce the overall encryption cost beyond that achieved by the current Numba- and NumPy-based implementation. FPGA-based and streaming implementations will also be investigated for sustained-throughput scenarios and deployment on PACS gateways and edge medical devices. The public schedule context will be strengthened through cryptographically authenticated metadata or a signed public context source, together with explicit nonce management, integrity protection, and replay-detection mechanisms. Finally, the encryption interface and adversarial model will be formalized to evaluate confidentiality under an IND-CPA-oriented framework, followed by an authenticated-encryption extension and systematic timing, cache, and power side-channel testing.

## Figures and Tables

**Table 1:** DNA encoding rules used in this study.

Binary Value	Rule 1	Rule 2	Rule 3	Rule 4	Rule 5	Rule 6	Rule 7	Rule 8
00	A	A	C	C	G	G	T	T
01	C	G	A	T	A	T	C	G
10	G	C	T	A	T	A	G	C
11	T	T	G	G	C	C	A	A

**Table 2:** DNA exclusive-OR operation used in this study.

⊕_DNA_	A	C	G	T
A	A	C	G	T
C	C	A	T	G
G	G	T	A	C
T	T	G	C	A

**Table 3:** Assignment of selector values to scan trajectories.

Selector *s_k_*	Assigned Trajectory
0	Forward zigzag
1	Reverse zigzag
2	Forward inward spiral
3	Reverse inward spiral
4	Forward Hilbert
5	Reverse Hilbert
6	Row-wise snake
7	Column-wise snake

**Table 4:** Nominal values and key-derived parameter ranges of the fractional-order modified Chen system.

Parameter	Symbol	Nominal Value	Key-Derived Range	HKDF Field Length
Chen coefficient	*a*	35	[34.30, 35.70]	2 bytes
Chen coefficient	*b*	3	[2.94, 3.06]	2 bytes
Chen coefficient	*c*	12	[11.76, 12.24]	2 bytes
Chen coefficient	*d*	7	[6.86, 7.14]	2 bytes
Feedback control	*r*	0.5	[0.49, 0.51]	2 bytes
Fractional order	α	—	[0.94, 1.00)	2 bytes
Feedback gain	κ	—	[0.05, 0.50]	2 bytes
Feedback sensitivity	β	—	[0.0005, 0.0050]	2 bytes
Initial state	*x*_0_, *y*_0_, *z*_0_, *w*_0_	—	[−1, 1] each	32 bytes

**Table 5:** Unique equilibrium of the fractional-order modified Chen system and the eigenvalues of the associated Jacobian at the nominal coefficients. The eigenvalues are independent of the feedback parameters κ and β.

Equilibrium	Coordinates	λ_1_	λ_2_	λ_3_	λ_4_	Type
O	(0, 0, 0, 0)	−39.7356	+16.7356	−3.0000	+0.5000	Unstable saddle

**Table 6:** Three-stage bisection scan for the lower bound of the admissible fractional order. A configuration is counted as passing only when the chaos criterion is satisfied under both integrator settings.

Stage	Range of α	Step Δα	Orders	Long Runs	Fully Robust Orders
Coarse	0.880–0.940	10^−3^	61	610	6
Refined	0.9360–0.9390	10^−4^	31	310	29
Ultra-refined	0.93700–0.93750	10^−5^	51	510	46
Upper band	0.940–0.999	10^−3^	60	600	60

**Table 7:** Median values of the four largest Lyapunov exponents of the short-memory GL companion map over the admissible (κ, β) rectangle, reported per sampled fractional order (100 grid points per order, 10^6^ measurement steps). Exponents are expressed per unit time.

α	λ_1_	λ_2_	λ_3_	λ_4_
0.9400	+0.2504	+0.1244	−0.0022	−29.2699
0.9466	+0.2932	+0.1395	−0.0033	−28.9736
0.9531	+0.3299	+0.1480	−0.0028	−28.6662
0.9597	+0.3517	+0.1538	−0.0020	−28.3513
0.9662	+0.3823	+0.1530	−0.0021	−28.0347
0.9728	+0.4153	+0.1478	−0.0026	−27.7194
0.9793	+0.4508	+0.1367	−0.0026	−27.3935
0.9859	+0.4870	+0.1168	−0.0027	−27.0674
0.9924	+0.5310	+0.0948	−0.0034	−26.7496
0.9990	+0.5719	+0.0811	−0.0044	−26.4347

**Table 8:** Validation of the Lyapunov estimator against the analytic divergence ∇·F = −25.5 at α = 1, κ = 0. The upper block reports the full spectrum at the production step size; the lower block reports the relative deviation under step-size refinement at matched physical horizon.

Quantity	Value
λ_1_	+0.562441
λ_2_	+0.093239
λ_3_	−0.005553
λ_4_	−26.391313
Σλ_i_ (measured)	−25.741185
∇·F (analytic)	−25.500000
Kaplan–Yorke dimension *D*_KY_	3.0246
Relative deviation at *h* = 1.0 × 10^−3^	0.9471%
Relative deviation at *h* = 5.0 × 10^−4^	0.4698%
Relative deviation at *h* = 2.5 × 10^−4^	0.2334%
Successive ratios (theoretical value 0.5)	0.4960/0.4968

**Table 9:** Modified 0–1 chaos test over the admissible region, with a periodic sinusoidal negative control.

Group	Cases	Median K (Minimum over Cases)	Minimum Individual-*c*_test_ Statistic	Accepted
Fractional modified Chen	35	0.9971	0.8759	35/35
Periodic sinusoidal control	1	−0.0019	—	0/1

**Table 10:** Full factorial convergence campaign over integration step and retained memory length. Bit balance and lag-one autocorrelation are reported as medians and worst cases over the 25 configurations evaluated in each cell.

*h*	*L*_mem_	*L*_mem_·*h*	Bounded	Bit Balance (Median)	max|ACF(1)|
5 × 10^−4^	50	0.025	25/25	0.49982	0.744
5 × 10^−4^	100	0.050	25/25	0.50012	0.005
5 × 10^−4^	200	0.100	25/25	0.50011	0.008
5 × 10^−4^	500	0.250	25/25	0.49994	0.007
1 × 10^−3^	50	0.050	25/25	0.49994	0.009
1 × 10^−3^ (production)	100	0.100	25/25	0.49990	0.006
1 × 10^−3^	200	0.200	25/25	0.49981	0.010
1 × 10^−3^	500	0.500	15/25	0.49948	0.722
2 × 10^−3^	50	0.100	25/25	0.49993	0.008
2 × 10^−3^	100	0.200	25/25	0.50026	0.006
2 × 10^−3^	200	0.400	25/25	0.50001	0.007
2 × 10^−3^	500	1.000	21/25	0.49992	0.723

**Table 11:** Exploratory widening of the feedback gain, evaluated on a separate branch. Each row reports a 10 × 10 × 10 Lyapunov grid measured against a freshly computed control grid at the production gain range under identical settings. The effect is quantified within fixed α slices to remove the dominant influence of the fractional order.

Gain Range	Order Band	Relative Effect on λ_2_	Sign Consistency	Effect/Control	Partial Correlation (κ, λ_2_|α)	λ_2_ > 0
[0.05, 0.5] (production)	[0.94, 1)	3.6%	5/10	—	−0.015	100%
[5, 50]	[0.94, 1)	19.5%	10/10	7.49×	−0.295	99.6%
[5, 30]	[0.94, 1)	9.5%	10/10	5.71×	—	99.8%
[5, 35]	[0.95, 1)	11.6%	10/10	9.47×	−0.286	100%

**Table 12:** Functional assignment of the DNA value and rule streams.

Symbol	Source	Functional Role	Length
*Q*_DNA_	Fractional modified Chen stream	DNA operand paired with *P*	*M* bytes
${\bar{R}}_{I},{\bar{R}}_{K},{\bar{R}}_{O}$	HKDF DNA-rule field	Base offsets for image, chaotic, and output rules	3 bytes
*R_I_*	Non-overlapping chaotic segment	Position-dependent image-rule selector	*M* bytes
*R_K_*	Non-overlapping chaotic segment	Position-dependent chaotic-rule selector	*M* bytes
*R_O_*	Non-overlapping chaotic segment	Position-dependent output-rule selector	*M* bytes

**Table 13:** Assignment of key-schedule and diffusion quantities in the SPARKLE256-based two-pass ARX-duplex diffusion.

Quantity	Derivation or Source	Length/Range	Functional Role
$Q_{\mathrm{DIF}}^{\left(F\right)}$	First half of the 2*M*-byte diffusion segment generated by the fractional-order modified Chen system	*M* bytes	Keystream absorbed into the rate during the forward duplex pass
$Q_{\mathrm{DIF}}^{\left(B\right)}$	Second half of the 2*M*-byte diffusion segment	*M* bytes	Independent keystream absorbed into the rate during the backward duplex pass
Seed*_F_* = *η*^(*SF*)^	HKDF-Expand output derived under info*_SF_*	16 bytes	Initializes the forward 256-bit duplex state
Seed*_B_* = *η*^(*SB*)^	HKDF-Expand output derived under info*_SB_*	16 bytes	Initializes the backward 256-bit duplex state
Λ	Mask_SP_ (Fold(*U*), Seed*_F_*⊕Seed*_B_*, *n_b_*)	16 bytes	Global inter-pass mask applied to every intermediate block before the backward duplex pass

**Table 14:** NIST SP 800-22 subtest-row results for the proposed outputs and the AES-256-CTR reference.

Test Name	Rows	Modified Chen Keystream	Ciphertext	AES-256-CTR
Frequency	1	1/1	1/1	1/1
Block Frequency	1	1/1	1/1	1/1
Cumulative Sums	2	2/2	2/2	2/2
Runs	1	1/1	1/1	1/1
Longest Run	1	1/1	1/1	1/1
Binary Matrix Rank	1	1/1	1/1	1/1
Discrete Fourier Transform	1	1/1	1/1	1/1
Non-overlapping Template	148	147/148	145/148	144/148
Overlapping Template	1	1/1	1/1	1/1
Maurer’s Universal Statistical	1	1/1	1/1	1/1
Approximate Entropy	1	1/1	1/1	1/1
Random Excursions	8	8/8	8/8	8/8
Random Excursions Variant	18	18/18	18/18	18/18
Serial	2	2/2	2/2	2/2
Linear Complexity	1	1/1	1/1	1/1
Total	188	187/188	185/188	184/188

**Table 15:** Summary of the Dieharder statistical test results.

Output Group	Total Rows	Overall P/W/F	Rows with Np ≥ 10	P/W/F for Np ≥ 10	Rewind Rows
Fractional modified Chen keystream	111	109/2/0	111	109/2/0	0
Operational ciphertext	111	106/5/0	111	106/5/0	0
AES-256-CTR reference	111	108/3/0	111	108/3/0	0

**Table 16:** Chi-square (*χ*^2^) test results for the encrypted RGB and grayscale images at the *α* = 0.05 significance level and *df* = 255. A result is considered to pass when *χ*^2^ < 293.247.

Image (RGB)	χ^2^	Remarks	Image (Grayscale)	χ^2^	Remarks
Airplane	238.7083	Passed	Airplane	259.8047	Passed
Brain	263.9063	Passed	Brain	248.0938	Passed
Breast	249.0052	Passed	Breast	254.2266	Passed
Chest	242.2161	Passed	Chest	256.5859	Passed
Dental	248.8229	Passed	Dental	272.9609	Passed
House	258.4531	Passed	House	271.0391	Passed
Lena	272.9089	Passed	Lena	237.1797	Passed
Mandrill	265.4349	Passed	Mandrill	266.1094	Passed
Peppers	238.1849	Passed	Peppers	227.0625	Passed
Sailboat on Lake	264.0938	Passed	Sailboat on Lake	264.6719	Passed
Tree	264.3151	Passed	Tree	250.2578	Passed

**Table 17:** Adjacent-pixel correlation coefficients of the plain and encrypted test images in the horizontal, vertical, and diagonal directions for individual RGB channels, their channel averages, and the grayscale representations.

		Original Image			Encrypted Image		
Image	Channel	Horizontal	Vertical	Diagonal	Horizontal	Vertical	Diagonal
Airplane	R	0.9059	0.8852	0.8254	−0.0038	0.0032	0.0171
	G	0.8951	0.8998	0.8394	−0.0172	0.0081	0.0079
	B	0.9212	0.8660	0.8342	0.0263	−0.0195	−0.0294
	Average	0.9074	0.8837	0.8330	0.0018	−0.0027	−0.0015
	Grayscale	0.9020	0.8936	0.8385	0.0028	−0.0287	0.0033
Brain	R	0.9085	0.9333	0.8670	−0.0066	−0.0085	0.0127
	G	0.8973	0.9196	0.8553	0.0001	−0.0171	−0.0069
	B	0.8113	0.8611	0.7308	0.0231	0.0005	0.0077
	Average	0.8723	0.9046	0.8177	0.0055	−0.0083	0.0045
	Grayscale	0.9183	0.9365	0.8821	0.0068	0.0086	−0.0215
Breast	R	0.9958	0.9964	0.9945	−0.0006	0.0039	−0.0035
	G	0.9839	0.9796	0.9783	−0.0044	0.0060	0.0053
	B	0.9773	0.9833	0.9756	−0.0018	0.0209	−0.0025
	Average	0.9857	0.9865	0.9828	−0.0023	0.0102	−0.0002
	Grayscale	0.9903	0.9884	0.9874	0.0252	−0.0071	0.0301
Chest	R	0.9368	0.9590	0.9109	−0.0078	0.0072	−0.0095
	G	0.9743	0.9819	0.9564	0.0180	−0.0225	0.0007
	B	0.8968	0.9162	0.8404	0.0116	−0.0167	−0.0064
	Average	0.9359	0.9524	0.9026	0.0073	−0.0107	−0.0051
	Grayscale	0.9752	0.9815	0.9568	−0.0126	−0.0275	−0.0050
Dental	R	0.8544	0.9147	0.8071	0.0261	0.0112	0.0080
	G	0.8002	0.8702	0.7385	−0.0184	−0.0180	−0.0087
	B	0.8520	0.9132	0.8067	−0.0005	0.0436	0.0131
	Average	0.8355	0.8994	0.7841	0.0024	0.0123	0.0041
	Grayscale	0.8282	0.8896	0.7741	0.0010	0.0042	0.0113
House	R	0.9632	0.9351	0.9201	−0.0126	−0.0040	−0.0057
	G	0.9793	0.9505	0.9247	0.0269	−0.0012	−0.0068
	B	0.9805	0.9748	0.9636	0.0170	−0.0096	−0.0255
	Average	0.9744	0.9535	0.9361	0.0104	−0.0049	−0.0127
	Grayscale	0.9764	0.9541	0.9339	0.0177	0.0191	−0.0180
Lena	R	0.9522	0.9757	0.9307	−0.0044	0.0115	−0.0114
	G	0.9348	0.9675	0.9111	0.0068	−0.0001	0.0180
	B	0.9182	0.9507	0.8844	0.0287	0.0326	−0.0033
	Average	0.9351	0.9646	0.9088	0.0104	0.0147	0.0011
	Grayscale	0.9381	0.9690	0.9142	−0.0106	−0.0077	−0.0173
Mandrill	R	0.9167	0.8734	0.8477	0.0129	−0.0253	−0.0264
	G	0.8043	0.7632	0.6938	0.0064	−0.0129	0.0094
	B	0.8776	0.8733	0.8094	−0.0143	−0.0036	0.0279
	Average	0.8662	0.8367	0.7836	0.0017	−0.0139	0.0036
	Grayscale	0.8061	0.7506	0.6760	−0.0174	−0.0058	−0.0015
Peppers	R	0.9496	0.9511	0.9060	0.0080	−0.0011	0.0011
	G	0.9568	0.9679	0.9232	0.0130	−0.0016	0.0179
	B	0.9367	0.9507	0.9005	0.0017	−0.0061	0.0031
	Average	0.9477	0.9566	0.9099	0.0075	−0.0029	0.0073
	Grayscale	0.9483	0.9605	0.9092	0.0280	−0.0041	−0.0233
Sailboat on lake	R	0.9329	0.9317	0.9001	−0.0116	−0.0127	−0.0055
	G	0.9334	0.9310	0.8924	−0.0095	0.0245	−0.0087
	B	0.9404	0.9457	0.9110	−0.0026	0.0284	0.0017
	Average	0.9355	0.9361	0.9012	−0.0079	0.0134	−0.0041
	Grayscale	0.9370	0.9356	0.9005	0.0075	0.0047	0.0046
Tree	R	0.9586	0.9303	0.9179	−0.0003	−0.0088	−0.0031
	G	0.9659	0.9431	0.9339	0.0117	0.0175	−0.0162
	B	0.9594	0.9371	0.9275	−0.0166	−0.0228	0.0025
	Average	0.9613	0.9368	0.9264	−0.0017	−0.0047	−0.0056
	Grayscale	0.9661	0.9415	0.9321	0.0082	0.0049	0.0049

**Table 18:** Comparison of horizontal, vertical, and diagonal adjacent-pixel correlation coefficients for the encrypted RGB Lena image with existing methods.

	Horizontal	Vertical	Diagonal
Proposed	0.0104	0.0147	0.0011
[42]	0.0238	0.0044	0.0038
[43]	−0.0073	0.0091	−0.0027
[44]	−0.0015	−0.0009	−0.0076
[45]	−0.0045	0.0149	−0.0033

**Table 19:** Information entropy values of the plain and encrypted RGB and grayscale test images.

Image	Channel	Original Image	Encrypted Image
Airplane	R	6.8567	7.9975
	G	6.9564	7.9973
	B	6.3351	7.9973
	Average	6.7161	7.9974
	Grayscale	6.8615	7.9971
Brain	R	3.6695	7.9969
	G	3.0882	7.9974
	B	4.0981	7.9970
	Average	3.6186	7.9971
	Grayscale	3.8238	7.9973
Breast	R	3.4264	7.9971
	G	4.2903	7.9977
	B	3.5466	7.9969
	Average	3.7544	7.9973
	Grayscale	4.1769	7.9972
Chest	R	6.0809	7.9978
	G	4.8756	7.9974
	B	7.1350	7.9969
	Average	6.0305	7.9973
	Grayscale	6.3423	7.9972
Dental	R	6.6483	7.9972
	G	6.6695	7.9974
	B	7.0490	7.9973
	Average	6.7889	7.9973
	Grayscale	7.5729	7.9970
House	R	6.4311	7.9969
	G	6.5389	7.9974
	B	6.2320	7.9972
	Average	6.4007	7.9972
	Grayscale	6.4963	7.9970
Lena	R	7.2495	7.9976
	G	7.5889	7.9968
	B	6.9289	7.9966
	Average	7.2558	7.9970
	Grayscale	7.4417	7.9974
Mandrill	R	7.6756	7.9977
	G	7.4793	7.9966
	B	7.7526	7.9970
	Average	7.6358	7.9971
	Grayscale	7.3500	7.9971
Peppers	R	7.3449	7.9973
	G	7.5607	7.9977
	B	7.1003	7.9971
	Average	7.3353	7.9974
	Grayscale	7.6218	7.9975
Sailboat on lake	R	7.3207	7.9971
	G	7.6720	7.9971
	B	7.3042	7.9971
	Average	7.4323	7.9971
	Grayscale	7.5239	7.9971
Tree	R	7.2104	7.9971
	G	7.4136	7.9973
	B	6.9207	7.9969
	Average	7.1816	7.9971
	Grayscale	7.3103	7.9972

**Table 20:** Comparison of channel-wise information entropy values for the encrypted RGB Lena image with existing methods.

	Red (R)	Green (G)	Blue (B)
Proposed Method	7.9976	7.9968	7.9966
[47]	7.9837	7.9916	7.9550
[48]	7.9971	7.9969	7.9978

**Table 21:** NPCR and UACI results obtained for the encrypted RGB and grayscale test images under a single-pixel plain-image modification.

	Channel	UACI	NPCR
Airplane	R	33.4454	99.6506
	G	33.5238	99.6231
	B	33.3389	99.5605
	Average	33.4360	99.6114
	Grayscale	33.4967	99.6124
Brain	R	33.3695	99.6277
	G	33.4959	99.6353
	B	33.3338	99.5941
	Average	33.3997	99.6190
	Grayscale	33.3525	99.5941
Breast	R	33.5571	99.5972
	G	33.3862	99.6155
	B	33.3647	99.6078
	Average	33.4360	99.6068
	Grayscale	33.4436	99.5926
Chest	R	33.4631	99.5453
	G	33.5973	99.5758
	B	33.4247	99.6521
	Average	33.4950	99.5911
	Grayscale	33.5047	99.5758
Dental	R	33.4893	99.6109
	G	33.4873	99.5972
	B	33.4511	99.6185
	Average	33.4759	99.6089
	Grayscale	33.3230	99.5941
House	R	33.6075	99.6109
	G	33.4905	99.6338
	B	33.5044	99.6078
	Average	33.5341	99.6175
	Grayscale	33.4769	99.6017
Lena	R	33.4035	99.6063
	G	33.5408	99.6368
	B	33.4697	99.6078
	Average	33.4713	99.6170
	Grayscale	33.3788	99.6002
Mandrill	R	33.6253	99.6231
	G	33.4668	99.6292
	B	33.3478	99.6490
	Average	33.4800	99.6338
	Grayscale	33.5079	99.6017
Peppers	R	33.1499	99.5941
	G	33.4139	99.6002
	B	33.4756	99.6002
	Average	33.3464	99.5982
	Grayscale	33.5502	99.5895
Sailboat on lake	R	33.5194	99.6017
	G	33.3859	99.6185
	B	33.2742	99.6048
	Average	33.3932	99.6084
	Grayscale	33.5342	99.6109
Tree	R	33.4909	99.6368
	G	33.4924	99.5911
	B	33.6166	99.6170
	Average	33.5333	99.6150
	Grayscale	33.4999	99.6262

**Table 22:** Comparison of NPCR and UACI values for the encrypted Lena image with existing methods.

	NPCR	UACI
Proposed Method	99.6170	33.4713
[49]	99.6122	33.4868
[50]	97.3967	32.4667
[51]	99.6109	33.4458
[52]	99.6116	33.4670
[53]	99.6145	33.4403
[54]	99.6013	33.4131

**Table 23:** Quantitative known-plaintext transfer analysis under an identical session secret and schedule context.

Target	Transfer Relation	Exact Pixel Match (%)	MSE	PSNR (dB)	SSIM	Correlation
Brain	XOR	0.3677	15,921.9637	6.1108	0.0064	0.0030
Brain	Modular Additive	0.4013	15,917.7872	6.1120	0.0054	0.0009
House	XOR	0.3677	7706.8451	9.2620	0.0092	−0.0026
House	Modular Additive	0.4059	7701.9713	9.2648	0.0120	0.0005

**Table 24:** Quantitative structured chosen-plaintext differential analysis of the proposed method.

	Number of Pairs	Mean Bit-Change Ratio	Mean NPCR (%)	Mean UACI (%)	NPCR Critical-Test Pass Rate	UACI Critical-Test Pass Rate
Grayscale, 256 × 256	304	0.500032	99.6119	33.4712	95.4%	95.4%
RGB, 256 × 256	408	0.500012	99.6083	33.4668	93.6%	96.1%
Grayscale, 512 × 512	120	0.500094	99.6107	33.4596	95.8%	91.7%

**Table 25:** Stage-wise ablation results averaged over the complete dataset and the medical-image subset.

	Evaluation Set	Entropy	Horizontal	Vertical	Diagonal	NPCR (%)	UACI (%)
Plain Image	All	6.3773	0.9234	0.9282	0.8806	0.0005	0.0000
	Medical	5.0481	0.9074	0.9357	0.8718	0.0005	0.0000
Gray Code	All	6.3773	0.7824	0.7836	0.7254	0.0005	0.0000
	Medical	5.0481	0.8064	0.8290	0.7615	0.0005	0.0000
Bit-Plane Permutation	All	6.8654	0.7994	0.0452	0.0484	0.0005	0.0000
	Medical	5.7559	0.7957	0.0744	0.0776	0.0005	0.0000
Dynamic DNA Mixing	All	7.9972	0.0073	0.0042	−0.0014	0.0005	0.0000
	Medical	7.9972	0.0105	0.0024	−0.0012	0.0005	0.0000
Forward SPARKLE256 Duplex	All	7.9972	−0.0018	0.0001	0.0022	99.6100	33.4657
	Medical	7.9971	−0.0033	0.0041	0.0073	99.6183	33.5024
Inter-pass Mask	All	7.9972	−0.0010	0.0030	0.0021	99.6056	33.4587
	Medical	7.9973	−0.0070	0.0032	−0.0037	99.6028	33.4881
Full Algorithm	All	7.9972	0.0032	0.0002	−0.0008	99.6115	33.4546
	Medical	7.9972	0.0032	0.0009	0.0008	99.6065	33.4517

**Table 26:** Theoretical complexity and stage-wise CPU execution cost of the proposed encryption algorithm.

Stage	Time Complexity	Space Complexity	Pure Python (ms)	Optimized (ms)	Speedup
Gray-code transformation	*O*(*M*)	*O*(*M*)	116.793	80.502	1.45×
Bit-plane decomposition and permutation	*O*(*BM*) *→ O*(*M*)	*O*(*BM*)	2448.768	22.209	110.26×
Fractional-order Chen keystream generation	*O*(*LD*) = *O*(*MD*)	*O*(*L* + *D*)	96,723.822	935.052	103.44×
Dynamic DNA value mixing	*O*(*M*)	*O*(*M*)	1177.266	66.336	17.75×
Forward SPARKLE256 duplex	*O*(*M*)	*O*(*M*)	128.214	126.834	1.01×
Backward SPARKLE256 duplex	*O*(*M*)	*O*(*M*)	127.311	125.394	1.02×
Total	*O*(*MD*); fixed *D*:*O*(*M*)	*O*(*M* + *L* + *D*)	100,722.174	1356.327	74.26×

**Table 27:** Mean resolution scalability of the optimized implementation across all 11 test images.

Resolution	Mode	Encrypt (s)	Decrypt (s)	Encrypt Spatial Throughput (MPix/s)	Encrypt Sample Throughput (MB/s)	Relative Encryption Time
256 × 256	Grayscale	0.4426	0.4442	0.1481	0.1481	1.000×
512 × 512	Grayscale	1.7778	1.7881	0.1475	0.1475	4.016×
1024 × 1024	Grayscale	7.1339	7.1518	0.1470	0.1470	16.117×
2048 × 2048	Grayscale	28.3945	28.7574	0.1477	0.1477	64.151×
256 × 256	RGB	1.3589	1.3631	0.0482	0.1447	1.000×
512 × 512	RGB	5.4223	5.4541	0.0483	0.1450	3.990×
1024 × 1024	RGB	21.6719	21.8294	0.0484	0.1452	15.948×
2048 × 2048	RGB	87.1797	87.5857	0.0481	0.1443	64.156×

## Data Availability

The Medical Imaging (CT-Xray) Colorization dataset employed in this study’s experimental evaluation is openly accessible via Kaggle (https://www.kaggle.com/datasets/shuvokumarbasak2030/medical-imaging-ct-xray-colorization-new-dataset, accessed on 1 May 2026). Additional test images used for evaluation purposes were obtained from publicly available standard benchmark image sets.
